# The relationship between sleep and salivary and serum inflammatory biomarkers in adolescents

**DOI:** 10.3389/fmed.2023.1175483

**Published:** 2023-05-26

**Authors:** Hend Alqaderi, Abeer Abdullah, Matthew Finkelman, Mohamed Abufarha, Sriraman Devarajan, Jehad Abubaker, Nikitha Ramesh, Mary Tavares, Fahd Al-Mulla, Saadoun Bin-Hasan

**Affiliations:** ^1^Dasman Diabetes Institute, Dasman, Kuwait; ^2^Department of Oral Health Policy and Epidemiology, Harvard School of Dental Medicine, Boston, MA, United States; ^3^Department of Preventive Dental Sciences, Faculty of Dentistry, King Abdulaziz University, Jeddah, Saudi Arabia; ^4^Department of Public Health and Community Service, Tufts University School of Dental Medicine, Boston, MA, United States; ^5^Boston University School of Public Health, Boston, MA, United States; ^6^Department of Health Policy and Health Services Research, Boston University Henry M. Goldman School of Dental Medicine, Boston, MA, United States; ^7^Farwaniya Hospital, Ministry of Health, Al Farwaniyah, Kuwait

**Keywords:** sleep, cytokin, saliva, serum, inflammation

## Abstract

**Objectives:**

Poor sleep behavior can trigger an inflammatory response and contribute to the development of inflammatory diseases. Cytokines can act as indicators of inflammation and may precede the onset of inflammatory diseases. This study aimed to determine the association between sleep timing parameters (bedtime, sleep duration, sleep debt, and social jetlag) and the levels of nine serum and salivary inflammatory and metabolic biomarkers.

**Methods:**

Data were collected from 352 adolescents aged 16–19 years enrolled in Kuwait’s public high schools. The levels of C-reactive protein (CRP), interleukin-6 (IL-6), interleukin-8 (IL-8), interleukin-10 (IL-10), vascular endothelial growth factor (VEGF), monocyte chemoattractant protein-1 (MCP-1), adiponectin, leptin, and insulin were measured from saliva and serum samples. We conducted mixed-effect multiple linear regression modeling to account for the school variable as a random effect to assess the relationship between the sleep variables and salivary and serum biomarkers. Mediation analysis was conducted to check if BMI was a mediator between bedtime and the biomarkers.

**Results:**

There was a statistically significant elevation in serum IL-6 level associated with later bedtime (0.05 pg./mL, *p* = 0.01). Adolescents with severe sleep debt of ≥2 h had an increase in salivary IL-6 biomarker levels (0.38 pg./mL, *p* = 0.01) compared to those who had sleep debt of <1 h. Adolescents with sleep debt of ≥2 h had significantly higher levels of serum CRP (0.61 μg/mL, *p* = 0.02) than those without sleep debt. Additionally, we found that the inflammatory biomarkers (CRP, IL-6, IL-8, IL-10, VEGF, and MCP-1) and metabolic biomarkers (adiponectin, leptin, and insulin) had more statistically significant associations with the bedtime variables than with sleep duration variables. CRP, IL-6, and IL-8 were associated with sleep debt, and IL-6, VEGF, adiponectin, and leptin levels were associated with social jetlag. BMIz was a full mediator in the relationship between late bedtime and increased serum levels of CRP, IL-6, and insulin.

**Conclusion:**

Adolescents who go to bed at or later than midnight had dysregulated levels of salivary and serum inflammatory biomarkers, suggesting that disrupted circadian rhythm can trigger higher levels of systemic inflammation and potentially exacerbate chronic inflammation and the risk of metabolic diseases.

## Introduction

Sleep and lack of sleep have been reported to play a role in immune processes, such as cellular immune activation and nocturnal and functional secretions of inflammatory biomarkers (1). An inflammatory reaction precedes the development of the metabolic diseases, and certain inflammatory biomarkers can be targeted to prevent the development of inflammatory conditions and organ damage (2). Since biomarker levels are critical in the development of persistent inflammation in certain conditions, it is vital to explore their application as indicators of disease risks and severities and as prognostic markers. There is established evidence confirming a causal relationship between disrupted sleep, circadian rhythm, and the promotion of inflammatory conditions (3). Evaluating the correlation between inflammatory biomarkers and various sleep measurements could help identify which sleep behaviors to target to improve and regulate circadian rhythm, thereby preventing inflammation. The circadian rhythm is significantly influenced by the light–dark cycle and bedtime.

Numerous studies have investigated the relationship between various sleep parameters and inflammatory biomarkers, yielding inconsistent results (4–9). However, a meta-analysis of 72 studies with a combined sample size exceeding 50,000 participants has provided more consistent findings. This analysis revealed that sleep disturbance was associated with higher levels of C-reactive protein (CRP) and interleukin-6 (IL-6), while shorter sleep duration was associated with elevated CRP levels but not IL-6 (7).While most studies have focused on adults, a limited number of investigations have been conducted in adolescents. Some of these studies found a consistent positive relationship between CRP and poor sleep behavior in adolescents, although the association with IL-6 was not always observed in adolescents. These studies attributed the lack of association with IL-6 to the potential masking effect of adjusting for body mass index (BMI) (10). Grandner et al. (4) have proposed several reasons for the conflicting results between sleep and both CRP and IL-6, including type 2 errors (lack of findings despite the presence of a true association). These reasons encompass inconsistent definitions of sleep behaviors, heterogeneity of sleep measurements, inadequate sample sizes, insufficient stratification, selection bias, and inadequate control for confounding and mediating factors (4). Notably, an intervention study on adolescents demonstrated that lower baseline IL-6 and CRP levels were associated with improved treatment responses for behavioral and physical health outcomes following intervention (11). To the best of our knowledge, no studies have investigated the relationship between inflammatory biomarkers in adolescents in the Middle East. Identifying these biomarkers in this our population can provide valuable insights into the development of inflammatory conditions during adolescence. Adolescence is a critical developmental period associated with both significant risks and opportunities for establishing healthy behaviors. The present study aims to validate previous findings regarding selected inflammatory markers associated with poor sleep behavior, with the potential to inform future interventions by identifying biomarkers to measure and sleep behaviors to target.

This study aimed to evaluate the relationship between nine salivary and serum biomarkers such as interleukin (IL)-6, IL-8, IL-10, leptin, C-reactive protein (CRP), insulin, vascular endothelial growth factor (VEGF), and monocyte chemoattractant protein-1 (MCP-1), and different validated sleep parameters such as sleep duration, bedtime, sleep debt, and social jetlag in Kuwaiti adolescents. These biomarkers are implicated in multiple chronic conditions associated with inflammation, such as metabolic syndrome, rheumatoid arthritis, coronary artery disease, inflammatory bowel disease, and cancer (12–14).

This study was a part of a larger cohort study that aimed to evaluate the relationship between salivary biomarkers and hyperglycemia and obesity in adolescents (15). Therefore, the selection of biomarkers was based on their known association with diabetes and other inflammatory conditions.

Furthermore, it is important to acknowledge that the composition of human saliva is influenced by both local and systemic factors. Therefore, we collected saliva samples, taking into account the potential association between sleep and local inflammation (16).

As we investigate the link between sleep and inflammatory biomarkers, we cannot overlook the importance of bodyweight measurements, such as BMI. A wealth of evidence has demonstrated that insufficient sleep is associated with an increased BMI, and higher BMI is linked to inflammatory conditions (17). In this study, we also aimed to evaluate the mediation effect of BMI in the relationship between inflammatory biomarkers and bedtime.

## Materials and methods

### Study design and population

In March and April 2019, we collected data from 352 adolescents who were 16–19 years old and enrolled in Kuwait’s public high schools. The participants for this cross-sectional multi-site study were drawn from Kuwait’s Capital and Hawali governorates.

This study was part of a larger cohort study that aimed to evaluate the relationship between salivary biomarkers and hyperglycemia and obesity in adolescents (15).

The inclusion criteria for this study were senior male and female students enrolled in public high schools in the state of Kuwait who provided their consent to participate. A convenient sampling method was employed to select participants, and a total of 352 children were included in the study based on the allocated budget. The convenience sampling approach was adopted as it depended on the willingness of schools to host the research team for data collection.

In this study, students with medical conditions were not excluded; however, adjustments were made for these conditions during the analysis.

Responses were collected from students using the Qualtrics program (River Park Drive, Provo, UT 84604, United States), on individual iPads (Apple, Cupertino, CA).

This study was conducted during the academic year, and as such, the sleep behavior responses reflected the patterns of the school year. Additionally, data collection took place over a 2-month period, meaning there was no seasonal variation that could have impacted sleep timing.

## Ethical approval

This study was approved by the Dasman Diabetes Institute Human Ethical Review Committee in Kuwait and the Ministry of Health in Kuwait. We obtained informed consent from the parents or legal guardians and assent form from the adolescents participating in the study at the outset.

All procedures performed in studies involving human participants were as per the institutional and/or national research committee’s ethical standards.

### Salivary and serum biomarker levels

Salivary and serum biomarker levels were the primary dependent continuous variables in this study. Saliva samples were obtained from each participant during the academic year at schools early in the morning before breakfast, after an overnight fast (≥12 h). Salivary collection methodology has been described in an earlier study (18), where subjects were instructed to rinse their mouth with water and to provide 4 mL of an unstimulated whole saliva sample into a conical tube placed in a cup of crushed ice. The samples were processed and centrifuged at 2800 rpm for 20 min at 4°C at the Dasman Biobank and Biochemistry and Molecular Biology Department, Dasman Diabetes Institute Laboratories, Kuwait. Then, supernatants were transferred to a screwcap 2D barcoded storage tube (Thermo Scientific), read using a barcode reader (Thermo Scientific VisionMate™ ST), and frozen at −80°C. The analyzes were conducted at the Forsyth Institute in Cambridge, MA, United States.

Serum samples were collected in 7.5 mL of red and black marbled topped tubes with clot activator (SST tubes) from 333 subjects. After collection, the samples were centrifuged at 3000 rpm for 15 min at room temperature, then pipetted into the Eppendorf tubes and stored at −80°C. The frozen samples were transported to the Forsyth Institute under temperature-monitored dry ice. The samples were thawed at 4°C overnight and kept on ice throughout the assay procedure. Biomarker levels were measured in 25 μL of saliva and serum supernatants using multiplex magnetic bead panels on a Luminex 200™ system (Luminex, Austin, TX). Results were evaluated using Bio-Plex Manager™ (Version 5.0; Bio-Rad, Hercules, CA).

### Explanatory variables

The study was conducted during the academic year, so that the sleep timing responses could reflect the school year patterns.

#### Sleep duration weekdays and weekends

SDWday was calculated by counting the number of hours from the bedtime to the time of waking up on weekdays. The two validated questions (19) used were as follows: “During the past week, at what time did you go to bed on weekdays?” and “During the past week, at what time did you wake up on weekdays?” Data included continuous variables. SDWend was also calculated using the same abovementioned validated questions but for weekends.

#### Average sleep duration on weekdays and weekends

This variable was calculated by measuring the average sleep hours per week using the following formula: [(sleep duration weekday × 5) + (sleep duration weekend × 2)]/7 (20). This created a continuous variable.

#### Bedtime weekdays and weekends

A bedtime variable was used to evaluate the students’ sleep behavior using the question: “During the past week, at what time did you go to bed on weekdays/weekends?” This created a continuous variable representing bedtime in hours. We used the 24-h clock to record bedtimes (e.g., we coded a bedtime of 1:00 AM as 13:00 and 2:00 AM as 14:00), so that larger values indicated later bedtimes. We categorized bedtime into the following categories: before midnight (<12:00 AM), (from 12:00 to 1:00 AM), and (>1:00 AM).

#### Bedtime average weekdays and weekends

The BTAverage was calculated using the following formula: [(bedtime weekday × 5) + (bedtime weekend × 2)]/7, creating a continuous variable (21). We further investigated BTAverage as a categorical variable and categorized it into the following: (<12:00 AM), (from 12:00 to 1:00 AM), and (>1:00 AM).

Social jetlag was calculated using the following formula: SJL = bedtime weekends – bedtime weekdays (21). We created a continuous variable in hours, which was dichotomized based on the median as our cut-off value to classify the participants into two groups, with either absence (<1.5 h of SJL) or presence (≥1.5 h) of SJL (22).

Sleep debt was calculated using the formula: SD = SDWend − SDWday. And it was defined as a difference of ≥60 min (23). Severe sleep debt is a difference of ≥90 min (24), but in our study we counted severe sleep debt as >120 min as we had the duration variables in hours and not in minutes.

*Nap was* a binary variable that captured the habit of taking a nap in the afternoon. The study population was dichotomized into those who napped and those who did not.

#### BMI and obesity

Obesity was also analyzed as an independent binary variable, and participants were classified as obese or non-obese adolescents according to the WHO criteria (25). A stadiometer was used to measure height, and a calibrated digital scale was used to measure weight. BMI-for-sex/age *z*-score (BMIz) was calculated using WHO guidelines (25). Obese subjects had a *z*-score of ≥2 standard deviations (SD) units from the mean. Overweight subjects had 2 > *z*-scores ≥1 SD unit above the mean. Normal healthy weight was defined as values of ±1 SD unit. Underweight individuals were defined as those weighing ≤1 SD unit below the mean (25).

Data on age, sex, medical history, dietary habits, and screen time were self-reported. Screen-time was divided into adolescents who engaged in screen-based activities ≤2 h per day vs. those >2 h a day, and it includes the use of computers or smart devices and video games.

### Statistical analysis

A one-sample Kolmogorov–Smirnov test was conducted to test data normality. We transformed the skewed variables using the log 10 function. We conducted mixed-effect linear regression analyzes to adjust for school variable as a random effect and test the association of nine salivary and serum immunometabolic biomarkers as continuous outcome variables with all the sleep timing variables (SDWday, SDWend, SDAverge, BTWday, BTWend, BTAverage, SJL and SD), after adjusting for age, sex, dietary habits, screen time, napping, and medical history. Sex, age, and medical history are essential demographic variables that may influence the relationship between our measured outcomes and exposures. Additionally, nap, screen time, and dietary habits are potential confounding variables that could be associated with the outcomes and exposures, further elucidating their relationship.

To examine the mechanisms by which bedtime behavior influences biomarkers levels, we investigated BMI as a mediator in the associations between BTAverage and the nine salivary and serum biomarkers. Mediation analysis was conducted using structural equation modeling (SEM) (26). Analyzes included the 5,000-bootstrap method of resampling to estimate the confidence intervals for total, direct, and indirect effects. Goodness-of-fit tests were also conducted. Models were tested for multicollinearity. Models were tested for multicollinearity issues and exhibited Variance Inflation Factor (VIF) values of less than 1.6, supporting the absence of multicollinearity among parameters used as predictors. Statistical analyzes were performed on Stata/SE 15.1 (StataCorp, College Station, TX) statistical software. Results for the regression were reported using beta coefficient (β), and their 95% confidence intervals, with 0.05 significance level.

## Results

Table 1 shows the participants’ frequency and percent for BTWday, BTWend, and BTAverage, Table 2 presents their characteristics both stratified by average bedtime. Table 3 shows the biomarkers levels stratified by bedtime variables. Table 4 presents the mixed effect linear analysis examining the association between sleep timing variables (sleep duration and bedtime) with salivary and serum immunometabolic biomarkers, adjusting for age, sex, medical history, screen time, napping, and dietary habits. The mean age of adolescents was 17 ± 0.59 years. We found that for every one-hour reduction in SDWDay and SDAverage, the levels of salivary and serum IL-6 increased. There was a statistically significant association between salivary IL-6 level and SDWday (*p* = 0.02) and between serum IL-6 level and SDWday (*p* = 0.04) and SDAverage (*p* = 0.02).

**Table 1:** Descriptive summary of participants frequency and percentage stratified by bedtime as a categorical variable.

Variable	Frequency	Percent	Cumulative percent
Bedtime on Weekdays (BTWday)			
Before 12:00 AM	128	36.36	36.36
12:00–1:00 AM	162	46.02	82.39
After 1:00 AM	62	17.61	100
Total	352	100	
Bedtime on weekends (BTWend)			
Before 12:00 AM	29	8.24	8.24
12:00–1:00 AM	132	37.5	45.74
After 1:00 AM	191	54.26	100
Total	352	100	
Bedtime average (BTAverage)			
Before 12:00 AM	129	36.65	36.65
12:00–1:00 AM	98	27.84	64.49
After 1:00 AM	125	35.51	100
Total	352	100	

**Table 2:** Descriptive summary of population characteristics stratified by average bedtime on weekdays and weekends.

Independent variable	Bedtime average weekdays and weekends			Total	Value of p*
	< 12:00 AM	12:00–1:00 AM	> 1:00 AM		
	*n* = 129	*n* = 98	*n* = 125	*n* = 352	
Sex, *n* (%)					
Males	61 (47.29%)	45 (45.92%)	66 (52.80%)	172 (48.86%)	0.537
Females	68 (52.71%)	53 (54.08%)	59 (47.20%)	180 (51.14%)	
BMI, *n* (%)					
Underweight	5 (3.91%)	1 (1.02%)	1 (0.81%)	7 (2.00%)	0.051**
Normal Weight	61 (47.66%)	38 (38.78%)	51 (41.13%)	150 (42.86%)	
Overweight	28 (21.88%)	25 (25.51%)	19 (15.32%)	72 (20.57%)	
Obese	34 (26.56%)	34 (34.69%)	53 (42.74%)	121 (34.57%)	
Obesity, *n* (%)					
Non-Obese	95 (73.64%)	64 (65.31%)	72 (57.60%)	231 (65.62%)	0.027
Obese	34 (26.36%)	34 (34.69%)	53 (42.40%)	121 (34.38%)	
Screen time					
≤ 2 Hours/Day	21 (16.28%)	7 (7.14%)	9 (7.20%)	37 (10.51%)	0.027
> 2 Hours/Day	108 (83.72%)	91 (92.86%)	116 (92.80%)	315 (89.49%)	
Medical history, *n* (%)					
No health problem	104 (81.25%)	73 (74.49%)	92 (74.19%)	269 (76.86%)	0.718**
Diabetes	2 (1.56%)	3 (3.06%)	2 (1.61%)	7 (2.00%)	
Cardiovascular problems	0 (0%)	1 (1.02%)	1 (0.81%)	2 (0.57%)	
Others ***	22 (17.19%)	21 (21.43%)	29 (23.39%)	72 (20.57%)	
Taking Naps, *n* (%)					
No	61 (47.29%)	20 (20.41%)	26 (20.80%)	107 (30.40%)	<0.001
Yes	68 (52.71%)	78 (79.59%)	99 (79.20%)	245 (69.60%)	
Snoring, *n* (%)					
No	86 (66.67%)	61 (62.24%)	81 (64.80%)	228 (64.77%)	0.788
Yes	43 (33.33%)	37 (37.76%)	44 (35.20%)	124 (35.23%)	
Interrupted sleep, *n* (%)					
No	50 (38.76%)	48 (48.98%)	48 (38.40%)	146 (41.48%)	0.207
Yes	79 (61.24%)	50 (51.02%)	77 (61.60%)	206 (58.52%)	
Difficulty waking-up in the morning, *n* (%)					
No	31 (24.03%)	19 (19.39%)	19 (15.20%)	69 (19.60%)	0.207
Yes	98 (75.97%)	79 (80.61%)	106 (84.80%)	283 (80.40%)	
Difficulty falling asleep, *n* (%)					
No	99 (77.34%)	65 (66.33%)	88 (70.97%)	252 (72.00%)	0.179
Yes	29 (22.66%)	33 (33.67%)	36 (29.03%)	98 (28.00%)	
Feeling sleepy during the day, *n* (%)					
No	20 (15.50%)	2 (2.04%)	7 (5.60%)	29 (8.24%)	0.001**
Yes	109 (84.50%)	96 (97.96%)	118 (94.40%)	323 (91.76%)	
Waking-up during night sleep, *n* (%)					
No	70 (55.12%)	55 (56.12%)	67 (54.03%)	192 (55.01%)	0.952
Yes	57 (44.88%)	43 (43.88%)	57 (45.97%)	157 (44.99%)	
Sleep debt > 1 h, *n* (%)					
≤ 1 h or absent sleep debt	29 (22.48%)	5 (5.10%)	3 (2.40%)	37 (10.51%)	<0.001**
> 1 h	100 (77.52%)	93 (94.90%)	122 (97.60%)	315 (89.49%)	
Severe sleep debt > 2 h, *n* (%)					
≤ 2 h or absent severe sleep debt	52 (40.31%)	12 (12.24%)	6 (4.80%)	70 (19.89%)	<0.001
> 2 severe sleep debt	77 (59.69%)	86 (87.76%)	119 (95.20%)	282 (80.11%)	
Social jetlag, *n* (%)					
< 1.5 h or absent SJL	43 (33.33%)	48 (48.98%)	85 (68.00%)	176 (50.00%)	<0.001
≥ 1.5 h	86 (66.67%)	50 (51.02%)	40 (32.00%)	176 (50.00%)	

* = *p*-value < 0.05 calculated by chi-square test except when one or more of the cells counts is less than 5.

** = *p*-value < 0.05 calculated by Fisher’s exact test.

*** =Others medical conditions includes: anxiety, *n* = 15(4.2%); depression, *n* = 7(2%); asthma, *n* = 21(6%); irritable bowel syndrome, *n* = 5(1.4%); migraine, *n* = 5(1.4%); anemia, *n* = 3(0.8%); sickle cell anemia, *n* = 3(0.8%); kidney stone, *n* = 2(0.5%); stomach problem, *n* = 3(0.8%); seizure *n* = 1(0.3%); bone marrow transplant, *n* = 1(0.3%); whooping cough, *n* = 1(0.3%); Glucose-6-phosphate dehydrogenase (G6PD), *n* = 1(0.3%); polycystic ovary syndrome *n* = 1(0.3%); bleeding nose, *n* = 1(0.3%).

**Table 3:** Descriptive summary of population characteristics, biomarker levels, and sleep variables stratified by average bedtime (before 12:00 AM, 12:00–1:00 AM and, After 1:00 AM).

Variable	N	Mean	SD*	AVERAGE Bedtime								
				Bedtime before 12:00 AM *n* = 129			Bedtime 12:00–1:00 AM *n* = 84			Bedtime after 1:00 AM *n* = 139		
				*N*	Mean	SD*	*N*	Mean	SD*	*N*	Mean	SD*
Age	352	17.04	0.59	129	16.97	0.59	98	16.98	0.55	125	17.16	0.60
BMI	351	26.99	7.59	129	25.96	7.78	98	27.28	6.32	124	27.82	8.22
Waist circumference	352	77.13	25.37	129	76.31	24.36	98	77.67	24.06	125	77.56	27.48
Biomarkers												
CRP saliva	301	851.93	1568.48	104	988.19	2060.23	86	893.17	1478.28	111	692.29	1000.87
HSCRPugml	332	2.79	3.23	123	2.55	3.11	91	2.77	3.11	118	3.07	3.46
IL-6 saliva	281	10.48	34.55	107	7.20	18.02	74	18.27	61.97	99	8.25	11.63
IL-6 serum	224	1.20	0.72	85	1.10	0.75	67	1.27	0.72	71	1.26	0.68
IL-8 saliva	311	663.24	804.09	109	736.62	1023.99	89	662.64	732.74	112	591.28	590.95
IL-8 serum	309	7.18	12.28	116	8.20	15.74	85	7.32	14.02	107	5.99	3.34
IL-10 saliva	155	0.52	0.29	58	0.47	0.27	42	0.55	0.30	55	0.55	0.29
IL-10 serum	23	1.10	1.43	6	1.32	1.55	6	0.77	0.54	11	1.17	1.75
Adiponectin saliva	339	8163.36	11342.30	121	8350.86	15072.98	98	7907.07	9060.31	120	8183.59	8324.39
Adiponectin serum	330	9.89×10^+6^	8.71×10^+6^	122	1.01×10^+7^	7.18×10^+6^	90	1.06×10 ^+7^	1.26×10^+7^	117	8.98×10^+6^	5.94×10^+6^
Leptin saliva	282	202.10	130.56	107	210.09	132.71	77	200.48	137.35	97	196.09	123.21
Leptin serum	331	23872.53	23786.19	122	21102.62	24441.83	90	26001.80	22379.69	118	25273.19	24092.23
VEGF saliva	334	705.61	341.04	121	700.62	325.57	95	688.53	358.68	117	727.75	343.61
VEGF serum	332	87.08	76.04	123	84.02	69.56	90	81.75	71.89	118	94.72	85.28
Insulin saliva	346	397.92	358.09	126	380.43	330.98	97	392.29	378.66	122	420.55	371.25
Insulin serum	331	1005.96	942.64	122	902.79	792.72	90	1114.63	1158.65	118	1021.03	897.60
MCP1 saliva	347	390.32	278.81	125	400.59	285.40	97	370.55	252.15	124	397.37	293.25
MCP1 serum	332	312.78	165.29	123	298.17	158.89	90	300.58	156.08	118	336.20	177.20
Sleep variables												
Average sleep duration	352	7.04	1.20	129	8.01	0.80	98	7.15	0.78	125	5.95	0.88
Sleep duration weekdays	352	6.12	1.46	129	7.47	0.74	98	6.17	0.76	125	4.67	1.00
Sleep duration weekends	352	9.35	1.83	129	9.36	2.09	98	9.60	1.67	125	9.14	1.65
Sleep debt	352	3.24	2.25	129	1.89	2.21	98	3.43	1.72	125	4.47	1.88
Average bedtime	352	12.49	1.33	129	11.07	0.66	98	12.56	0.28	125	13.90	0.61
Bedtime weekdays	352	12.08	1.52	129	10.50	0.74	98	12.17	0.45	125	13.65	0.83
Bedtime weekends	352	13.51	1.41	129	12.52	1.28	98	13.52	0.97	125	14.54	1.06
Social jetlag	352	−1.43	1.48	129	2.02	1.43	98	1.35	1.29	125	0.89	1.45
Napping	236	2.72	1.12	66	2.12	0.82	76	2.78	1.01	94	3.10	1.20

**Table 4:** Mixed effect linear regression analysis for the association between sleep timing variables (sleep duration and bedtime) and salivary and serum immunometabolic biomarkers.

Biomarker	Independent Variable	Saliva					Serum				
		*β*	SE	*p*-value	95% CI		*β*	SE	*p*-value	95% CI	
CRP	Average sleep duration	0.01	0.03	0.64	−0.04	0.07	0.02	0.07	0.81	−0.12	0.15
	Sleep duration weekday	−0.004	0.02	0.88	−0.05	0.04	−0.04	0.06	0.48	−0.15	0.07
	Sleep duration weekend	0.02	0.02	0.18	−0.01	0.06	0.08	0.04	0.07	−0.01	0.16
	Average bedtime	−0.01	0.03	0.59	−0.07	0.04	0.03	0.06	0.69	−0.10	0.15
	Weekday bedtime	−0.01	0.02	0.72	−0.06	0.04	0.02	0.06	0.75	−0.09	0.13
	Weekend bedtime	−0.02	0.03	0.43	−0.07	0.03	0.03	0.06	0.65	−0.09	0.15
IL-6	Average sleep duration	−0.08	0.05	0.11	−0.17	0.02	−0.05	0.02	0.02*	−0.09	−0.01
	Sleep duration weekday	−0.10	0.04	0.02*	−0.17	−0.02	−0.04	0.02	0.04*	−0.07	−0.002
	Sleep duration weekend	0.03	0.03	0.26	−0.03	0.09	−0.02	0.01	0.21	−0.04	0.01
	Average bedtime	0.03	0.05	0.48	−0.06	0.12	0.05	0.02	0.01*	0.01	0.09
	Weekday bedtime	0.07	0.04	0.09	−0.01	0.14	0.04	0.02	0.04*	0.002	0.07
	Weekend bedtime	−0.11	0.04	0.02*	−0.19	−0.02	0.06	0.02	0.003*	0.02	0.09
IL-8	Average sleep duration	0.002	0.02	0.91	−0.03	0.03	0.01	0.01	0.23	−0.01	0.04
	Sleep duration weekday	−0.004	0.01	0.76	−0.03	0.02	0.02	0.01	0.08	−0.002	0.03
	Sleep duration weekend	0.01	0.01	0.39	−0.01	0.03	−0.004	0.01	0.52	−0.02	0.01
	Average bedtime	−0.01	0.01	0.68	−0.04	0.02	−0.03	0.01	0.01*	−0.05	−0.01
	Weekday bedtime	−0.001	0.01	0.94	−0.03	0.02	−0.02	0.01	0.01*	−0.04	−0.01
	Weekend bedtime	−0.02	0.01	0.23	−0.05	0.01	−0.01	0.01	0.18	−0.03	0.01
IL-10	Average sleep duration	−0.02	0.02	0.49	−0.06	0.03	−0.21	0.12	0.08	−0.43	0.02
	Sleep duration weekday	−0.02	0.02	0.31	−0.06	0.02	−0.10	0.09	0.27	−0.26	0.07
	Sleep duration weekend	0.004	0.01	0.75	−0.02	0.03	−0.27	0.12	0.02*	−0.50	−0.04
	Average bedtime	0.02	0.02	0.29	−0.02	0.07	0.11	0.11	0.33	−0.11	0.32
	Weekday bedtime	0.02	0.02	0.30	−0.02	0.06	0.09	0.10	0.35	−0.10	0.29
	Weekend bedtime	0.02	0.02	0.40	−0.02	0.06	0.08	0.11	0.44	−0.13	0.30
VEGF	Average sleep duration	−25.88	16.36	0.11	−57.94	6.18	−0.01	0.02	0.34	−0.04	0.02
	Sleep duration weekday	−25.52	13.60	0.06	−52.17	1.12	−0.02	0.01	0.19	−0.04	0.01
	Sleep duration weekend	0.61	10.28	0.95	−19.55	20.77	0.003	0.01	0.73	−0.02	0.02
	Average bedtime	15.18	15.26	0.32	−14.74	45.09	0.02	0.01	0.28	−0.01	0.04
	Weekday bedtime	18.79	13.24	0.16	−7.16	44.74	0.01	0.01	0.28	−0.01	0.04
	Weekend bedtime	−8.34	14.65	0.57	−37.06	20.38	0.01	0.01	0.54	−0.02	0.03
MCP-1	Average sleep duration	−0.001	0.02	0.93	−0.03	0.03	−0.01	0.01	0.48	−0.03	0.01
	Sleep duration weekday	−0.004	0.01	0.78	−0.03	0.02	−0.01	0.01	0.31	−0.02	0.01
	Sleep duration weekend	0.003	0.01	0.74	−0.02	0.02	0.002	0.01	0.73	−0.01	0.01
	Average bedtime	0.01	0.01	0.65	−0.02	0.03	0.02	0.01	0.09	0.00	0.03
	Weekday bedtime	0.01	0.01	0.59	−0.02	0.03	0.01	0.01	0.19	−0.01	0.03
	Weekend bedtime	0.0004	0.01	0.98	−0.03	0.03	0.02	0.01	0.04	0.00	0.03
Adiponectin	Average sleep duration	−0.02	0.02	0.27	−0.05	0.01	−0.001	0.01	0.93	−0.03	0.02
	Sleep duration weekday	−0.02	0.01	0.11	−0.05	0.01	0.002	0.01	0.84	−0.02	0.02
	Sleep duration weekend	0.01	0.01	0.56	−0.02	0.03	−0.005	0.01	0.56	−0.02	0.01
	Average bedtime	0.01	0.02	0.62	−0.02	0.04	−0.01	0.01	0.28	−0.04	0.01
	Weekday bedtime	0.01	0.01	0.30	−0.01	0.04	−0.01	0.01	0.45	−0.03	0.01
	Weekend bedtime	−0.02	0.02	0.23	−0.05	0.01	−0.02	0.01	0.14	−0.04	0.01
Leptin	Average sleep duration	0.01	0.01	0.39	−0.01	0.04	−0.02	0.02	0.48	−0.06	0.03
	Sleep duration weekday	0.005	0.01	0.63	−0.02	0.02	−0.03	0.02	0.12	−0.06	0.01
	Sleep duration weekend	0.01	0.01	0.30	−0.01	0.03	0.02	0.01	0.20	−0.01	0.04
	Average bedtime	−0.01	0.01	0.64	−0.03	0.02	0.03	0.02	0.08	0.00	0.07
	Weekday bedtime	−0.002	0.01	0.85	−0.02	0.02	0.03	0.02	0.10	−0.01	0.06
	Weekend bedtime	−0.01	0.01	0.30	−0.03	0.01	0.02	0.02	0.18	−0.01	0.06
Insulin	Average sleep duration	−0.01	0.02	0.62	−0.04	0.02	−0.01	0.01	0.42	−0.04	0.02
	Sleep duration weekday	−0.02	0.01	0.12	−0.05	0.01	−0.01	0.01	0.35	−0.03	0.01
	Sleep duration weekend	0.02	0.01	0.06	−0.001	0.04	−0.00001	0.01	1.00	−0.02	0.02
	Average bedtime	0.02	0.02	0.14	−0.01	0.05	0.01	0.01	0.30	−0.01	0.04
	Weekday bedtime	0.02	0.01	0.08	−0.003	0.05	0.01	0.01	0.27	−0.01	0.03
	Weekend bedtime	0.001	0.01	0.93	−0.03	0.03	0.01	0.01	0.64	−0.02	0.03

* = *p*-value < 0.05. *β*: beta coefficient. SE: Standard Error.

Adjusting for sex, age, medical history, nap, screen time and dietary habits.

Additionally, adjusting for confounders, we demonstrated that for every one-hour increase in BTWend (later bedtime), there was a statistically significant reduction in salivary IL-6 level (−0.11 pg./mL, *p* = 0.02). As for serum biomarkers, there was a statistically significant elevation in serum IL-6 level associated with BTAverage (0.05 pg./mL, *p* = 0.01), BTWday (0.04 pg./mL, *p* = 0.04), and BTWend (0.06 pg./mL, *p* = 0.003).

Table 5 shows the association of biomarkers with categorical average bedtime on weekdays and weekends (BTAverage). There was a significant elevation in salivary IL-6 level (0.44 pg./mL, *p* = 0.002) among participants who went to bed ‘from 12:00 to 1:00 AM’ compared to those who went to bed earlier, ‘before 12:00 AM’.

**Table 5:** Mixed effect linear analysis for the relationship between salivary and serum immunometabolic biomarkers and bedtime for average bedtime as categorical variable.

Biomarker	Independent variable	Saliva					Serum				
		*β*	SE	*p*-value	95% CI		*β*	SE	*p*-value	95% CI	
CRP	Average bedtime (Categorical)										
	12:00–1:00 AM (*vs* before 12:00 AM)	*0.07	0.09	0.40	−0.10	0.24	*0.22	0.20	0.27	−0.17	0.62
	After 1:00 AM (*vs* before 12:00 AM)	*-0.03	0.08	0.75	−0.19	0.14	*0.32	0.20	0.11	−0.07	0.71
IL-6	Average Bedtime (Categorical)										
	12:00–1:00 AM (*vs* before 12:00 AM)	*0.44	0.14	0.002	0.16	0.71	*0.14	0.06	0.02	0.02	0.26
	After 1:00 AM (*vs* before 12:00 AM)	*0.17	0.14	0.21	−0.10	0.44	*0.17	0.06	0.01	0.04	0.29
IL-8	Average Bedtime (Categorical)										
	12:00–1:00 AM (*vs* before 12:00 AM)	*0.004	0.05	0.94	−0.09	0.10	*-0.07	0.03	0.03	−0.13	−0.01
	After 1:00 AM (*vs* before 12:00 AM)	*-0.02	0.05	0.60	−0.12	0.07	*-0.09	0.03	0.004	−0.15	−0.03
IL-10	Average Bedtime (Categorical)										
	12:00–1:00 AM (*vs* before 12:00 AM)	*0.09	0.06	0.12	−0.02	0.21	*0.53	0.42	0.21	−0.29	1.35
	After 1:00 AM (*vs* before 12:00 AM)	*0.11	0.06	0.07	−0.009	0.22	*0.75	0.38	0.04	0.003	1.49
VEGF	Average Bedtime (Categorical)										
	12:00–1:00 AM (*vs* before 12:00 AM)	*6.06	48.30	0.90	−88.61	100.73	*0.001	0.05	0.98	−0.09	0.09
	After 1:00 AM (*vs* before 12:00 AM)	*53.05	47.30	0.26	−39.66	145.76	*0.05	0.04	0.28	−0.04	0.14
MCP-1	Average Bedtime (Categorical)										
	12:00–1:00 AM (*vs* before 12:00 AM)	*-0.03	0.05	0.57	−0.11	0.06	*0.02	0.03	0.45	−0.04	0.08
	After 1:00 AM (*vs* before 12:00 AM)	*0.02	0.04	0.60	−0.06	0.11	*0.05	0.03	0.08	−0.01	0.11
Adiponectin	Average Bedtime (Categorical)										
	12:00–1:00 AM (*vs* before 12:00 AM)	*0.02	0.05	0.71	−0.08	0.12	*-0.02	0.04	0.55	−0.09	0.05
	After 1:00 AM (*vs* before 12:00 AM)	*0.01	0.05	0.92	−0.09	0.10	*-0.05	0.04	0.16	−0.12	0.02
Leptin	Average Bedtime (Categorical)										
	12:00–1:00 AM (*vs* before 12:00 AM)	*-0.02	0.04	0.65	−0.09	0.06	*0.15	0.06	0.02	0.03	0.27
	After 1:00 AM (*vs* before 12:00 AM)	*-0.01	0.04	0.69	−0.08	0.06	*0.14	0.06	0.02	0.02	0.26
Insulin	Average Bedtime (Categorical)										
	12:00–1:00 AM (*vs* before 12:00 AM)	*0.02	0.05	0.68	−0.08	0.12	*0.10	0.04	0.01	0.02	0.17
	After 1:00 AM (*vs* before 12:00 AM)	*0.09	0.05	0.08	−0.01	0.18	*0.08	0.04	0.04	0.004	0.16

Table 6 shows the association of biomarkers with other sleep variables (sleep debt and social jetlag). Adolescents with a sleep debt of ≥1 h and a severe sleep debt of ≥2 h had an increase in salivary IL-6 biomarker levels (0.35 pg./mL, *p* = 0.04; 0.38 pg./mL, *p* = 0.01), respectively, compared to those who had sleep debt of <1 h. Participants with a sleep debt of ≥1 h had significantly higher levels of serum IL-6 (0.17 pg./mL, *p* = 0.03) than those without sleep debt or those with sleep debt of <1 h. Students with sleep debt of ≥2 h had significantly higher levels of serum CRP (0.61 μg/mL, *p* = 0.02) than those without sleep debt or those with sleep debt of <2 h.

**Table 6:** Mixed effect linear regression analysis for the relationship between salivary and serum immunometabolic biomarkers and the sleep variables, social jetlag and sleep debt.

Biomarker	Independent Variable	Saliva					Serum				
		*β*	SE	*p*-value	95% CI		*β*	SE	*p*-value	95% CI	
CRP	Sleep Debt: ≥ 60 min (*vs* <60 min)	0.12	0.12	0.30	−0.11	0.36	0.61	0.26	0.02*	0.11	1.11
	Severe Sleep Debt: ≥ 120 min (*vs* <120 min)	−0.01	0.08	0.91	−0.18	0.16	0.13	0.20	0.52	−0.26	0.51
	Social Jetlag: continuous	−0.01	0.02	0.71	−0.06	0.04	0.01	0.06	0.92	−0.10	0.11
	Social Jetlag: ≥ 1.5 h (*vs* < 1.5 h)	−0.02	0.07	0.76	−0.15	0.11	0.002	0.16	0.99	−0.32	0.32
IL-6	Sleep Debt: ≥ 60 min (*vs* <60 min)	0.35	0.18	0.04*	0.005	0.70	0.17	0.08	0.03*	0.02	0.33
	Severe Sleep Debt: ≥ 120 min (*vs* <120 min)	0.38	0.14	0.01*	0.11	0.64	0.02	0.06	0.70	−0.09	0.14
	Social Jetlag: continuous	−0.14	0.04	<0.001	−0.22	−0.07	0.01	0.02	0.54	−0.03	0.05
	Social Jetlag: ≥ 1.5 h (*vs* < 1.5 h)	−0.47	0.11	<0.001	−0.69	−0.26	−0.08	0.05	0.12	−0.18	0.02
IL-8	Sleep Debt: ≥ 60 min (*vs* <60 min)	0.03	0.06	0.61	−0.09	0.15	−0.08	0.04	0.04	−0.17	−0.001
	Severe Sleep Debt: ≥ 120 min (*vs* <120 min)	0.04	0.05	0.43	−0.05	0.13	−0.05	0.03	0.11	−0.11	0.01
	Social Jetlag: continuous	−0.01	0.01	0.32	−0.04	0.01	0.01	0.01	0.19	−0.01	0.03
	Social Jetlag: ≥ 1.5 h (*vs* < 1.5 h)	−0.04	0.04	0.31	−0.11	0.04	0.01	0.03	0.70	−0.04	0.06
IL-10	Sleep Debt: ≥ 60 min (*vs* <60 min)	0.10	0.08	0.23	−0.06	0.26	0.07	0.35	0.85	−0.62	0.75
	Severe Sleep Debt: ≥ 120 min (*vs* <120 min)	0.12	0.07	0.08	−0.01	0.24	0.07	0.35	0.85	−0.62	0.75
	Social Jetlag: continuous	−0.01	0.02	0.76	−0.05	0.04	−0.01	0.02	0.76	−0.05	0.04
	Social Jetlag: ≥ 1.5 h (*vs* < 1.5 h)	−0.01	0.06	0.91	−0.11	0.10	0.33	0.38	0.39	−0.42	1.08
VEGF	Sleep Debt: ≥ 60 min (*vs* <60 min)	73.93	63.14	0.24	−49.82	197.69	0.05	0.06	0.36	−0.06	0.17
	Severe Sleep Debt: ≥ 120 min (*vs* <120 min)	48.83	47.44	0.30	−44.14	141.80	0.06	0.04	0.18	−0.03	0.15
	Social Jetlag: continuous	−26.99	13.54	0.04*	−53.53	−0.45	−0.01	0.01	0.61	−0.03	−0.03
	Social Jetlag: ≥ 1.5 h (*vs* < 1.5 h)	−50.28	38.20	0.19	−125.14	24.59	−0.04	0.04	0.26	−0.11	0.03
MCP-1	Sleep Debt: ≥ 60 min (*vs* <60 min)	0.06	0.06	0.31	−0.05	0.17	0.02	0.04	0.66	−0.06	0.09
	Severe Sleep Debt: ≥ 120 min (*vs* <120 min)	0.03	0.04	0.57	−0.06	0.11	0.02	0.03	0.44	−0.03	0.08
	Social Jetlag: continuous	−0.01	0.01	0.61	−0.03	0.02	0.004	0.01	0.59	−0.01	0.02
	Social Jetlag: ≥ 1.5 h (*vs* < 1.5 h)	0.001	0.04	0.98	−0.07	0.07	0.003	0.02	0.91	−0.04	0.05
Adiponectin	Sleep Debt: ≥ 60 min (*vs* <60 min)	0.06	0.07	0.41	−0.08	0.19	−0.04	0.05	0.44	−0.13	0.06
	Severe Sleep Debt: ≥ 120 min (*vs* <120 min)	0.08	0.05	0.13	−0.02	0.18	0.01	0.04	0.70	−0.06	0.08
	Social Jetlag: continuous	−0.03	0.01	0.03	−0.06	−0.003	−0.01	0.01	0.56	−0.03	0.01
	Social Jetlag: ≥ 1.5 h (*vs* < 1.5 h)	−0.07	0.04	0.11	−0.15	0.01	0.004	0.03	0.89	−0.05	0.06
Leptin	Sleep Debt: ≥ 60 min (*vs* <60 min)	0.01	0.05	0.82	−0.08	0.10	0.15	0.08	0.06	−0.003	0.31
	Severe Sleep Debt: ≥ 120 min (*vs* <120 min)	−0.02	0.04	0.65	−0.09	0.05	0.08	0.06	0.20	−0.04	0.21
	Social Jetlag: continuous	−0.01	0.01	0.46	−0.03	0.01	−0.01	0.02	0.70	−0.04	0.03
	Social Jetlag: ≥ 1.5 h (*vs* < 1.5 h)	−0.06	0.03	0.04*	−0.11	0.00	0.01	0.05	0.80	−0.09	0.11
Insulin	Sleep Debt: ≥ 60 min (*vs* <60 min)	0.09	0.06	0.17	−0.04	0.21	0.02	0.04	0.66	−0.06	0.09
	Severe Sleep Debt: ≥ 120 min (*vs* <120 min)	0.06	0.05	0.19	−0.03	0.16	0.02	0.03	0.44	−0.03	0.08
	Social Jetlag: continuous	−0.02	0.01	0.10	−0.05	0.004	0.004	0.01	0.59	−0.01	0.02
	Social Jetlag: ≥ 1.5 h (*vs* < 1.5 h)	−0.05	0.04	0.25	−0.12	0.03	0.003	0.02	0.91	−0.04	0.05

* = *p*-value < 0.05. SE: Standard Error. Adjusting for sex, age, medical history, nap, screen time, and dietary habits.

Only salivary biomarkers showed significant associations with SJL. For every one-hour increase in social jetlag, there was an overall reduction in the following salivary biomarkers IL-6 (−0.14 pg./mL, *p* < 0.001), adiponectin (−0.03 pg./mL, p = 0.03), and VEGF (−27 pg./mL, *p* = 0.04). We further investigated the effect of social jetlag with a median cut-off point of 1.5 h of social jetlag. Adolescents with a social jetlag of ≥1.5 h had a reduction in salivary IL-6 (−0.47 pg./mL, *p* < 0.001) and leptin (−0.06 pg./mL, *p* = 0.04) levels compared to that no social jetlag or those with a social jetlag of <1.5 h.

Table 7 shows the results of the components of the mediation analysis using the SEM technique. We showed consistent evidence of full mediation by BMI for serum CRP, IL-6, and insulin levels.

**Table 7:** Mediation effect of BMI on the association between serum and salivary biomarkers and average bedtime.

Biomarker	Characteristics	Estimate	SE	CI		*p*-value
Serum CRP	Direct effect	0.151	0.206	−0.253	0.554	0.464
	Indirect effect	0.277	0.132	0.018	0.536	0.036
	Total effect	0.428	0.245	−0.052	0.908	0.081
Serum IL-6	Direct effect	0.077	0.042	0.069	−0.006	0.160
	Indirect effect	0.021	0.009	0.018	0.004	0.039
	Total effect	0.098	0.042	0.018	0.017	0.180
Serum insulin	Direct effect	0.017	0.020	0.404	−0.023	0.056
	Indirect effect	0.022	0.011	0.047	0.0003	0.043
	Total effect	0.038	0.023	0.095	−0.007	0.083

The confidence intervals and standard errors were obtained using 5,000 bootstraps. Goodness of fit tests: comparative fit index = 1.00, Tucker–Lewis index = 1.00. Size of residuals: standardized root mean squared residual = 0.00.

## Discussion

We assessed multiple sleep parameters, including bedtime, sleep duration, sleep debt, and social jetlag. We observed that the levels of salivary IL-6 and serum IL-6, IL-10, MCP-1, and insulin increase with delayed bedtime. We also found that serum IL-6 level was associated with sleep debt, and that salivary IL-6 level is associated with SJL.

Sleep debt is defined as the difference in sleep duration between weekdays and weekends. Increased sleep debt have a regulatory effect in controlling low-grade systemic inflammation among adults with sleep deprivation on weekdays (27).

Adolescents who had longer weekend catch-up sleep (sleep debt) had higher levels of salivary IL-6 and serum CRP and IL-6. The relationship between increased sleep debt and higher levels of CRP could be explained by multiple factors. First, delayed night sleep can trigger inflammation in different body organs owing to hormonal disruption (28, 29), and night sleep deprivation suppresses immunity, as observed by increased levels of cytokine inflammatory markers (30). Second, the level of CRP is higher in children with obesity (31), and chronic inflammation is a characteristic of obesity, as adipocytes trigger metabolic signals that induce inflammation as indexed by increases in the levels of CRP and other inflammatory markers such as IL6 with disruption of sleep (32).

Furthermore, the evening chronotype (natural tendency to go to bed and wake up later) leads to the accumulation of sleep debt, which is later recovered on weekends, leading to circadian disruption during the week (33) and creating what is called ‘social jetlag’, the most frequent form of circadian rhythm misalignment. Recent studies have investigated social jetlag as a potential risk factor for the development of metabolic diseases (34, 35).

Social jetlag phenomenon occurs when an individual’s sleep and wake times during the workweek differ significantly from those on their days off or during weekend, causing a misalignment of the internal clock with the external environment. This can lead to symptoms similar to those of jet lag, such as fatigue, mood disturbances, and impaired cognitive performance, and potentially chronic inflammatory diseases.

Students with a social jetlag of ≥1.5 h had lower salivary and serum VEGF levels. Salivary VEGF is not an exudate from the bloodstream; in humans, it is secreted from the parotid glands (36–38). Several studies in adults showed that sleep restriction among adults is associated with a higher risk of oral inflammatory disease, such as periodontitis (39, 40). Two studies found that higher secretion of salivary VEGF is prevalent in healthy individuals, and that lower secretion is prevalent in those with periodontitis (41, 42). These results suggest the protective nature of salivary VEGF in maintaining healthy oral mucosa and periodontal tissues (41).

The increase in salivary IL-6 levels noted with multiple sleep parameters in our study may reflect localized oral inflammation (39, 43). Our earlier prospective longitudinal study of Kuwaiti children showed that inadequate sleep can induce hyperglycemia, leading to gingivitis, which may progress to periodontitis over time (40).

Further, our results showed an overall increase in serum insulin is associated with later bedtimes. Evidence shows that sleep deprivation triggers an increase in insulin resistance (44). Insulin resistance develops owing to high levels of insulin secretion, which leads to the development of type 2 diabetes. A strong body of evidence shows a positive association between insulin and sleep deprivation independent of age, sex, ethnicity, BMI, and physical activity (45).

Further examination of BMI in the mediation analysis showed that increased BMI was a full mediator in the relationship between increased CRP, IL-6, and insulin levels and bedtime. Although several studies have addressed the associations between higher levels of inflammatory cytokines and the risk of increased bodyweight, to our knowledge, this is the first study to examine the mediation effect of increased BMI in the relationship between sleep timing and systemic inflammation as indexed by inflammatory biomarkers. We showed that later bedtime was associated with IL6, IL8, insulin, CRP, and MCP-1, and short sleep duration was only associated with IL6. Being a full mediator, increased BMI is involved in the causal pathway that can trigger inflammation and subsequent comorbidities.

Studies show that disrupted circadian rhythms in adolescents increased the levels of inflammatory cytokines and can predict metabolic syndrome (46). Circadian disruption (47, 48) has received increasing research attention. Circadian rhythms are critical for the optimal function of the immune system, and their disruption can affect host immune responses and increase susceptibility and development of pathology (49). Circadian rhythms are regulated by the sleep–wake and dark–light cycles. At the molecular level, circadian rhythms are driven by the molecular circadian clock, which is found in nearly every cell in the body (50). Circadian rhythms are mainly disrupted by irregular sleep–wake schedules and other environmental factors, such as light exposure at night and eating food late at night, resulting in cellular and organ dysfunction, including metabolic pathology (50). Understanding the interaction between circadian rhythms manifested by poor sleep behavior and systemic inflammation could provide new insights for intervention to improve lifestyle and prevent consequences of metabolic syndrome.

This study had some limitations. Owing to the cross-sectional design, we were unable to infer a causal relationship. We did not utilize randomized sample recruitment in this study, which may leave it vulnerable to selection bias. Additionally, the data on sleep duration were self-reported; hence, information was susceptible to recall bias.

Another limitation of this study is that we did not collect data on insomnia and sleep-disordered breathing and local inflammation, which could have played roles in the levels of inflammatory salivary biomarkers.

## Conclusion

We observed that the levels of salivary IL-6 and serum IL-6, IL-10, MCP-1, and insulin increase with delayed bedtime. Adolescents who had longer weekend catch-up sleep had higher levels of salivary IL-6 and serum CRP and IL-6. These changes in biomarker levels with sleep timing behavior support the hypothesis that late bedtime and disrupted circadian rhythm promote local and systemic inflammation that can trigger chronic metabolic diseases.

In conclusion, while conventional wisdom has emphasized the role of diet and physical activity in preventing inflammatory and metabolic diseases, emerging studies suggest that improving sleep may also be a valuable approach. Although controlling diet and physical activity can be challenging, addressing sleep behaviors holds promise for mitigating inflammation and reducing the risk of metabolic diseases. The present study serves as a crucial preliminary investigation in identifying specific sleep behaviors to target and inflammatory biomarkers to measure, thereby offering potential strategies for improving health outcomes in this population. Further research in this area is warranted to develop comprehensive interventions that encompass sleep as a key component in disease prevention and management. By recognizing the importance of sleep in promoting health and preventing inflammation and metabolic diseases, healthcare providers and individuals alike can prioritize sleep as a valuable aspect of overall well-being.

## Data availability statement

The raw data supporting the conclusions of this article will be made available by the authors, without undue reservation.

## Ethics statement

This study was approved by the Dasman Diabetes Institute Human Ethical Review Committee in Kuwait and the Ministry of Health in Kuwait. We obtained informed consent from the parents or legal guardians and assent form from the adolescents participating in the study at the outset. All procedures performed in studies involving human participants were as per the institutional and/or national research committee’s ethical standards. Written informed consent to participate in this study was provided by the participants’ legal guardian/next of kin.

## Author contributions

HA and AA contributed to writing the original draft of the manuscript, data analysis, and data interpretation. SB-H contributed to the study methodology, data interpretation, and critical revision of the manuscript. FA-M, MA, SD, and JA contributed to the data collection, project administration, and they revised the manuscript. MT contributed to the conception of the study, study methodology, and critically revised the manuscript. NR contributed to the data analysis and data interpretation. MF contributed to the data analysis and critical revision of the manuscript. All authors provided final approval and agreed to be accountable for all aspects of the work.

## Funding

This study was funded by the Dasman Diabetes Institute, Kuwait (Grant # RA/065/2011 and RA/005/2011); the Kuwait Foundation for the Advancement of Sciences, Kuwait (Grant # PR19-13MM-01); and the Forsyth Institute, Cambridge, MA, United States (Grant # FSI-CP02). This work would not have been possible without the support of the Kuwait School Oral Health Program, Ministry of Health in Kuwait.

## Conflict of interest

The authors declare that the research was conducted in the absence of any commercial or financial relationships that could be construed as a potential conflict of interest.

## Publisher’s note

All claims expressed in this article are solely those of the authors and do not necessarily represent those of their affiliated organizations, or those of the publisher, the editors and the reviewers. Any product that may be evaluated in this article, or claim that may be made by its manufacturer, is not guaranteed or endorsed by the publisher.
