# Supplementary material for: The relationship between sleep and salivary and serum inflammatory biomarkers in adolescents
Source: Front Med (Lausanne). 2023 May 26;10:1175483. doi: 10.3389/fmed.2023.1175483 (PMC10250646; doi:10.3389/fmed.2023.1175483)
Supplement: Supplementary file 2 [file Data_Sheet_2.PDF]

```
**************************************************
***********Doctorate Thesis Do-File**************
**************************************************
*Importing the data
import excel "/Users/dshdoshdshdosh/Desktop/Doc
> torate thesis/Final data/Final data for analysi
> s/Copy of All_Merged_with food.xlsx", sheet("AL
> L_MERGED") firstrow clear


*Cleaning the data
drop IL10_P1
drop IL12p70_P1
drop IL13_P1
drop IL17A_P1
drop IL1b_P1
drop IL4_P1
drop IL8_P1
drop MCP1_P1
drop TNFa_P1
drop VEGF_P1
drop Ghrelin_P1
drop Insulin_P1
drop Leptin_P1
drop MPO_P1
drop MMP9_P1
drop CRP_P1
drop Resistin_P1
drop Leptin_P2
drop VEGF_P2
drop MCP1_P2
drop IL10_P2
drop IL8_P2
drop Insulin_P2
drop CRP_P2
drop IFNgamma_P1
drop Visit_dateP1
drop Visit_dateP2
drop Waist_cm1
drop Height_cm1
drop Weight_kg1
drop Height_cm2
drop Weight_kg2
drop AgeP1
drop AgeP2
drop BMI1
drop BMI2
drop WHOcat1
drop OBWHO1
drop WHOcat2
```

```
drop OBWHO2
drop WHOcatP1
drop WHOcatP2
drop Waist_cm2
**********************************************************************
**********
*Categorization:
tab Sex
gen sex_n=.
replace sex_n = 1 if Sex == 1
replace sex_n = 2 if Sex == 2
label define Sex 1 "Male" 2 "Female"
label values sex_n Sex
tab sex_n

tab med_hist
gen med_hist_n=.
replace med_hist_n = 0 if med_hist == 0
replace med_hist_n = 1 if med_hist == 1
replace med_hist_n = 2 if med_hist == 2
replace med_hist_n = 3 if med_hist == 3
replace med_hist_n = 4 if med_hist == 4
replace med_hist_n = 5 if med_hist == 5
replace med_hist_n = 99 if med_hist == 99
label define med_hist 0 "No health problem" 1 "Diabetes" 2 "Heart
Disease" 3 "Anxiety" 4 "Depression" 5 "Asthma" 99 "Others"
label values med_hist_n med_hist
tab med_hist_n
tab medical_history
gen medical_history_n =.
replace medical_history_n = 0 if medical_history == 0
replace medical_history_n = 1 if medical_history == 1
replace medical_history_n = 2 if medical_history == 2
replace medical_history_n = 3 if medical_history == 3
label define medical_history 0 "No health problem" 1 "Diabetes" 2
"Heart disease" 3 "Others"
label value medical_history_n medical_history
tab medical_history_n

tab meds
gen meds_n=.
replace meds_n = 0 if meds == 0
replace meds_n = 1 if meds == 1
label define meds 0 "Not on medications" 1 " On medications"
label value meds_n meds
tab meds_n

tab mom_diab
gen mom_diab_n=.
replace mom_diab_n = 0 if mom_diab == 0
```

```
replace mom_diab_n = 1 if mom_diab == 1
replace mom_diab_n = 2 if mom_diab == 2
label define mom_diab 0 "Mother not diabetic" 1 "Mother diabetic" 2 "I
do not know"
label value mom_diab_n mom_diab
tab mom_diab_n

tab dad_diab
gen dad_diab_n=.
replace dad_diab_n = 0 if dad_diab == 0
replace dad_diab_n = 1 if dad_diab == 1
replace dad_diab_n = 2 if dad_diab == 2
label define dad_diab 0 "Father not diabetic" 1 "Father diabetic" 2 "I
do not know"
label value dad_diab_n dad_diab
tab dad_diab_n

tab mom_edu
gen mom_edu_n=.
replace mom_edu_n = 0 if mom_edu == 0
replace mom_edu_n = 1 if mom_edu == 1
replace mom_edu_n = 2 if mom_edu == 2
replace mom_edu_n = 3 if mom_edu == 3
replace mom_edu_n = 4 if mom_edu == 4
replace mom_edu_n = 5 if mom_edu == 5
replace mom_edu_n = 6 if mom_edu == 6
label define mom_edu 0 "No degree" 1 "Less than highschool" 2
"Highschool degree" 3 "Diplomate or certificate from institute" 4
"Bachelor degree" 5 "Master or Doctorate" 6 "I do not know"
label value mom_edu_n mom_edu
tab mom_edu_n
tab mother_ed
gen mother_ed_n=.
replace mother_ed_n = 0 if mom_edu_n == 0 | mom_edu_n == 1
replace mother_ed_n = 1 if mom_edu_n == 2 | mom_edu_n == 3
replace mother_ed_n = 2 if mom_edu_n == 4
replace mother_ed_n = 3 if mom_edu_n == 5
replace mother_ed_n = 4 if mom_edu_n == 6
label define mom_edu_n 0 "Less than highschool" 1 " Highschool or
Diploma" 2 "Bachelor degree" 3 "Masters or Doctorate" 4 "I do not
know"
label value mother_ed_n mom_edu_n
tab mother_ed_n

tab dad_edu
gen dad_edu_n=.
replace dad_edu_n = 0 if dad_edu == 0
replace dad_edu_n = 1 if dad_edu == 1
replace dad_edu_n = 2 if dad_edu == 2
replace dad_edu_n = 3 if dad_edu == 3
```

```
replace dad_edu_n = 4 if dad_edu == 4
replace dad_edu_n = 5 if dad_edu == 5
replace dad_edu_n = 6 if dad_edu == 6
label define dad_edu 0 "No degree" 1 "Less than highschool" 2
"Highschool degree" 3 "Diplomate or certificate from institute" 4
"Bachelor degree" 5 "Master or Doctorate" 6 "I do not know"
label value dad_edu_n dad_edu
tab dad_edu_n
tab fath_ed
gen fath_ed_n=.
replace fath_ed_n = 0 if dad_edu_n == 0 | dad_edu_n == 1
replace fath_ed_n = 1 if dad_edu_n == 2 | dad_edu_n == 3
replace fath_ed_n = 2 if dad_edu_n == 4
replace fath_ed_n = 3 if dad_edu_n == 5
replace fath_ed_n = 4 if dad_edu_n == 6
label define dad_edu_n 0 "Less than highschool" 1 " Highschool or
Diploma" 2 "Bachelor degree" 3 "Masters or Doctorate" 4 "I do not
know"
label value fath_ed_n dad_edu_n
tab fath_ed_n


tab sleep_weekday
gen sleep_weekday_n=.
replace sleep_weekday_n = 1 if sleep_weekday == 1
replace sleep_weekday_n = 2 if sleep_weekday == 2
replace sleep_weekday_n = 3 if sleep_weekday == 3
replace sleep_weekday_n = 21 if sleep_weekday == 21
replace sleep_weekday_n = 22 if sleep_weekday == 22
replace sleep_weekday_n = 23 if sleep_weekday == 23
replace sleep_weekday_n = 24 if sleep_weekday == 24
replace sleep_weekday_n = 99 if sleep_weekday == 99
label define sleep_weekday 1 "1:00 a.m" 2 "2:00 a.m" 3 "3:00 a.m" 21
"9:00 p.m" 22 "10:00 p.m" 23 "11:00 p.m" 24 "12:00 a.m" 99 "Other"
label values sleep_weekday_n sleep_weekday
tab sleep_weekday_n

tab wakeup_weekday
gen wakeup_weekday_n=.
replace wakeup_weekday_n = 5 if wakeup_weekday == 5
replace wakeup_weekday_n = 6 if wakeup_weekday == 6
replace wakeup_weekday_n = 7 if wakeup_weekday == 7
replace wakeup_weekday_n = 4 if wakeup_weekday == 4
label define wakeup_weekday 5 "5:00 a.m" 6 "6:00 a.m" 7 "7:00 a.m" 4
"4:00 a.m"
label value wakeup_weekday_n wakeup_weekday
tab wakeup_weekday_n

tab Sleep_time_weekday
gen Sleep_time_weekday_n=.
```

```
replace Sleep_time_weekday_n= 1 if Sleep_time_weekday == 1
replace Sleep_time_weekday_n= 2 if Sleep_time_weekday == 2
replace Sleep_time_weekday_n= 3 if Sleep_time_weekday == 3
replace Sleep_time_weekday_n= 4 if Sleep_time_weekday == 4
replace Sleep_time_weekday_n= 5 if Sleep_time_weekday == 5
replace Sleep_time_weekday_n= 6 if Sleep_time_weekday == 6
replace Sleep_time_weekday_n= 7 if Sleep_time_weekday == 7
replace Sleep_time_weekday_n= 8 if Sleep_time_weekday == 8
replace Sleep_time_weekday_n= 9 if Sleep_time_weekday == 9
label define Sleep_time_weekday 1 " 1hr/night" 2 "2hr/night" 3 "3hr/
night" 4 "4hr/night" 5 "5hr/night" 6 "6hr/night" 7 "7hr/night" 8 "8hr/
night" 9 "9hr/night"
label values Sleep_time_weekday_n Sleep_time_weekday
tab Sleep_time_weekday_n

tab sleep_weekend
gen sleep_weekend_n=.
replace sleep_weekend_n = 1 if sleep_weekend == 1
replace sleep_weekend_n = 2 if sleep_weekend == 2
replace sleep_weekend_n = 3 if sleep_weekend == 3
replace sleep_weekend_n = 4 if sleep_weekend == 4
replace sleep_weekend_n = 5 if sleep_weekend == 5
replace sleep_weekend_n = 6 if sleep_weekend == 6
replace sleep_weekend_n = 7 if sleep_weekend == 7
replace sleep_weekend_n = 8 if sleep_weekend == 8
replace sleep_weekend_n = 9 if sleep_weekend == 9
replace sleep_weekend_n = 21 if sleep_weekend == 21
replace sleep_weekend_n = 22 if sleep_weekend == 22
replace sleep_weekend_n = 23 if sleep_weekend == 23
replace sleep_weekend_n = 24 if sleep_weekend == 24
replace sleep_weekend_n = 99 if sleep_weekend == 99
label define sleep_weekend 1 "1:00 a.m" 2 "2:00 a.m" 3 "3:00 a.m" 4
"4:00 a.m" 5 "5:00 a.m" 6 "6:00 a.m" 7 "7:00 a.m" 8 "8:00 a.m" 9 "9:00
a.m" 21 "9:00 p.m" 22 "10:00 p.m" 23 "11:00 p.m" 24 "12:00 a.m" 99
"Other"
label values sleep_weekend_n sleep_weekend
tab sleep_weekend_n

tab wakeup_weekend
gen wakeup_weekend_n=.
replace wakeup_weekend_n = 6 if wakeup_weekend == 6
replace wakeup_weekend_n = 7 if wakeup_weekend == 7
replace wakeup_weekend_n = 8 if wakeup_weekend == 8
replace wakeup_weekend_n = 9 if wakeup_weekend == 9
replace wakeup_weekend_n = 10 if wakeup_weekend == 10
replace wakeup_weekend_n = 11 if wakeup_weekend == 11
replace wakeup_weekend_n = 12 if wakeup_weekend == 12
replace wakeup_weekend_n = 13 if wakeup_weekend == 13
replace wakeup_weekend_n = 14 if wakeup_weekend == 14
replace wakeup_weekend_n = 15 if wakeup_weekend == 15
```

```
replace wakeup_weekend_n = 16 if wakeup_weekend == 16
replace wakeup_weekend_n = 17 if wakeup_weekend == 17
replace wakeup_weekend_n = 18 if wakeup_weekend == 18
replace wakeup_weekend_n = 99 if wakeup_weekend == 99
label define wakeup_weekend 6 "6:00 a.m" 7 "7:00 a.m" 8 "8:00 a.m" 9
"9:00 a.m" 10 "10:00 a.m" 11 "11:00 a.m" 12 "12:00 p.m" 13 "1:00 p.m"
14 "2:00 p.m" 15 "3:00 p.m" 16 "4:00 p.m" 17 "5:00 p.m" 18 "6:00 p.m"
99 "Other"
label value wakeup_weekend_n wakeup_weekend
tab wakeup_weekend_n

tab Sleep_time_weekend
gen Sleep_time_weekend_n=.
replace Sleep_time_weekend_n= 1 if Sleep_time_weekend == 1
replace Sleep_time_weekend_n= 4 if Sleep_time_weekend == 4
replace Sleep_time_weekend_n= 5 if Sleep_time_weekend == 5
replace Sleep_time_weekend_n= 6 if Sleep_time_weekend == 6
replace Sleep_time_weekend_n= 7 if Sleep_time_weekend == 7
replace Sleep_time_weekend_n= 8 if Sleep_time_weekend == 8
replace Sleep_time_weekend_n= 9 if Sleep_time_weekend == 9
replace Sleep_time_weekend_n= 10 if Sleep_time_weekend == 10
replace Sleep_time_weekend_n= 11 if Sleep_time_weekend == 11
replace Sleep_time_weekend_n= 12 if Sleep_time_weekend == 12
replace Sleep_time_weekend_n= 13 if Sleep_time_weekend == 13
replace Sleep_time_weekend_n= 14 if Sleep_time_weekend == 14
replace Sleep_time_weekend_n= 15 if Sleep_time_weekend == 15
replace Sleep_time_weekend_n= 18 if Sleep_time_weekend == 18
label define Sleep_time_weekend 1 " 1hr/night" 4 "4hr/night" 5 "5hr/
night" 6 "6hr/night" 7 "7hr/night" 8 "8hr/night" 9 "9hr/night" 10
"10hr/night" 11 "11hr/night" 12 "12hr/night" 13 "13hr/night" 14 "14hr/
night" 15 "15hr/night" 18 "18hr/night"
label values Sleep_time_weekend_n Sleep_time_weekend
tab Sleep_time_weekend_n

tab interupted_sleep
gen interupted_sleep_n=.
replace interupted_sleep_n =0 if interupted_sleep == 0
replace interupted_sleep_n =1 if interupted_sleep == 1
replace interupted_sleep_n =2 if interupted_sleep == 2
replace interupted_sleep_n =3 if interupted_sleep == 3
replace interupted_sleep_n =4 if interupted_sleep == 4
label define interupted_sleep 0 "Not at all" 1 "Slightly often" 2
"Often" 3 "Very often" 4 "Extremely often"
label value interupted_sleep_n interupted_sleep
tab interupted_sleep_n
tab interupted_sleep_bi
gen interupted_sleep_bi_n =.
replace interupted_sleep_bi_n = 0 if interupted_sleep_bi == 0
replace interupted_sleep_bi_n = 1 if interupted_sleep_bi == 1 |
interupted_sleep_bi == 2
```

```
label define interupted_sleep_bi 0 "No" 1 "Yes"
label values interupted_sleep_bi_n interupted_sleep_bi
tab interupted_sleep_bi_n

tab wakeup_night
gen wakeup_night_n=.
replace wakeup_night_n = 0 if wakeup_night == 0
replace wakeup_night_n = 1 if wakeup_night == 1
replace wakeup_night_n = 2 if wakeup_night == 2
label define wakeup_night 0 "No" 1 "Always" 2 "Sometimes"
label value wakeup_night_n wakeup_night
tab wakeup_night_n
tab wakeupnight_bin
gen wakeupnight_bin_n=.
replace wakeupnight_bin_n = 0 if wakeup_night_n == 0
replace wakeupnight_bin_n = 1 if wakeup_night_n == 1 | wakeup_night_n
== 2
label define wakeup_night_n 0 "No" 1 "Yes"
label value wakeupnight_bin_n wakeup_night_n
tab wakeupnight_bin_n

describe sleepy_day
tab sleepy_day
gen sleepy_day_n=.
replace sleepy_day_n = 0 if sleepy_day == 0
replace sleepy_day_n = 1 if sleepy_day == 1
replace sleepy_day_n = 2 if sleepy_day == 2
label define sleepy_day 0 "No" 1 "Yes" 2 "Sometimes"
label value sleepy_day_n sleepy_day
tab sleepy_day_n
tab sleepy_day_bi
gen sleepy_day_bi_n =.
replace sleepy_day_bi_n = 0 if sleepy_day_bi == 0
replace sleepy_day_bi_n = 1 if sleepy_day_bi == 1 | sleepy_day_bi == 2
label define sleepy_day_bi 0 "No" 1 "Yes"
label values sleepy_day_bi_n sleepy_day_bi
tab sleepy_day_bi_n

describe hard_wakeup
tab hard_wakeup
gen hard_wakeup_n=.
replace hard_wakeup_n = 0 if hard_wakeup == 0
replace hard_wakeup_n = 1 if hard_wakeup == 1
replace hard_wakeup_n = 2 if hard_wakeup == 2
label define hard_wakeup 0 "No" 1 "Yes" 2 "Sometimes"
label value hard_wakeup_n hard_wakeup
tab hard_wakeup_n
tab hard_wakeup_bi
gen hard_wakeup_bi_n =.
replace hard_wakeup_bi_n = 0 if hard_wakeup_bi == 0
```

```
replace hard_wakeup_bi_n = 1 if hard_wakeup_bi == 1 | hard_wakeup_bi
== 2
label define hard_wakeup_bi 0 "No" 1 "Yes"
label values hard_wakeup_bi_n hard_wakeup_bi
tab hard_wakeup_bi_n

describe diff_fall_asleep
tab diff_fall_asleep
gen diff_fall_asleep_n=.
replace diff_fall_asleep_n = 0 if diff_fall_asleep == 0
replace diff_fall_asleep_n = 1 if diff_fall_asleep == 1
replace diff_fall_asleep_n = 2 if diff_fall_asleep == 2
replace diff_fall_asleep_n = 3 if diff_fall_asleep == 3
replace diff_fall_asleep_n = 4 if diff_fall_asleep == 4
label define diff_fall_asleep 0 "Not at all" 1 "Slightly often" 2
"Often" 3 "Very often" 4 "Extremely often"
label value diff_fall_asleep_n diff_fall_asleep
tab diff_fall_asleep_n
tab diff_fall_asleep_bi
gen diff_fall_asleep_bi_n = .
replace diff_fall_asleep_bi_n = 0 if diff_fall_asleep_n == 0
replace diff_fall_asleep_bi_n = 1 if diff_fall_asleep_n == 1 |
diff_fall_asleep_n ==2 | diff_fall_asleep_n ==3 | diff_fall_asleep_n
==4
label define diff_fall_asleep_n 0 "Not at all" 1 " Yes"
label values diff_fall_asleep_bi_n diff_fall_asleep_n
tab diff_fall_asleep_bi_n


describe quality_sleep
tab quality_sleep
gen quality_sleep_n=.
replace quality_sleep_n = 0 if quality_sleep == 0
replace quality_sleep_n = 1 if quality_sleep == 1
replace quality_sleep_n = 2 if quality_sleep == 2
replace quality_sleep_n = 3 if quality_sleep == 3
replace quality_sleep_n = 4 if quality_sleep == 4
replace quality_sleep_n = 5 if quality_sleep == 5
label define quality_sleep 0 "I do not know" 1 "Very bad" 2 "Bad" 3
"Fair" 4 "Good" 5 "Excellent"
label value quality_sleep_n quality_sleep
tab quality_sleep_n

describe snore
tab snore
gen snore_n=.
replace snore_n = 0 if snore == 0
replace snore_n = 1 if snore == 1
replace snore_n = 2 if snore == 2
label define snore 0 "No" 1 "Yes" 2 "I do not konw"
```

```
label value snore_n snore
tab snore_n
tab snore_bi
gen snore_bi_n =.
replace snore_bi_n = 0 if snore_bi == 0
replace snore_bi_n = 1 if snore_bi == 1 | snore_bi == 2
label define snore_bi 0 "No" 1 "Yes"
label values snore_bi_n snore_bi
tab snore_bi_n

describe nap
tab nap
gen nap_n=.
replace nap_n = 0 if nap == 0
replace nap_n = 1 if nap == 1
replace nap_n = 2 if nap == 2
replace nap_n = 3 if nap == 3
label define nap 0 "No" 1 "Always" 2 "Sometimes" 3 "Others or I do not
know"
label value nap_n nap
tab nap_n
tab nap_bi
gen nap_bi_n =.
replace nap_bi_n = 0 if nap_bi == 0
replace nap_bi_n = 1 if nap_bi == 1 | nap_bi == 2
label define nap_bi 0 "No" 1 "Yes"
label values nap_bi_n nap_bi
tab nap_bi_n

describe sleep_apnea_max
tab sleep_apnea_max
gen sleep_apnea_max_n=.
replace sleep_apnea_max_n = 0 if sleep_apnea_max == 0
replace sleep_apnea_max_n = 1 if sleep_apnea_max == 1
label define sleep_apnea_max 0 "No" 1 "Yes"
label value sleep_apnea_max_n sleep_apnea_max
tab sleep_apnea_max_n

tab bed12bin
gen bed12bin_n =.
replace bed12bin_n = 0 if sleep_weekday == 21 | sleep_weekday == 22 |
sleep_weekday == 23
replace bed12bin_n = 1 if sleep_weekday == 1 | sleep_weekday == 2 |
sleep_weekday == 3 | sleep_weekday == 24 | sleep_weekday == 99
label define bed12bin 0 "Sleep before midnight" 1 "Sleep at or after
midnight"
label values bed12bin_n bed12bin
tab bed12bin_n

tab sleep_weekend_n
```

```
tab sleep_weekend
gen bed12_weekend_bi = .
replace bed12_weekend_bi = 0 if sleep_weekend_n == 21  |
sleep_weekend_n == 22  | sleep_weekend_n == 23
replace bed12_weekend_bi = 1 if sleep_weekend_n == 1 | sleep_weekend_n
== 2 | sleep_weekend_n == 3 | sleep_weekend_n == 4 | sleep_weekend_n
== 5 | sleep_weekend_n == 6 | sleep_weekend_n == 7 | sleep_weekend_n
== 8 | sleep_weekend_n == 9 | sleep_weekend_n == 24 | sleep_weekend_n
== 99
label define sleep_weekend_n 0 "Sleep before midnight" 1 "Sleep at or
after midnight"
label values bed12_weekend_bi sleep_weekend_n
tab bed12_weekend_bi

tab bed10_bin
gen bed10_bin_n = .
replace bed10_bin_n = 0 if sleep_weekday == 21 | sleep_weekday == 22
replace bed10_bin_n = 1 if sleep_weekday == 23 | sleep_weekday == 24 |
sleep_weekday == 1 | sleep_weekday == 2 | sleep_weekday == 3 |
sleep_weekday == 99
label define bed10_bin 0 "Sleep at or before 10 p.m" 1 "Sleep after 10
p.m"
label value bed10_bin_n bed10_bin
tab bed10_bin_n

tab sleep_duration6
gen sleep_duration6_n =.
replace sleep_duration6_n = 0 if Sleep_time_weekday == 1 |
Sleep_time_weekday == 2 | Sleep_time_weekday == 3 | Sleep_time_weekday
== 4 | Sleep_time_weekday == 5 | Sleep_time_weekday == 6
replace sleep_duration6_n = 1 if Sleep_time_weekday == 7 |
Sleep_time_weekday == 8 | Sleep_time_weekday == 9
label define sleep_duration6 0 "<or= 6hr/night" 1 "> 6hr/night"
label value sleep_duration6_n sleep_duration6
tab sleep_duration6_n

tab sleep_duration7
gen sleep_duration7_n =.
replace sleep_duration7_n = 0 if Sleep_time_weekday == 1 |
Sleep_time_weekday == 2 | Sleep_time_weekday == 3 | Sleep_time_weekday
== 4 | Sleep_time_weekday == 5 | Sleep_time_weekday == 6 |
Sleep_time_weekday == 7
replace sleep_duration7_n = 1 if Sleep_time_weekday == 8 |
Sleep_time_weekday == 9
label define sleep_duration7 0 "<or= 7hr/night" 1 "> 7hr/night"
label value sleep_duration7_n sleep_duration7
tab sleep_duration7_n

tab sleep_duration8
gen sleep_duration8_n =.
```

```
replace sleep_duration8_n = 0 if Sleep_time_weekday == 1 |
Sleep_time_weekday == 2 | Sleep_time_weekday == 3 | Sleep_time_weekday
== 4 | Sleep_time_weekday == 5 | Sleep_time_weekday == 6 |
Sleep_time_weekday == 7 | Sleep_time_weekday == 8
replace sleep_duration8_n = 1 if Sleep_time_weekday == 9
label define sleep_duration8 0 "<or= 8hr/night" 1 "> 8hr/night"
label value sleep_duration8_n sleep_duration8
tab sleep_duration8_n

tab WHOcat3
gen WHOcat3_n =.
replace WHOcat3_n = 1 if WHOcat3 == 1
replace WHOcat3_n = 2 if WHOcat3 == 2
replace WHOcat3_n = 3 if WHOcat3 == 3
replace WHOcat3_n = 4 if WHOcat3 == 4
label define WHOcat3 1 "Underweight" 2 "Normal weight" 3 "Overweight"
4 "Obese"
label value WHOcat3_n WHOcat3
tab WHOcat3_n

tab OBWHO3
gen OBWHO3_n =.
replace OBWHO3_n = 0 if OBWHO3 == 0
replace OBWHO3_n = 1 if OBWHO3 == 1
label define OBWHO3 0 "Non-obese" 1 "Obese"
label value OBWHO3_n OBWHO3
tab OBWHO3_n

tab electronic
gen electronic_n=.
replace electronic_n = 0 if electronic == 0
replace electronic_n = 1 if electronic == 1
label define electronic 0 " less than 3h/day" 1 "3hrs or more/day"
label values electronic_n electronic
tab electronic_n

tab Sleep_time_weekday_n
tab Sleep_time_weekend
gen Sleep_time_weekday5 = Sleep_time_weekday * 5
gen Sleep_time_weekend2 = Sleep_time_weekend * 2
tab Sleep_time_weekday5
gen Ave_Sleep_time_week =  Sleep_time_weekday5 + Sleep_time_weekend2
tab Ave_Sleep_time_week
gen AverageSleepDuration = Ave_Sleep_time_week / 7
tab AverageSleepDuration
gen AverageSleepDuration_bi=.
replace AverageSleepDuration_bi = 0 if AverageSleepDuration <= 6
replace AverageSleepDuration_bi = 1 if AverageSleepDuration > 6
tab AverageSleepDuration_bi
label define AverageSleepDuration 0 "< or = 6 hrs/day" 1 " > 6 hrs/
```

```
day"
label value AverageSleepDuration_bi AverageSleepDuration
tab AverageSleepDuration_bi
gen AverageSleepDuration_cat=.
replace AverageSleepDuration_cat=0 if AverageSleepDuration < 6
replace AverageSleepDuration_cat=1 if AverageSleepDuration >= 6 &
AverageSleepDuration < 7
replace AverageSleepDuration_cat=2 if AverageSleepDuration >= 7 &
AverageSleepDuration < 8
replace AverageSleepDuration_cat=3 if AverageSleepDuration >= 8 &
AverageSleepDuration< 9
replace AverageSleepDuration_cat=4 if AverageSleepDuration >= 9
tab AverageSleepDuration_cat

tab bed_weekend
tab bed_hour
gen bed_weekend2 = bed_weekend*2
gen bed_weekday5= bed_hour*5
gen bedtime_both2 = bed_weekend2 + bed_weekday5
replace bedtime_both2 = bedtime_both2/7
tab bedtime_both2
sum bedtime_both2, det
gen bed12 = bedtime_both2
replace bed12 = 0 if bed12 < 12.57143
replace bed12 = 1 if bed12 >= 12.57143
tab bed12
hist bed12bin_n
hist bed_weekend

drop bed12
gen bed12 = bedtime_both2
replace bed12 = 0 if bed12 < 12
replace bed12 = 1 if bed12 >= 12 & bed12 <=16
tab bed12


*gen bedtimeWEcat = bed_weekend
*replace bedtimeWEcat = 0 if bedtimeWEcat <=11
*replace bedtimeWEcat = 1 if bedtimeWEcat >11 & bedtimeWEcat <=12
*replace bedtimeWEcat = 2 if bedtimeWEcat >12 & bedtimeWEcat <=16
*tab bedtimeWEcat

*****************************************************************
*****************************************************************
gen bedtimeWEcat2 = bed_weekend
replace bedtimeWEcat2 = 0 if bedtimeWEcat2 <=11
replace bedtimeWEcat2 = 1 if bedtimeWEcat2 >11 & bedtimeWEcat2 <=13
replace bedtimeWEcat2 = 2 if bedtimeWEcat2 >13 & bedtimeWEcat2 <=16
tab bedtimeWEcat2
*****************************************************************
```

```
****************************************************************

*gen bedtimeWDcat = bed_hour
*tab bedtimeWDcat
*replace bedtimeWDcat = 0 if bedtimeWDcat <=11
*replace bedtimeWDcat = 1 if bedtimeWDcat >11 & bedtimeWDcat <=12
*replace bedtimeWDcat = 2 if bedtimeWDcat >12 & bedtimeWDcat <=16
*tab bedtimeWDcat

****************************************************************
****************************************************************
gen bedtimeWDcat2 = bed_hour
tab bedtimeWDcat2
replace bedtimeWDcat2 = 0 if bedtimeWDcat2 <=11
replace bedtimeWDcat2 = 1 if bedtimeWDcat2 >11 & bedtimeWDcat2 <=13
replace bedtimeWDcat2 = 2 if bedtimeWDcat2 >13 & bedtimeWDcat2 <=16
tab bedtimeWDcat2
****************************************************************
****************************************************************

gen bedtime123= bedtime_both2
replace bedtime123 = 0 if bedtime123 <=11
replace bedtime123 = 1 if bedtime123 > 11 & bedtime123 <=12
replace bedtime123 = 2 if bedtime123 >12 & bedtime123<=15.5
tab bedtime123

gen bedtimecat = .
replace bedtimecat = 0 if bedtime_both2 < 12
replace bedtimecat = 1 if bedtime_both2 >= 12 & bedtime_both2 <13
replace bedtimecat = 2 if bedtime_both2 >= 13 & bedtime_both2 <15.5
tab bedtimecat
*********************************************************************
*******************************************
gen bedtimecat2 = .
replace bedtimecat2 = 0 if bedtime_both2 < 12
replace bedtimecat2 = 1 if bedtime_both2 >= 12 & bedtime_both2 <=13
replace bedtimecat2 = 2 if bedtime_both2 > 13 & bedtime_both2 <15.5
tab bedtimecat2
********************************************
*********************************************************************


tab vediogames
gen vediogames_bi = .
replace vediogames_bi = 0 if vediogames == 0 | vediogames == 1 |
vediogames == 2
replace vediogames_bi = 1 if vediogames == 3 | vediogames == 4 |
vediogames == 5 | vediogames == 6
label define vediogames 0 "Less or equal to 2 hrs" 1 " More than 2
hours"
```

```
label value vediogames_bi vediogames
tab vediogames_bi

tab bed12bin_n
tab bed12bin_n, nolabel

*Sleep dept is the difference beween sleep duration (weekends -
weekdays
gen sleepdept = Sleep_time_weekend_n - Sleep_time_weekday_n
tab sleepdept
gen sleepdeptcat = .
replace sleepdeptcat = 0 if sleepdept <= 0
replace sleepdeptcat = 1 if sleepdept >= 1 & sleepdept <=11
tab sleepdeptcat

*Severe sleep debt =sleep debt more than 90 minutes ( we have the data
for the 2 hours i.e 120 minutes)
gen sleepdeptt = sleepdept
tab sleepdeptt
gen sleepdeptsevere = .
replace sleepdeptsevere = 0 if sleepdeptt <= 1
replace sleepdeptsevere = 1 if sleepdeptt >= 2 & sleepdeptt <=11
tab sleepdeptsevere

*Social getlag is the difference beween bedtime (weekday - weekend)
gen social_getlag= bed_weekend - bed_hour
gen socialgetlag_bi = social_getlag
tab socialgetlag_bi
sum socialgetlag_bi, detail
replace socialgetlag_bi = 0 if socialgetlag_bi < 1.431818
replace socialgetlag_bi = 1 if socialgetlag_bi >= 1.431818 &
social_getlag<= 6
tab socialgetlag_bi

tab social_getlag
gen SJL_bi = social_getlag
sum SJL_bi, detail
replace SJL_bi = 0 if SJL_bi < 1
replace SJL_bi = 1 if SJL_bi >= 1& SJL_bi <= 6
tab SJL_bi

gen Abscent_circadian = .
replace Abscent_circadian = 0 if vediogames_bi == 0 & bed12bin_n == 0
| vediogames_bi == 0 & bed12bin_n == 1 | vediogames_bi == 1 &
bed12bin_n == 0
replace Abscent_circadian = 1 if vediogames_bi == 1 & bed12bin_n == 1
tab Abscent_circadian
* Abeeer abscent circadian means = sleeping late after or at midnight
and exposed to late time light = the circadian rhythm got disturbed
```

```
tab sleep_duration6
tab bed12bin_n
gen timeandduration = .
replace timeandduration = 0 if sleep_duration6 == 1 & bed12bin_n == 0
replace timeandduration = 1 if sleep_duration6 == 0 & bed12bin_n == 0
replace timeandduration = 2 if sleep_duration6 == 0 & bed12bin_n == 1
replace timeandduration = 3 if sleep_duration6 == 1 & bed12bin_n == 1
tab timeandduration

*************************
tab AverageSleepDuration
gen ASD6 = .
replace ASD6 = 0 if AverageSleepDuration <= 6
replace ASD6 = 1 if AverageSleepDuration > 6 & AverageSleepDuration
<11
tab ASD6

tab bedtime_both2
gen BT12 = .
replace BT12 = 0 if bedtime_both2 <12
replace BT12 = 1 if bedtime_both2 >=12 & bedtime_both2 <15.5
tab BT12

tab ASD6
tab BT12
gen durtime =.
replace durtime = 0 if ASD == 0 & BT12 == 1
replace durtime = 1 if ASD == 1 & BT12 == 0
replace durtime = 2 if ASD == 0 & BT12 == 0
replace durtime = 3 if ASD == 1 & BT12 == 1
tab durtime

gen SGood = durtime >=1 & durtime ! = 0
tab SGood

drop SGood
gen SBad = durtime
replace SBad = 0 if durtime == 0
************************************



*Food
tab coffee
gen coffee_n = .
replace coffee_n = 0 if coffee < 1
replace coffee_n = 1 if coffee >= 1 & coffee <= 4
tab coffee_n

*Dinner
```

```
tab dinner
tab lunch
tab coffee

tab fied_food_restaurant
gen friedfood_restaurant =.
replace friedfood_restaurant = 0 if fied_food_restaurant <1
replace friedfood_restaurant = 1 if fied_food_restaurant >= 1 &
fied_food_restaurant <=3
tab friedfood_restaurant

gen bed12new= bedtime_both2
replace bed12new= 0 if bedtime_both2 < 12.49
replace bed12new= 1 if bedtime_both2 > = 12.49
label define bedtime_both2 0 "less than 12.49" 1 "at or more than
12.49"
label value bedtime_both2 bed12new
tab bed12new

************************Sleep variables
coding*****************************************
*AverageSleepDuration = (continuous) avearge sleep duration for both
weekends and weekdays
*Sleep_time_weekday_n = (continuous) sleep duration on weekdays
*Sleep_time_weekend_n = (continuous) sleep duration on weekends
*bedtime_both2 = (continuous) average bedtime for both weekends and
seekdays
*bed_hour = (continuous) bedtime on weekdays
*bed_weekend = (continuous) bedtime on weekends
*bed12 = (binary) average bedtime for both weekends and weekdays
*bed12bin_n = (binary) bedtime for weekdays
*bed12_weekend_bi = (binary) bedtime for weekends
*sleepdept = (continuous) difference in sleep duration between
weekdays and weekends
*sleepdeptcat = (binary) more than 60 minutes
*sleepdeptsevere = (binary) more than 120 monutes (the definition for
severe sleepdebt is > 90 minites, but we do not have the data for
that)
*social_getlag = (continuous) differnce in bedtime between weekdays
and weekedns
*socialgetlag_bi = (binary) social jetlag divided by the mean
*other classification not for this paper == bedtime123 = (categorical)
for average bedtime on both weekends and weekdays
*bedtimecat = (categorical) for average bedtime on both weekends and
weekdays
*bedtimeWDcat = (categorical) for bedtime weekdays
*bedtimeWEcat = (categorical) for bedtime weekends
*SJL_bi = SJL by <1hour and eqaual or more to 1 hour
*durtime = 4 categories for average bedtime and average sleep duration
Used for this thesis:
```

```
bedtimecat2 = (categorrical) for average bedtime on both weekends and
weekdays
bedtimeWDcat2 = (categorical) for bedtime weekdays
bedtimeWEcat2 = (categorical) for bedtime weekends
**********************************************************************
**********
*****Checking for normality of distribution for the outcome variables:
==> all outcomes not normally distributed
*All Above are skewed to the right==> log10
tab Adiponectin_P3
hist Adiponectin_P3
graph box Adiponectin_P3

tab Adiponectin_Se_P3
hist Adiponectin_Se_P3
graph box Adiponectin_Se_P3

tab UCN3_Se_P3
hist UCN3_Se_P3
graph box UCN3_Se_P3

tab IL6_P3
hist IL6_P3
graph box IL6_P3

tab HSCRPugml
hist HSCRPugml
graph box HSCRPugml

tab IL10_P3
hist IL10_P3
graph box IL10_P3

sum IL8_P3, detail
hist IL8_P3

hist Leptin_P3

gen logIL6_P3 = log(IL6_P3)
gen logIL10 = log(IL10_P3)
gen logIL10_Se = log(IL10_Se_P3)

tab IL6_Se_P3
hist IL6_Se_P3
graph box IL6_Se_P3


*One-Sample Kolmogorov-Smirnov Test to check for normality of
distribution: (if significant==> not normally distributed, so if not
significant ==> normally distributed)
```

```
ksmirnov X = normal((X-mean )/ SD )
sum CRP_P3
ksmirnov CRP_P3 = normal((CRP_P3-851.9256 )/ 1568.478 )

sum HSCRPugml
ksmirnov HSCRPugml = normal((HSCRPugml-2.788032)/ 3.233532 )

sum IL6_P3
ksmirnov IL6_P3 = normal((IL6_P3-10.48274 )/34.54921 )

sum IL6_Se_P3
ksmirnov IL6_Se_P3 = normal((IL6_Se_P3- 1.202366)/.7201484)

sum IL8_P3
ksmirnov IL8_P3 = normal((IL8_P3-  663.2398)/ 663.2398)

sum IL8_Se_P3
ksmirnov IL8_Se_P3 = normal((IL8_Se_P3- 7.184563)/ 12.27899 )

sum IL10_P3
ksmirnov IL10_P3 = normal((IL10_P3- .52)/ .2859843 )

sum IL10_Se_P3
ksmirnov IL10_Se_P3 = normal((IL10_Se_P3- 1.102174 )/1.432953 )

sum VEGF_P3
ksmirnov VEGF_P3 = normal((VEGF_P3- 705.6112)/ 341.0367 )
*Normally distributed

sum VEGF_Se_P3
ksmirnov VEGF_Se_P3 = normal((VEGF_Se_P3- 87.07759 )/76.03974 )

sum MCP1_P3
ksmirnov MCP1_P3 = normal((MCP1_P3- 705.6112)/ 341.0367 )

sum MCP1_Se_P3
ksmirnov MCP1_Se_P3 = normal((MCP1_Se_P3- 312.7764 )/165.2888  )

sum Adiponectin_P3
ksmirnov Adiponectin_P3 = normal((Adiponectin_P3- 8163.355)/ 11342.3)

sum Adiponectin_Se_P3
ksmirnov Adiponectin_Se_P3 = normal((Adiponectin_Se_P3- 9893942 )/
8712548 )

sum Leptin_P3
ksmirnov Leptin_P3 = normal((Leptin_P3- 202.0996)/ 130.5632)

sum Leptin_Se_P3
ksmirnov Leptin_Se_P3 = normal((Leptin_Se_P3- 23872.53 )/23786.19 )
```

```
sum Insulin_P3
ksmirnov Insulin_P3 = normal((Insulin_P3- 397.9171)/ 358.0936 )

sum Insulin_Se_P3
ksmirnov Insulin_Se_P3 = normal((Insulin_Se_P3- 1005.961 )/ 942.6419 )

sum social_getlag
ksmirnov Insulin_Se_P3 = normal((Insulin_Se_P3- 1.431818 )/1.479551  )
gen logsocial_getlag = log(social_getlag)


gen logIL6_P3 = log(IL6_P3)
gen logIL10 = log(IL10_P3)
gen logIL10_Se = log(IL10_Se_P3)


****Descriptive tables:
*Continuos variables:
sum AgeP3, detail
sum BMI3, detail
sum Waist_cm3, detail

*Biomarkers:
sum Adiponectin_P3, detail
sum Adiponectin_Se_P3, detail

sum Leptin_P3, detail
sum Leptin_Se_P3, detail

sum Insulin_P3, detail
sum Insulin_Se_P3, detail

sum IL6_P3, detail
sum IL6_Se_P3,detail

sum IL8_P3, detail
sum IL8_Se_P3, detail

sum IL10_P3, detail
sum IL10_Se_P3, detail

sum VEGF_P3, detail
sum VEGF_Se_P3, detail

sum MCP1_P3, detail
sum MCP1_Se_P3, detail

sum CRP_P3, detail
sum CRP_Se_P3, detail
```

```
sum HSCRPugml, detail

sum DineOut, detail
sum EatHome, detail

*Sleep variables:
sum Sleep_time_weekday_n, detail
sum Sleep_time_weekend_n, detail
sum AverageSleepDuration, detail

sum bed_hour, detail
sum bed_weekend, detail
sum bedtime_both2, detail

sum sleepdept, detail
sum social_getlag, detail
sum nap_hours, detail

tab bed12
tab bed12bin_n
tab bed12_weekend_bi
tab bedtime123
tab bedtimeWDcat
tab bedtimeWEcat
**********************************************************************
*********
Descriptive: Continuous
tabstat AgeP3,  s(n mean SD) c(s) by(bedtimecat2)
tabstat BMI3,  s(n mean SD) c(s) by(bedtimecat2)
tabstat Waist_cm3,  s(n mean SD) c(s) by(bedtimecat2)
tabstat Adiponectin_P3,  s(n mean SD) c(s) by(bedtimecat2)
tabstat Adiponectin_Se_P3,  s(n mean SD) c(s) by(bedtimecat2)
tabstat Leptin_P3,  s(n mean SD) c(s) by(bedtimecat2)
tabstat Leptin_Se_P3,  s(n mean SD) c(s) by(bedtimecat2)
tabstat Insulin_P3,  s(n mean SD) c(s) by(bedtimecat2)
tabstat Insulin_Se_P3,  s(n mean SD) c(s) by(bedtimecat2)
tabstat IL6_P3,  s(n mean SD) c(s) by(bedtimecat2)
tabstat IL6_Se_P3,  s(n mean SD) c(s) by(bedtimecat2)
tabstat IL8_P3,  s(n mean SD) c(s) by(bedtimecat2)
tabstat IL8_Se_P3,  s(n mean SD) c(s) by(bedtimecat2)
tabstat IL10_P3,  s(n mean SD) c(s) by(bedtimecat2)
tabstat IL10_Se_P3,  s(n mean SD) c(s) by(bedtimecat2)
tabstat VEGF_P3,  s(n mean SD) c(s) by(bedtimecat2)
tabstat VEGF_Se_P3,  s(n mean SD) c(s) by(bedtimecat2)
tabstat MCP1_P3,  s(n mean SD) c(s) by(bedtimecat2)
tabstat MCP1_Se_P3,  s(n mean SD) c(s) by(bedtimecat2)
tabstat CRP_P3,  s(n mean SD) c(s) by(bedtimecat2)
tabstat CRP_Se_P3,  s(n mean SD) c(s) by(bedtimecat2)
tabstat HSCRPugml,  s(n mean SD) c(s) by(bedtimecat2)
tabstat DineOut,  s(n mean SD) c(s) by(bedtimecat2)
```

```
tabstat EatHome,  s(n mean SD) c(s) by(bedtimecat2)

tabstat AverageSleepDuration,  s(n mean SD) c(s) by(bedtimecat2)
tabstat Sleep_time_weekday_n,  s(n mean SD) c(s) by(bedtimecat2)
tabstat Sleep_time_weekend_n,  s(n mean SD) c(s) by(bedtimecat2)
tabstat bedtime_both2,  s(n mean SD) c(s) by(bedtimecat2)
tabstat bed_hour,  s(n mean SD) c(s) by(bedtimecat2)
tabstat bed_weekend,  s(n mean SD) c(s) by(bedtimecat2)
tabstat sleepdept,  s(n mean SD) c(s) by(bedtimecat2)
tabstat social_getlag,  s(n mean SD) c(s) by(bedtimecat2)
tabstat nap_hours, s(n mean SD) c(s) by(bedtimecat2)


tabstat DineOut,  s(n mean SD) c(s) by(bedtimecat2)
tabstat EatHome,  s(n mean SD) c(s) by(bedtimecat2)

sum AgeP3, detail

* Descriptive Binary:
tab bedtimecat2
tab sex_n bedtimecat2, column freq chi2 exact
tab WHOcat3_n bedtimecat2, column freq chi2 exact
tab OBWHO3_n bedtimecat2, column freq chi2 exact
tab nap_bi_n bedtimecat2, column freq chi2 exact
tab snore_bi_n bedtimecat2, column freq chi2 exact
tab interupted_sleep_bi_n bedtimecat2, column freq chi2 exact
tab hard_wakeup_bi_n bedtimecat2, column freq chi2 exact
tab diff_fall_asleep_bi_n bedtimecat2, column freq chi2 exact
tab sleepy_day_bi_n bedtimecat2, column freq chi2 exact
tab wakeupnight_bin_n bedtimecat2, column freq chi2 exact
tab electronic_n bedtimecat2, column freq chi2 exact
tab medical_history_n bedtimecat2, column freq chi2 exact
tab sleepdeptcat bedtimecat2, column freq chi2 exact
tab sleepdeptsevere bedtimecat2, column freq chi2 exact
tab socialgetlag_bi bedtimecat2, column freq chi2 exact
tab Abscent_circadian bedtimecat2, column freq chi2 exact
tab physical_act bedtimecat2, column freq chi2 exact
tab coffee_n bedtimecat2, column freq chi2 exact
tab dinner bedtimecat2, column freq chi2 exact
tab lunch bedtimecat2, column freq chi2 exact
tab friedfood_restaurant bedtimecat2, column freq chi2 exact


********************************************
ANOVA vs Kraskel wallis
oneway AgeP3 bedtimecat2, tabulate
oneway BMI3 bedtimecat2, tabulate
oneway Waist_cm3 bedtimecat2, tabulate

kwa CRP_P3, by(bedtimecat2)
```

```
Levens test for homodcadasticity
robvar CRP_P3, by(bedtimecat2)
Shapiro test for narmality distribution: if stat sig: not normally
distributed
swilk CRP_P3 bedtimecat2
*********************************************************************
**********************
*****See the correlation between sleep time and duration on (weekend,
weekday, and average) using interaction term between the two or
categorization combining the two sleep variables.
First: Correlation
*WEEKEND
pwcorr Sleep_time_weekend_n bed_weekend, star (.05)
*there in negative significant (*) correlation
*WEEKDAY
pwcorr Sleep_time_weekday_n bed_hour, star (.05)
*there in negative significant (*) correlation
*Average
pwcorr AverageSleepDuration bedtime_both2
*there in negative significant (*) correlation
*Interpretation: the earlier the bedtime, the more sleeping
duration*********
*********************************************************************
**********
*With spearman for ordinal variables, no need to do the log
transformation as it will compensate for it
spearman CRP_P3 HSCRPugml, stats(rho p)
spearman IL6_P3 IL6_Se_P3, stats(rho p)
spearman IL8_P3 IL8_Se_P3, stats(rho p)
spearman IL10_P3 IL10_Se_P3, stats(rho p)
spearman Adiponectin_P3 Adiponectin_Se_P3, stats(rho p)
spearman Leptin_P3 Leptin_Se_P3, stats(rho p)
spearman Insulin_P3 Insulin_Se_P3, stats(rho p)
spearman VEGF_P3 VEGF_Se_P3, stats(rho p)
spearman MCP1_P3 MCP1_Se_P3, stats(rho p)

* With pearson correlation for continuous variables==> log
transformation because not normally distributed
pwcorr logCRP_P3 logHSCRPugml, sig star(.05) obs
pwcorr logIL6_P3 logIL6_Se_P3, sig star(.05) obs
pwcorr logIL8_P3 logIL8_Se_P3, sig star(.05) obs
pwcorr logIL10_P3 logIL10_Se_P3, sig star(.05) obs
pwcorr VEGF_P3 logVEGF_Se_P3, sig star(.05) obs
pwcorr logMCP1_P3 logMCP1_Se_P3, sig star(.05) obs
pwcorr logAdiponectin_P3 logAdiponectin_Se_P3, sig star(.05) obs
pwcorr logLeptin_P3 logLeptin_Se_P3, sig star(.05) obs
pwcorr logInsulin_P3 logInsulin_Se_P3, sig star(.05) obs
```

```
===================================================================================================
===========================
*******************************PAPER
1*******************************************************
*Dr.Finkelman: Random Effect Model to account for the School variable
(it is 15 schools and we use REM to account for the school effect to
be at Random)

*use estat ic command to check which model to select:
estat ic
to compare between models using AIC and BIC , where lower values are
the modle with the best fit.


* logCRP_P3
*MIXED EFFECT MODEL
xtmixed logCRP_P3 AverageSleepDuration sex_n AgeP3 nap_bi_n
i.medical_history_n vediogames DineOut, || School:
xtmixed logCRP_P3 Sleep_time_weekday_n sex_n AgeP3 nap_bi_n
i.medical_history_n vediogames DineOut, || School:
xtmixed logCRP_P3 Sleep_time_weekend_n sex_n AgeP3 nap_bi_n
i.medical_history_n vediogames DineOut, || School:

xtmixed logCRP_P3 bedtime_both2 sex_n AgeP3 nap_bi_n
i.medical_history_n vediogames DineOut, || School:
xtmixed logCRP_P3 bed_hour sex_n AgeP3 nap_bi_n i.medical_history_n
vediogames DineOut, || School:
xtmixed logCRP_P3 bed_weekend sex_n AgeP3 nap_bi_n i.medical_history_n
vediogames DineOut, || School:

xtmixed logCRP_P3 i.bedtimecat2 sex_n AgeP3 nap_bi_n
i.medical_history_n vediogames DineOut, || School:

xtmixed logCRP_P3 i.sleepdeptcat sex_n AgeP3 nap_bi_n
i.medical_history_n vediogames DineOut, || School:
xtmixed logCRP_P3 i.sleepdeptsevere sex_n AgeP3 nap_bi_n
i.medical_history_n vediogames DineOut, || School:

xtmixed logCRP_P3 i.socialgetlag_bi sex_n AgeP3 nap_bi_n
i.medical_history_n vediogames DineOut, || School:
xtmixed logCRP_P3 social_getlag sex_n AgeP3 nap_bi_n
i.medical_history_n vediogames DineOut, || School:

xtmixed logCRP_P3 i.durtime sex_n AgeP3 nap_bi_n i.medical_history_n
vediogames DineOut, || School:
```

```
xtmixed logCRP_P3 i.timeandduration sex_n AgeP3 nap_bi_n
i.medical_history_n vediogames DineOut, || School:
xtmixed logCRP_P3 i.bedtimeWDcat2 sex_n AgeP3 nap_bi_n
i.medical_history_n vediogames DineOut, || School:
xtmixed logCRP_P3 i.bedtimeWEcat2 sex_n AgeP3 nap_bi_n
i.medical_history_n vediogames DineOut, || School:
xtmixed logCRP_P3 i.SJL_bi sex_n AgeP3 nap_bi_n i.medical_history_n
vediogames DineOut, || School:
xtmixed logCRP_P3 bed12new sex_n AgeP3 nap_bi_n i.medical_history_n
vediogames DineOut, || School:
xtmixed logCRP_P3 bedtimecat sex_n AgeP3 nap_bi_n i.medical_history_n
vediogames DineOut, || School:
xtmixed logCRP_P3 bed12 sex_n AgeP3 nap_bi_n i.medical_history_n
vediogames DineOut, || School:
xtmixed logCRP_P3 bed12bin_n sex_n AgeP3 nap_bi_n i.medical_history_n
vediogames DineOut, || School:
xtmixed logCRP_P3 bed12_weekend_bi sex_n AgeP3 nap_bi_n
i.medical_history_n vediogames DineOut, || School:
xtmixed logCRP_P3 i.bedtime123 sex_n AgeP3 nap_bi_n
i.medical_history_n vediogames DineOut, || School:
xtmixed logCRP_P3 i.bedtimeWDcat sex_n AgeP3 nap_bi_n
i.medical_history_n vediogames DineOut, || School:
xtmixed logCRP_P3 i.bedtimeWEcat sex_n AgeP3 nap_bi_n
i.medical_history_n vediogames DineOut, || School:
xtmixed logCRP_P3 sleepdept sex_n AgeP3 nap_bi_n i.medical_history_n
vediogames DineOut, || School:
xtmixed logCRP_P3 social_getlag  sex_n AgeP3 nap_bi_n
i.medical_history_n vediogames DineOut, || School:
xtmixed logCRP_P3 social_getlag i.durtime  sex_n AgeP3 nap_bi_n
i.medical_history_n vediogames DineOut, || School:
xtmixed logCRP_P3 i.socialgetlag_bi sex_n AgeP3 nap_bi_n
i.medical_history_n vediogames BMI3 DineOut, || School:
xtmixed logCRP_P3 i.sleepdeptsevere sex_n AgeP3 nap_bi_n
i.medical_history_n vediogames BMI3 DineOut, || School:
xtmixed logCRP_P3 i.sleepdeptcat sex_n AgeP3 nap_bi_n
i.medical_history_n vediogames BMI3 DineOut, || School:


*HSCRP (High sensistivity Serum CRP)
*MIXED EFFECT MODELS
gen logHSCRPugml = log(HSCRPugml)

xtmixed logHSCRPugml AverageSleepDuration sex_n AgeP3 nap_bi_n
i.medical_history_n vediogames DineOut, || School:
xtmixed logHSCRPugml Sleep_time_weekday_n sex_n AgeP3 nap_bi_n
i.medical_history_n vediogames DineOut, || School:
xtmixed logHSCRPugml Sleep_time_weekend_n sex_n AgeP3 nap_bi_n
i.medical_history_n vediogames DineOut, || School:
```

```
xtmixed logHSCRPugml bedtime_both2 sex_n AgeP3 nap_bi_n
i.medical_history_n vediogames DineOut, || School:
xtmixed logHSCRPugml bed_hour sex_n AgeP3 nap_bi_n i.medical_history_n
vediogames DineOut, || School:
xtmixed logHSCRPugml bed_weekend sex_n AgeP3 nap_bi_n
i.medical_history_n vediogames DineOut, || School:

xtmixed logHSCRPugml i.bedtimecat2 sex_n AgeP3 nap_bi_n
i.medical_history_n vediogames DineOut, || School:

xtmixed logHSCRPugml i.sleepdeptcat sex_n AgeP3 nap_bi_n
i.medical_history_n vediogames DineOut, || School:
xtmixed logHSCRPugml i.sleepdeptsevere sex_n AgeP3 nap_bi_n
i.medical_history_n vediogames DineOut, || School:

xtmixed logHSCRPugml i.socialgetlag_bi sex_n AgeP3 nap_bi_n
i.medical_history_n vediogames DineOut, || School:
xtmixed logHSCRPugml social_getlag sex_n AgeP3 nap_bi_n
i.medical_history_n vediogames DineOut, || School:

xtmixed logHSCRPugml i.timeandduration sex_n AgeP3 nap_bi_n
i.medical_history_n vediogames DineOut, || School:


xtmixed logHSCRPugml i.SJL_bi sex_n AgeP3 nap_bi_n i.medical_history_n
vediogames DineOut, || School:
xtmixed logHSCRPugml i.durtime sex_n AgeP3 nap_bi_n
i.medical_history_n vediogames DineOut, || School:
xtmixed logHSCRPugml i.bedtimeWDcat2 sex_n AgeP3 nap_bi_n
i.medical_history_n vediogames DineOut, || School:
xtmixed logHSCRPugml i.bedtimeWEcat2 sex_n AgeP3 nap_bi_n
i.medical_history_n vediogames DineOut, || School:
xtmixed logHSCRPugml i.sleepdeptcat sex_n AgeP3 nap_bi_n
i.medical_history_n vediogames BMI3 DineOut, || School:
xtmixed logHSCRPugml i.sleepdeptsevere sex_n AgeP3 nap_bi_n
i.medical_history_n vediogames BMI3 DineOut, || School:
xtmixed logHSCRPugml i.socialgetlag_bi sex_n AgeP3 nap_bi_n
i.medical_history_n vediogames BMI3 DineOut, || School:


*CRP serum not high sensitivity:
xtmixed logCRP_Se_P3 AverageSleepDuration sex_n AgeP3 nap_bi_n
i.medical_history_n vediogames DineOut, || School:
xtmixed logCRP_Se_P3 Sleep_time_weekday_n sex_n AgeP3 nap_bi_n
i.medical_history_n vediogames DineOut, || School:
xtmixed logCRP_Se_P3 Sleep_time_weekend_n sex_n AgeP3 nap_bi_n
i.medical_history_n vediogames DineOut, || School:

xtmixed logCRP_Se_P3 bedtime_both2 sex_n AgeP3 nap_bi_n
i.medical_history_n vediogames DineOut, || School:
```

```
xtmixed logCRP_Se_P3 bed_hour sex_n AgeP3 nap_bi_n i.medical_history_n
vediogames DineOut, || School:
xtmixed logCRP_Se_P3 bed_weekend sex_n AgeP3 nap_bi_n
i.medical_history_n vediogames DineOut, || School:

xtmixed logCRP_Se_P3 i.bedtimecat2 sex_n AgeP3 nap_bi_n
i.medical_history_n vediogames DineOut, || School:

xtmixed logCRP_Se_P3 i.sleepdeptcat sex_n AgeP3 nap_bi_n
i.medical_history_n vediogames DineOut, || School:
xtmixed logCRP_Se_P3 i.sleepdeptsevere sex_n AgeP3 nap_bi_n
i.medical_history_n vediogames DineOut, || School:

xtmixed logCRP_Se_P3 i.socialgetlag_bi sex_n AgeP3 nap_bi_n
i.medical_history_n vediogames DineOut, || School:
xtmixed logCRP_Se_P3 social_getlag sex_n AgeP3 nap_bi_n
i.medical_history_n vediogames DineOut, || School:

xtmixed logCRP_Se_P3 i.durtime sex_n AgeP3 nap_bi_n
i.medical_history_n vediogames DineOut, || School:


xtmixed logCRP_Se_P3 i.bedtimeWDcat2 sex_n AgeP3 nap_bi_n
i.medical_history_n vediogames DineOut, || School:
xtmixed logCRP_Se_P3 i.bedtimeWEcat2 sex_n AgeP3 nap_bi_n
i.medical_history_n vediogames DineOut, || School:
xtmixed logCRP_Se_P3 i.sleepdeptcat sex_n AgeP3 nap_bi_n
i.medical_history_n vediogames BMI3 DineOut, || School:
xtmixed logCRP_Se_P3 i.sleepdeptsevere sex_n AgeP3 nap_bi_n
i.medical_history_n vediogames BMI3 DineOut, || School:
xtmixed logCRP_Se_P3 i.socialgetlag_bi sex_n AgeP3 nap_bi_n
i.medical_history_n vediogames BMI3 DineOut, || School:
xtmixed logCRP_Se_P3 i.SJL_bi sex_n AgeP3 nap_bi_n i.medical_history_n
vediogames DineOut, || School:


**************************

*Third: IL6 Saliva ==> log
xtmixed logIL6_P3 AverageSleepDuration sex_n AgeP3 nap_bi_n
i.medical_history_n vediogames DineOut, || School:
xtmixed logIL6_P3 Sleep_time_weekday sex_n AgeP3 nap_bi_n
i.medical_history_n vediogames DineOut, || School:
*Significant
xtmixed logIL6_P3 Sleep_time_weekend_n sex_n AgeP3 nap_bi_n
i.medical_history_n vediogames DineOut, || School:

xtmixed logIL6_P3 bedtime_both2 sex_n AgeP3 nap_bi_n
i.medical_history_n vediogames DineOut, || School:
xtmixed logIL6_P3 bed_hour sex_n AgeP3 nap_bi_n i.medical_history_n
```

```
vediogames DineOut, || School:
xtmixed logIL6_P3 bed_weekend sex_n AgeP3 nap_bi_n i.medical_history_n
vediogames DineOut, || School:
*Significant

xtmixed logIL6_P3 i.bedtimecat2 sex_n AgeP3 nap_bi_n
i.medical_history_n vediogames DineOut, || School:
xtmixed logIL6_P3 i.bedtimeWDcat2 sex_n AgeP3 nap_bi_n
i.medical_history_n vediogames DineOut, || School:
*sig
xtmixed logIL6_P3 i.bedtimeWEcat2 sex_n AgeP3 nap_bi_n
i.medical_history_n vediogames DineOut, || School:

xtmixed logIL6_P3 i.bedtimecat2 sex_n AgeP3 nap_bi_n
i.medical_history_n vediogames BMI3 DineOut, || School:

xtmixed logIL6_P3 bed12new sex_n AgeP3 nap_bi_n i.medical_history_n
vediogames DineOut, || School:
xtmixed logIL6_P3 bedtimecat sex_n AgeP3 nap_bi_n i.medical_history_n
vediogames DineOut, || School:

xtmixed logIL6_P3 bed12 sex_n AgeP3 nap_bi_n i.medical_history_n
vediogames DineOut, || School:
*Significant
xtmixed logIL6_P3 bed12bin_n sex_n AgeP3 nap_bi_n i.medical_history_n
vediogames DineOut, || School:
*Significant
xtmixed logIL6_P3 bed12_weekend_bi sex_n AgeP3 nap_bi_n
i.medical_history_n vediogames DineOut, || School:

xtmixed logIL6_P3 i.socialgetlag_bi sex_n AgeP3 nap_bi_n
i.medical_history_n vediogames DineOut, || School:
*Significant
xtmixed logIL6_P3 i.SJL_bi sex_n AgeP3 nap_bi_n i.medical_history_n
vediogames DineOut, || School:
xtmixed logIL6_P3 social_getlag sex_n AgeP3 nap_bi_n
i.medical_history_n vediogames DineOut, || School:


xtmixed logIL6_P3 i.durtime sex_n AgeP3 nap_bi_n i.medical_history_n
vediogames DineOut, || School:


xtmixed logIL6_P3 i.bedtime123 sex_n AgeP3 nap_bi_n
i.medical_history_n vediogames DineOut, || School:
*Significant
xtmixed logIL6_P3 i.bedtimeWDcat sex_n AgeP3 nap_bi_n
i.medical_history_n vediogames DineOut, || School:
*Significant
xtmixed logIL6_P3 i.bedtimeWEcat sex_n AgeP3 nap_bi_n
```

```
i.medical_history_n vediogames DineOut, || School:

xtmixed logIL6_P3 sleepdept sex_n AgeP3 nap_bi_n i.medical_history_n
vediogames DineOut, || School:
*Significant
xtmixed logIL6_P3 i.sleepdeptcat sex_n AgeP3 nap_bi_n
i.medical_history_n vediogames DineOut, || School:
*Significant
xtmixed logIL6_P3 i.sleepdeptsevere sex_n AgeP3 nap_bi_n
i.medical_history_n vediogames DineOut, || School:
*Significant

xtmixed logIL6_P3 i.sleepdeptcat sex_n AgeP3 nap_bi_n
i.medical_history_n vediogames DineOut, || School:
xtmixed logIL6_P3 i.sleepdeptcat sex_n AgeP3 nap_bi_n
i.medical_history_n vediogames BMI3 DineOut, || School:

xtmixed logIL6_P3 i.sleepdeptsevere sex_n AgeP3 nap_bi_n
i.medical_history_n vediogames DineOut, || School:
xtmixed logIL6_P3 i.sleepdeptsevere sex_n AgeP3 nap_bi_n
i.medical_history_n vediogames BMI3 DineOut, || School:

xtmixed logIL6_P3 i.socialgetlag_bi sex_n AgeP3 nap_bi_n
i.medical_history_n vediogames DineOut, || School:
xtmixed logIL6_P3 i.socialgetlag_bi sex_n AgeP3 nap_bi_n
i.medical_history_n vediogames BMI3 DineOut, || School:

xtmixed logIL6_P3 i.SJL_bi sex_n AgeP3 nap_bi_n i.medical_history_n
vediogames DineOut, || School:


xtmixed logIL6_P3 i.timeandduration sex_n AgeP3 nap_bi_n
i.medical_history_n vediogames DineOut, || School:
xtmixed logIL6_P3 sleepdept sex_n AgeP3 nap_bi_n i.medical_history_n
vediogames DineOut, || School:
xtmixed logIL6_P3 social_getlag sex_n AgeP3 nap_bi_n
i.medical_history_n vediogames DineOut, || School:
xtmixed logIL6_P3 social_getlag sex_n AgeP3 nap_bi_n
i.medical_history_n vediogames DineOut, || School:
*Significant

xtmixed logIL6_P3 i.durtime sex_n AgeP3 nap_bi_n i.medical_history_n
vediogames DineOut, || School:


*Second: IL6 Serum
gen logIL6_Se_P3 = log (IL6_Se_P3)

xtmixed logIL6_Se_P3 AverageSleepDuration sex_n AgeP3 nap_bi_n
i.medical_history_n vediogames DineOut, || School:
```

```
xtmixed logIL6_Se_P3 Sleep_time_weekday sex_n AgeP3 nap_bi_n
i.medical_history_n vediogames DineOut, || School:
xtmixed logIL6_Se_P3 Sleep_time_weekend_n sex_n AgeP3 nap_bi_n
i.medical_history_n vediogames DineOut, || School:

xtmixed logIL6_Se_P3 bedtime_both2 sex_n AgeP3 nap_bi_n
i.medical_history_n vediogames DineOut, || School:
xtmixed logIL6_Se_P3 bed_hour sex_n AgeP3 nap_bi_n i.medical_history_n
vediogames DineOut, || School:
xtmixed logIL6_Se_P3 bed_weekend sex_n AgeP3 nap_bi_n
i.medical_history_n vediogames DineOut, || School:

xtmixed logIL6_Se_P3 i.bedtimecat2 sex_n AgeP3 nap_bi_n
i.medical_history_n vediogames DineOut, || School:

xtmixed logIL6_Se_P3 i.sleepdeptcat sex_n AgeP3 nap_bi_n
i.medical_history_n vediogames DineOut, || School:
xtmixed logIL6_Se_P3 i.sleepdeptsevere sex_n AgeP3 nap_bi_n
i.medical_history_n vediogames DineOut, || School:

xtmixed logIL6_Se_P3 i.socialgetlag_bi sex_n AgeP3 nap_bi_n
i.medical_history_n vediogames DineOut, || School:
xtmixed logIL6_Se_P3 social_getlag sex_n AgeP3 nap_bi_n
i.medical_history_n vediogames DineOut, || School:

xtmixed logIL6_Se_P3 i.durtime sex_n AgeP3 nap_bi_n
i.medical_history_n vediogames DineOut, || School:

xtmixed logIL6_Se_P3 i.bedtimeWDcat2 sex_n AgeP3 nap_bi_n
i.medical_history_n vediogames DineOut, || School:
xtmixed logIL6_Se_P3 i.bedtimeWEcat2 sex_n AgeP3 nap_bi_n
i.medical_history_n vediogames DineOut, || School:
xtmixed logIL6_Se_P3 i.bedtimecat2 sex_n AgeP3 nap_bi_n
i.medical_history_n vediogames BMI3 DineOut, || School:
xtmixed logIL6_Se_P3 i.sleepdeptcat sex_n AgeP3 nap_bi_n
i.medical_history_n vediogames BMI3 DineOut, || School:
xtmixed logIL6_Se_P3 i.sleepdeptsevere sex_n AgeP3 nap_bi_n
i.medical_history_n vediogames BMI3 DineOut, || School:
xtmixed logIL6_Se_P3 i.socialgetlag_bi sex_n AgeP3 nap_bi_n
i.medical_history_n vediogames BMI3 DineOut, || School:

***************************


xtmixed IL6_Se_P3 AverageSleepDuration sex_n AgeP3 nap_bi_n
i.medical_history_n vediogames DineOut, || School:
xtmixed IL6_Se_P3 Sleep_time_weekday sex_n AgeP3 nap_bi_n
i.medical_history_n vediogames DineOut, || School:
xtmixed IL6_Se_P3 Sleep_time_weekend_n sex_n AgeP3 nap_bi_n
i.medical_history_n vediogames DineOut, || School:
```

```
xtmixed IL6_Se_P3 bedtime_both2 sex_n AgeP3 nap_bi_n
i.medical_history_n vediogames DineOut, || School:
xtmixed IL6_Se_P3 bed_hour sex_n AgeP3 nap_bi_n i.medical_history_n
vediogames DineOut, || School:
xtmixed IL6_Se_P3 bed_weekend sex_n AgeP3 nap_bi_n i.medical_history_n
vediogames DineOut, || School:
*significant

xtmixed IL6_Se_P3 i.bedtimecat2 sex_n AgeP3 nap_bi_n
i.medical_history_n vediogames DineOut, || School:
*sig
xtmixed IL6_Se_P3 i.bedtimeWDcat2 sex_n AgeP3 nap_bi_n
i.medical_history_n vediogames DineOut, || School:
*sig
xtmixed IL6_Se_P3 i.bedtimeWEcat2 sex_n AgeP3 nap_bi_n
i.medical_history_n vediogames DineOut, || School:

xtmixed IL6_Se_P3 i.socialgetlag_bi sex_n AgeP3 nap_bi_n
i.medical_history_n vediogames DineOut, || School:
xtmixed IL6_Se_P3 i.sleepdeptcat sex_n AgeP3 nap_bi_n
i.medical_history_n vediogames DineOut, || School:
xtmixed IL6_Se_P3 i.sleepdeptsevere sex_n AgeP3 nap_bi_n
i.medical_history_n vediogames DineOut, || School:

xtmixed IL6_Se_P3 bed12new sex_n AgeP3 nap_bi_n i.medical_history_n
vediogames DineOut, || School:

xtmixed IL6_Se_P3 bed12 sex_n AgeP3 nap_bi_n i.medical_history_n
vediogames DineOut, || School:
*Significant
xtmixed IL6_Se_P3 bed12bin_n sex_n AgeP3 nap_bi_n i.medical_history_n
vediogames DineOut, || School:
*Significant
xtmixed IL6_Se_P3 bed12_weekend_bi sex_n AgeP3 nap_bi_n
i.medical_history_n vediogames DineOut, || School:

xtmixed IL6_Se_P3 i.bedtime123 sex_n AgeP3 nap_bi_n
i.medical_history_n vediogames DineOut, || School:
xtmixed IL6_Se_P3 bedtimecat sex_n AgeP3 nap_bi_n i.medical_history_n
vediogames DineOut, || School:
xtmixed IL6_Se_P3 i.bedtimeWDcat sex_n AgeP3 nap_bi_n
i.medical_history_n vediogames DineOut, || School:
*Significant
xtmixed IL6_Se_P3 i.bedtimeWEcat sex_n AgeP3 nap_bi_n
i.medical_history_n vediogames DineOut, || School:

xtmixed IL6_Se_P3 social_getlag sex_n AgeP3 nap_bi_n
i.medical_history_n vediogames DineOut, || School:
xtmixed IL6_Se_P3 i.timeandduration sex_n AgeP3 nap_bi_n
```

```
i.medical_history_n vediogames DineOut, || School:
xtmixed IL6_Se_P3 sleepdept sex_n AgeP3 nap_bi_n i.medical_history_n
vediogames DineOut, || School:



*Fourth: logAdiponectin_P3_n
xtmixed logAdiponectin_P3 AverageSleepDuration sex_n AgeP3 nap_bi_n
i.medical_history_n vediogames DineOut, || School:
xtmixed logAdiponectin_P3 AverageSleepDuration logInsulin_P3 sex_n
AgeP3 nap_bi_n i.medical_history_n vediogames DineOut, || School:
xtmixed logAdiponectin_P3 AverageSleepDuration sex_n AgeP3 nap_bi_n
i.medical_history_n vediogames BMI3 DineOut, || School:

xtmixed logAdiponectin_P3 Sleep_time_weekday_n sex_n AgeP3 nap_bi_n
i.medical_history_n vediogames DineOut, || School:
xtmixed logAdiponectin_P3 Sleep_time_weekend_n sex_n AgeP3 nap_bi_n
i.medical_history_n vediogames DineOut, || School:

xtmixed logAdiponectin_P3 bedtime_both2 sex_n AgeP3 nap_bi_n
i.medical_history_n vediogames DineOut, || School:
xtmixed logAdiponectin_P3 bed_hour sex_n AgeP3 nap_bi_n
i.medical_history_n vediogames DineOut, || School:
xtmixed logAdiponectin_P3 bed_weekend sex_n AgeP3 nap_bi_n
i.medical_history_n vediogames DineOut, || School:

xtmixed logAdiponectin_P3 i.bedtimecat2 sex_n AgeP3 nap_bi_n
i.medical_history_n vediogames DineOut, || School:
xtmixed logAdiponectin_P3 i.bedtimecat2 logInsulin_P3 sex_n AgeP3
nap_bi_n i.medical_history_n vediogames DineOut, || School:
xtmixed logAdiponectin_P3 i.bedtimeWDcat2 sex_n AgeP3 nap_bi_n
i.medical_history_n vediogames DineOut, || School:
xtmixed logAdiponectin_P3 i.bedtimeWEcat2 sex_n AgeP3 nap_bi_n
i.medical_history_n vediogames DineOut, || School:

xtmixed logAdiponectin_P3 i.sleepdeptcat sex_n AgeP3 nap_bi_n
i.medical_history_n vediogames DineOut, || School:
xtmixed logAdiponectin_P3 i.sleepdeptsevere sex_n AgeP3 nap_bi_n
i.medical_history_n vediogames DineOut, || School:
xtmixed logAdiponectin_P3 i.socialgetlag_bi sex_n AgeP3 nap_bi_n
i.medical_history_n vediogames DineOut, || School:

xtmixed logAdiponectin_P3 bed12new sex_n AgeP3 nap_bi_n
i.medical_history_n vediogames DineOut, || School:
xtmixed logAdiponectin_P3 bedtimecat sex_n AgeP3 nap_bi_n
i.medical_history_n vediogames DineOut, || School:
xtmixed logAdiponectin_P3 bed12 sex_n AgeP3 nap_bi_n
i.medical_history_n vediogames DineOut, || School:
xtmixed logAdiponectin_P3 bed12bin_n sex_n AgeP3 nap_bi_n
i.medical_history_n vediogames DineOut, || School:
```

```
xtmixed logAdiponectin_P3 bed12_weekend_bi sex_n AgeP3 nap_bi_n
i.medical_history_n vediogames DineOut, || School:

xtmixed logAdiponectin_P3 i.bedtime123 sex_n AgeP3 nap_bi_n
i.medical_history_n vediogames DineOut, || School:
xtmixed logAdiponectin_P3 i.bedtimeWDcat sex_n AgeP3 nap_bi_n
i.medical_history_n vediogames DineOut, || School:
xtmixed logAdiponectin_P3 i.bedtimeWEcat sex_n AgeP3 nap_bi_n
i.medical_history_n vediogames DineOut, || School:

xtmixed logAdiponectin_P3 i.timeandduration sex_n AgeP3 nap_bi_n
i.medical_history_n vediogames DineOut, || School:
xtmixed logAdiponectin_P3 sleepdept sex_n AgeP3 nap_bi_n
i.medical_history_n vediogames DineOut, || School:
xtmixed logAdiponectin_P3 social_getlag sex_n AgeP3 nap_bi_n
i.medical_history_n vediogames DineOut, || School:

xtmixed logAdiponectin_P3 i.durtime sex_n AgeP3 nap_bi_n
i.medical_history_n vediogames DineOut, || School:


*Fifth: logAdiponectin_Se_P3
xtmixed logAdiponectin_Se_P3 AverageSleepDuration sex_n AgeP3 nap_bi_n
i.medical_history_n vediogames DineOut, || School:
xtmixed logAdiponectin_Se_P3 AverageSleepDuration logInsulin_Se_P3
sex_n AgeP3 nap_bi_n i.medical_history_n vediogames DineOut, ||
School:
xtmixed logAdiponectin_Se_P3 AverageSleepDuration sex_n AgeP3 nap_bi_n
i.medical_history_n vediogames BMI3 DineOut, || School:

xtmixed logAdiponectin_Se_P3 Sleep_time_weekday_n sex_n AgeP3 nap_bi_n
i.medical_history_n vediogames DineOut, || School:
xtmixed logAdiponectin_Se_P3 Sleep_time_weekend_n sex_n AgeP3 nap_bi_n
i.medical_history_n vediogames DineOut, || School:

xtmixed logAdiponectin_Se_P3 bedtime_both2 sex_n AgeP3 nap_bi_n
i.medical_history_n vediogames DineOut, || School:
xtmixed logAdiponectin_Se_P3 bed_hour sex_n AgeP3 nap_bi_n
i.medical_history_n vediogames DineOut, || School:
xtmixed logAdiponectin_Se_P3 bed_weekend sex_n AgeP3 nap_bi_n
i.medical_history_n vediogames DineOut, || School:

xtmixed logAdiponectin_Se_P3 i.bedtimecat2 sex_n AgeP3 nap_bi_n
i.medical_history_n vediogames DineOut, || School:
xtmixed logAdiponectin_Se_P3 i.bedtimecat2 logInsulin_Se_P3 sex_n
AgeP3 nap_bi_n i.medical_history_n vediogames DineOut, || School:

xtmixed logAdiponectin_Se_P3 i.bedtimeWDcat2 sex_n AgeP3 nap_bi_n
i.medical_history_n vediogames DineOut, || School:
xtmixed logAdiponectin_Se_P3 i.bedtimeWEcat2 sex_n AgeP3 nap_bi_n
```

```
i.medical_history_n vediogames DineOut, || School:

xtmixed logAdiponectin_Se_P3 i.sleepdeptcat sex_n AgeP3 nap_bi_n
i.medical_history_n vediogames DineOut, || School:
xtmixed logAdiponectin_Se_P3 i.sleepdeptsevere sex_n AgeP3 nap_bi_n
i.medical_history_n vediogames DineOut, || School:
xtmixed logAdiponectin_Se_P3 i.socialgetlag_bi sex_n AgeP3 nap_bi_n
i.medical_history_n vediogames DineOut, || School:

xtmixed logAdiponectin_Se_P3 bed12new sex_n AgeP3 nap_bi_n
i.medical_history_n vediogames DineOut, || School:
xtmixed logAdiponectin_Se_P3 bedtimecat sex_n AgeP3 nap_bi_n
i.medical_history_n vediogames DineOut, || School:

xtmixed logAdiponectin_Se_P3 bed12 sex_n AgeP3 nap_bi_n
i.medical_history_n vediogames DineOut, || School:
xtmixed logAdiponectin_Se_P3 bed12bin_n sex_n AgeP3 nap_bi_n
i.medical_history_n vediogames DineOut, || School:
xtmixed logAdiponectin_Se_P3 bed12_weekend_bi sex_n AgeP3 nap_bi_n
i.medical_history_n vediogames DineOut, || School:

xtmixed logAdiponectin_Se_P3 i.bedtime123 sex_n AgeP3 nap_bi_n
i.medical_history_n vediogames DineOut, || School:
xtmixed logAdiponectin_Se_P3 i.bedtimeWDcat sex_n AgeP3 nap_bi_n
i.medical_history_n vediogames DineOut, || School:
xtmixed logAdiponectin_Se_P3 i.bedtimeWEcat sex_n AgeP3 nap_bi_n
i.medical_history_n vediogames DineOut, || School:

xtmixed logAdiponectin_Se_P3 i.timeandduration sex_n AgeP3 nap_bi_n
i.medical_history_n vediogames DineOut, || School:
xtmixed logAdiponectin_Se_P3 sleepdept sex_n AgeP3 nap_bi_n
i.medical_history_n vediogames DineOut, || School:
xtmixed logAdiponectin_Se_P3 social_getlag sex_n AgeP3 nap_bi_n
i.medical_history_n vediogames DineOut, || School:

xtmixed logAdiponectin_Se_P3 i.durtime sex_n AgeP3 nap_bi_n
i.medical_history_n vediogames DineOut, || School:


*Sixth: logIL10
xtmixed logIL10_P3 AverageSleepDuration sex_n AgeP3 nap_bi_n
i.medical_history_n vediogames DineOut, || School:
xtmixed logIL10_P3 Sleep_time_weekday_n sex_n AgeP3 nap_bi_n
i.medical_history_n vediogames DineOut, || School:
xtmixed logIL10_P3 Sleep_time_weekend_n sex_n AgeP3 nap_bi_n
i.medical_history_n vediogames DineOut, || School:

xtmixed logIL10_P3 bedtime_both2 sex_n AgeP3 nap_bi_n
i.medical_history_n vediogames DineOut, || School:
xtmixed logIL10_P3 bed_hour sex_n AgeP3 nap_bi_n i.medical_history_n
```

```
vediogames DineOut, || School:
xtmixed logIL10_P3 bed_weekend sex_n AgeP3 nap_bi_n
i.medical_history_n vediogames DineOut, || School:

xtmixed logIL10_P3 i.bedtimecat2 sex_n AgeP3 nap_bi_n
i.medical_history_n vediogames DineOut, || School:

xtmixed logIL10_P3 i.sleepdeptcat sex_n AgeP3 nap_bi_n
i.medical_history_n vediogames DineOut, || School:
xtmixed logIL10_P3 i.sleepdeptsevere sex_n AgeP3 nap_bi_n
i.medical_history_n vediogames DineOut, || School:

xtmixed logIL10_P3 i.socialgetlag_bi sex_n AgeP3 nap_bi_n
i.medical_history_n vediogames DineOut, || School:
xtmixed logIL10_P3 social_getlag sex_n AgeP3 nap_bi_n
i.medical_history_n vediogames DineOut, || School:

xtmixed logIL10_P3 i.durtime sex_n AgeP3 nap_bi_n i.medical_history_n
vediogames DineOut, || School:

xtmixed logIL10_P3 i.bedtimeWDcat2 sex_n AgeP3 nap_bi_n
i.medical_history_n vediogames DineOut, || School:
xtmixed logIL10_P3 i.bedtimeWEcat2 sex_n AgeP3 nap_bi_n
i.medical_history_n vediogames DineOut, || School:


************



*Seventh: logIL10_Se
xtmixed logIL10_Se AverageSleepDuration sex_n AgeP3 nap_bi_n
i.medical_history_n vediogames DineOut, || School:
xtmixed logIL10_Se Sleep_time_weekday_n sex_n AgeP3 nap_bi_n
i.medical_history_n vediogames DineOut, || School:
xtmixed logIL10_Se Sleep_time_weekend_n sex_n AgeP3 nap_bi_n
i.medical_history_n vediogames DineOut, || School:

xtmixed logIL10_Se bedtime_both2 sex_n AgeP3 nap_bi_n
i.medical_history_n vediogames DineOut, || School:
xtmixed logIL10_Se bed_hour sex_n AgeP3 nap_bi_n i.medical_history_n
vediogames DineOut, || School:
xtmixed logIL10_Se bed_weekend sex_n AgeP3 nap_bi_n
i.medical_history_n vediogames DineOut, || School:

xtmixed logIL10_Se i.bedtimecat2 sex_n AgeP3 nap_bi_n
i.medical_history_n vediogames DineOut, || School:

xtmixed logIL10_Se i.durtime sex_n AgeP3 nap_bi_n i.medical_history_n
vediogames DineOut, || School:
```

```
xtmixed logIL10_Se i.sleepdeptcat sex_n AgeP3 nap_bi_n
i.medical_history_n vediogames DineOut, || School:
xtmixed logIL10_Se i.sleepdeptsevere sex_n AgeP3 nap_bi_n
i.medical_history_n vediogames DineOut, || School:

xtmixed logIL10_Se i.socialgetlag_bi sex_n AgeP3 nap_bi_n
i.medical_history_n vediogames DineOut, || School:
xtmixed logIL10_Se social_getlag sex_n AgeP3 nap_bi_n
i.medical_history_n vediogames DineOut, || School:

xtmixed logIL10_Se i.durtime sex_n AgeP3 nap_bi_n i.medical_history_n
vediogames DineOut, || School:


xtmixed logIL10_Se bed12new sex_n AgeP3 nap_bi_n i.medical_history_n
vediogames DineOut, || School:
*Significant

xtmixed logIL10_Se bedtimecat sex_n AgeP3 nap_bi_n i.medical_history_n
vediogames DineOut, || School:

xtmixed logIL10_Se bed12 sex_n AgeP3 nap_bi_n i.medical_history_n
vediogames DineOut, || School:
xtmixed logIL10_Se bed12bin_n sex_n AgeP3 nap_bi_n i.medical_history_n
vediogames DineOut, || School:
xtmixed logIL10_Se bed12_weekend_bi sex_n AgeP3 nap_bi_n
i.medical_history_n vediogames DineOut, || School:

xtmixed logIL10_Se i.bedtime123 sex_n AgeP3 nap_bi_n
i.medical_history_n vediogames DineOut, || School:
*Significant
xtmixed logIL10_Se i.bedtimeWDcat sex_n AgeP3 nap_bi_n
i.medical_history_n vediogames DineOut, || School:
xtmixed logIL10_Se i.bedtimeWEcat sex_n AgeP3 nap_bi_n
i.medical_history_n vediogames DineOut, || School:

xtmixed logIL10_Se i.timeandduration sex_n AgeP3 nap_bi_n
i.medical_history_n vediogames DineOut, || School:
xtmixed logIL10_Se sleepdept sex_n AgeP3 nap_bi_n i.medical_history_n
vediogames DineOut, || School:
xtmixed logIL10_Se social_getlag sex_n AgeP3 nap_bi_n
i.medical_history_n vediogames DineOut, || School:
*Significant

xtmixed logIL10_Se i.bedtimeWDcat2 sex_n AgeP3 nap_bi_n
i.medical_history_n vediogames DineOut, || School:
xtmixed logIL10_Se i.bedtimeWEcat2 sex_n AgeP3 nap_bi_n
i.medical_history_n vediogames DineOut, || School:
xtmixed logIL10_Se i.bedtimecat2 sex_n AgeP3 nap_bi_n
```

```
i.medical_history_n vediogames BMI3 DineOut, || School:

*Eight: logIL8_P3
xtmixed logIL8_P3 AverageSleepDuration sex_n AgeP3 nap_bi_n
i.medical_history_n vediogames DineOut, || School:
xtmixed logIL8_P3 Sleep_time_weekday_n sex_n AgeP3 nap_bi_n
i.medical_history_n vediogames DineOut, || School:
xtmixed logIL8_P3 Sleep_time_weekend_n sex_n AgeP3 nap_bi_n
i.medical_history_n vediogames DineOut, || School:

xtmixed logIL8_P3 bedtime_both2 sex_n AgeP3 nap_bi_n
i.medical_history_n vediogames DineOut, || School:
xtmixed logIL8_P3 bed_hour sex_n AgeP3 nap_bi_n i.medical_history_n
vediogames DineOut, || School:
xtmixed logIL8_P3 bed_weekend sex_n AgeP3 nap_bi_n i.medical_history_n
vediogames DineOut, || School:

xtmixed logIL8_P3 i.bedtimecat2 sex_n AgeP3 nap_bi_n
i.medical_history_n vediogames DineOut, || School:

xtmixed logIL8_P3 i.sleepdeptcat sex_n AgeP3 nap_bi_n
i.medical_history_n vediogames DineOut, || School:
xtmixed logIL8_P3 i.sleepdeptsevere sex_n AgeP3 nap_bi_n
i.medical_history_n vediogames DineOut, || School:

xtmixed logIL8_P3 i.socialgetlag_bi sex_n AgeP3 nap_bi_n
i.medical_history_n vediogames DineOut, || School:
xtmixed logIL8_P3 social_getlag sex_n AgeP3 nap_bi_n
i.medical_history_n vediogames DineOut, || School:

xtmixed logIL8_P3 i.durtime sex_n AgeP3 nap_bi_n i.medical_history_n
vediogames DineOut, || School:


xtmixed logIL8_P3 i.bedtimecat2 sex_n AgeP3 nap_bi_n
i.medical_history_n vediogames BMI3 DineOut, || School:
xtmixed logIL8_P3 bed12new sex_n AgeP3 nap_bi_n i.medical_history_n
vediogames DineOut, || School:
xtmixed logIL8_P3 bedtimecat sex_n AgeP3 nap_bi_n i.medical_history_n
vediogames DineOut, || School:

xtmixed logIL8_P3 bed12 sex_n AgeP3 nap_bi_n i.medical_history_n
vediogames DineOut, || School:
xtmixed logIL8_P3 bed12bin_n sex_n AgeP3 nap_bi_n i.medical_history_n
vediogames DineOut, || School:
xtmixed logIL8_P3 bed12_weekend_bi sex_n AgeP3 nap_bi_n
i.medical_history_n vediogames DineOut, || School:

xtmixed logIL8_P3 i.bedtime123 sex_n AgeP3 nap_bi_n
i.medical_history_n vediogames DineOut, || School:
```

```
xtmixed logIL8_P3 i.bedtimeWDcat sex_n AgeP3 nap_bi_n
i.medical_history_n vediogames DineOut, || School:
xtmixed logIL8_P3 i.bedtimeWEcat sex_n AgeP3 nap_bi_n
i.medical_history_n vediogames DineOut, || School:

xtmixed logIL8_P3 i.timeandduration sex_n AgeP3 nap_bi_n
i.medical_history_n vediogames DineOut, || School:
xtmixed logIL8_P3 sleepdept sex_n AgeP3 nap_bi_n i.medical_history_n
vediogames DineOut, || School:
xtmixed logIL8_P3 social_getlag sex_n AgeP3 nap_bi_n
i.medical_history_n vediogames DineOut, || School:

xtmixed logIL8_P3 i.bedtimeWDcat2 sex_n AgeP3 nap_bi_n
i.medical_history_n vediogames DineOut, || School:
xtmixed logIL8_P3 i.bedtimeWEcat2 sex_n AgeP3 nap_bi_n
i.medical_history_n vediogames DineOut, || School:

*Ninth: logIL8_Se_P3
xtmixed logIL8_Se_P3 AverageSleepDuration sex_n AgeP3 nap_bi_n
i.medical_history_n vediogames DineOut, || School:
xtmixed logIL8_Se_P3 Sleep_time_weekday_n sex_n AgeP3 nap_bi_n
i.medical_history_n vediogames DineOut, || School:
xtmixed logIL8_Se_P3 Sleep_time_weekend_n sex_n AgeP3 nap_bi_n
i.medical_history_n vediogames DineOut, || School:

xtmixed logIL8_Se_P3 bedtime_both2 sex_n AgeP3 nap_bi_n
i.medical_history_n vediogames DineOut, || School:
*Significant
xtmixed logIL8_Se_P3 bed_hour sex_n AgeP3 nap_bi_n i.medical_history_n
vediogames DineOut, || School:
*Significant
xtmixed logIL8_Se_P3 bed_weekend sex_n AgeP3 nap_bi_n
i.medical_history_n vediogames DineOut, || School:

xtmixed logIL8_Se_P3 i.bedtimecat2 sex_n AgeP3 nap_bi_n
i.medical_history_n vediogames DineOut, || School:
*sig
xtmixed logIL8_Se_P3 i.bedtimeWDcat2 sex_n AgeP3 nap_bi_n
i.medical_history_n vediogames DineOut, || School:
*sig
xtmixed logIL8_Se_P3 i.bedtimeWEcat2 sex_n AgeP3 nap_bi_n
i.medical_history_n vediogames DineOut, || School:
*sig

xtmixed logIL8_Se_P3 i.bedtimecat2 sex_n AgeP3 nap_bi_n
i.medical_history_n vediogames BMI3 DineOut, || School:

xtmixed logIL8_Se_P3 i.sleepdeptcat sex_n AgeP3 nap_bi_n
i.medical_history_n vediogames DineOut, || School:
*Significant
```

```
xtmixed logIL8_Se_P3 i.sleepdeptsevere sex_n AgeP3 nap_bi_n
i.medical_history_n vediogames DineOut, || School:
xtmixed logIL8_Se_P3 i.socialgetlag_bi sex_n AgeP3 nap_bi_n
i.medical_history_n vediogames DineOut, || School:

xtmixed logIL8_Se_P3 social_getlag sex_n AgeP3 nap_bi_n
i.medical_history_n vediogames DineOut, || School:

xtmixed logIL8_Se_P3 bed12new sex_n AgeP3 nap_bi_n i.medical_history_n
vediogames DineOut, || School:

xtmixed logIL8_Se_P3 bedtimecat sex_n AgeP3 nap_bi_n
i.medical_history_n vediogames DineOut, || School:
*sig

xtmixed logIL8_Se_P3 bed12 sex_n AgeP3 nap_bi_n i.medical_history_n
vediogames DineOut, || School:
*Significant
xtmixed logIL8_Se_P3 bed12bin_n sex_n AgeP3 nap_bi_n
i.medical_history_n vediogames DineOut, || School:
*Significant
xtmixed logIL8_Se_P3 bed12_weekend_bi sex_n AgeP3 nap_bi_n
i.medical_history_n vediogames DineOut, || School:
*Significant

xtmixed logIL8_Se_P3 i.bedtime123 sex_n AgeP3 nap_bi_n
i.medical_history_n vediogames DineOut, || School:
*Marginal significance
xtmixed logIL8_Se_P3 i.bedtimeWDcat sex_n AgeP3 nap_bi_n
i.medical_history_n vediogames DineOut, || School:
*Significant

xtmixed logIL8_Se_P3 i.bedtimeWEcat sex_n AgeP3 nap_bi_n
i.medical_history_n vediogames DineOut, || School:
*Significant

xtmixed logIL8_Se_P3 i.timeandduration sex_n AgeP3 nap_bi_n
i.medical_history_n vediogames DineOut, || School:
xtmixed logIL8_Se_P3 sleepdept sex_n AgeP3 nap_bi_n
i.medical_history_n vediogames DineOut, || School:
xtmixed logIL8_Se_P3 social_getlag sex_n AgeP3 nap_bi_n
i.medical_history_n vediogames DineOut, || School:

xtmixed logIL8_Se_P3 i.durtime sex_n AgeP3 nap_bi_n
i.medical_history_n vediogames DineOut, || School:


*Tenth: logLeptin_P3
xtmixed logLeptin_P3 AverageSleepDuration sex_n AgeP3 nap_bi_n
i.medical_history_n vediogames DineOut, || School:
```

```
xtmixed logLeptin_P3 AverageSleepDuration logInsulin_P3 sex_n AgeP3
nap_bi_n i.medical_history_n vediogames DineOut, || School:
xtmixed logLeptin_P3 AverageSleepDuration sex_n AgeP3 nap_bi_n
i.medical_history_n vediogames BMI3 DineOut, || School:

xtmixed logLeptin_P3 Sleep_time_weekday_n sex_n AgeP3 nap_bi_n
i.medical_history_n vediogames DineOut, || School:
xtmixed logLeptin_P3 Sleep_time_weekend_n sex_n AgeP3 nap_bi_n
i.medical_history_n vediogames DineOut, || School:

xtmixed logLeptin_P3 bedtime_both2 sex_n AgeP3 nap_bi_n
i.medical_history_n vediogames DineOut, || School:
xtmixed logLeptin_P3 bed_hour sex_n AgeP3 nap_bi_n i.medical_history_n
vediogames DineOut, || School:
xtmixed logLeptin_P3 bed_weekend sex_n AgeP3 nap_bi_n
i.medical_history_n vediogames DineOut, || School:

xtmixed logLeptin_P3 i.bedtimecat2 sex_n AgeP3 nap_bi_n
i.medical_history_n vediogames DineOut, || School:
xtmixed logLeptin_P3 i.bedtimecat2 logInsulin_P3 sex_n AgeP3 nap_bi_n
i.medical_history_n vediogames DineOut, || School:

xtmixed logLeptin_P3 i.bedtimeWDcat2 sex_n AgeP3 nap_bi_n
i.medical_history_n vediogames DineOut, || School:
xtmixed logLeptin_P3 i.bedtimeWEcat2 sex_n AgeP3 nap_bi_n
i.medical_history_n vediogames DineOut, || School:

xtmixed logLeptin_P3 i.bedtimecat2 sex_n AgeP3 nap_bi_n
i.medical_history_n vediogames BMI3 DineOut, || School:

xtmixed logLeptin_P3 i.sleepdeptcat sex_n AgeP3 nap_bi_n
i.medical_history_n vediogames DineOut, || School:
xtmixed logLeptin_P3 i.sleepdeptsevere sex_n AgeP3 nap_bi_n
i.medical_history_n vediogames DineOut, || School:

xtmixed logLeptin_P3 i.socialgetlag_bi sex_n AgeP3 nap_bi_n
i.medical_history_n vediogames DineOut, || School:
xtmixed logLeptin_P3 i.socialgetlag_bi sex_n AgeP3 nap_bi_n
i.medical_history_n vediogames BMI3 DineOut, || School:

xtmixed logLeptin_P3 i.SJL_bi sex_n AgeP3 nap_bi_n i.medical_history_n
vediogames DineOut, || School:

xtmixed logLeptin_P3 bed12new sex_n AgeP3 nap_bi_n i.medical_history_n
vediogames DineOut, || School:
xtmixed logLeptin_P3 bedtimecat sex_n AgeP3 nap_bi_n
i.medical_history_n vediogames DineOut, || School:

xtmixed logLeptin_P3 bed12 sex_n AgeP3 nap_bi_n i.medical_history_n
vediogames DineOut, || School:
```

```
xtmixed logLeptin_P3 bed12bin_n sex_n AgeP3 nap_bi_n
i.medical_history_n vediogames DineOut, || School:
xtmixed logLeptin_P3 bed12_weekend_bi sex_n AgeP3 nap_bi_n
i.medical_history_n vediogames DineOut, || School:

xtmixed logLeptin_P3 i.bedtime123 sex_n AgeP3 nap_bi_n
i.medical_history_n vediogames DineOut, || School:
xtmixed logLeptin_P3 i.bedtimeWDcat sex_n AgeP3 nap_bi_n
i.medical_history_n vediogames DineOut, || School:
xtmixed logLeptin_P3 i.bedtimeWEcat sex_n AgeP3 nap_bi_n
i.medical_history_n vediogames DineOut, || School:

xtmixed logLeptin_P3 i.timeandduration sex_n AgeP3 nap_bi_n
i.medical_history_n vediogames DineOut, || School:
xtmixed logLeptin_P3 sleepdept sex_n AgeP3 nap_bi_n
i.medical_history_n vediogames DineOut, || School:
xtmixed logLeptin_P3 social_getlag sex_n AgeP3 nap_bi_n
i.medical_history_n vediogames DineOut, || School:

xtmixed logLeptin_P3 i.durtime sex_n AgeP3 nap_bi_n
i.medical_history_n vediogames DineOut, || School:


*Eleventh: logLeptin_Se_P3
xtmixed logLeptin_Se_P3 AverageSleepDuration sex_n AgeP3 nap_bi_n
i.medical_history_n vediogames DineOut, || School:
xtmixed logLeptin_Se_P3 AverageSleepDuration logInsulin_Se_P3 sex_n
AgeP3 nap_bi_n i.medical_history_n vediogames DineOut, || School:
xtmixed logLeptin_Se_P3 AverageSleepDuration sex_n AgeP3 nap_bi_n
i.medical_history_n vediogames BMI3 DineOut, || School:

xtmixed logLeptin_Se_P3 Sleep_time_weekday_n sex_n AgeP3 nap_bi_n
i.medical_history_n vediogames DineOut, || School:
xtmixed logLeptin_Se_P3 Sleep_time_weekday_n logInsulin_Se_P3 sex_n
AgeP3 nap_bi_n i.medical_history_n vediogames DineOut, || School:

xtmixed logLeptin_Se_P3 Sleep_time_weekend_n sex_n AgeP3 nap_bi_n
i.medical_history_n vediogames DineOut, || School:

xtmixed logLeptin_Se_P3 bedtime_both2 sex_n AgeP3 nap_bi_n
i.medical_history_n vediogames DineOut, || School:
xtmixed logLeptin_Se_P3 bed_hour sex_n AgeP3 nap_bi_n
i.medical_history_n vediogames DineOut, || School:
xtmixed logLeptin_Se_P3 bed_weekend sex_n AgeP3 nap_bi_n
i.medical_history_n vediogames DineOut, || School:

xtmixed logLeptin_Se_P3 i.bedtimecat2 sex_n AgeP3 nap_bi_n
i.medical_history_n vediogames DineOut, || School:
xtmixed logLeptin_Se_P3 i.bedtimecat2 logInsulin_Se_P3 sex_n AgeP3
nap_bi_n i.medical_history_n vediogames DineOut, || School:
```

```
xtmixed logLeptin_Se_P3 i.bedtimeWDcat2 sex_n AgeP3 nap_bi_n
i.medical_history_n vediogames DineOut, || School:
*sig
xtmixed logLeptin_Se_P3 i.bedtimeWEcat2 sex_n AgeP3 nap_bi_n
i.medical_history_n vediogames DineOut, || School:

xtmixed logLeptin_Se_P3 i.bedtimecat2 sex_n AgeP3 nap_bi_n
i.medical_history_n vediogames BMI3 DineOut, || School:

xtmixed logLeptin_Se_P3 i.sleepdeptcat sex_n AgeP3 nap_bi_n
i.medical_history_n vediogames DineOut, || School:
xtmixed logLeptin_Se_P3 i.sleepdeptsevere sex_n AgeP3 nap_bi_n
i.medical_history_n vediogames DineOut, || School:
xtmixed logLeptin_Se_P3 i.socialgetlag_bi sex_n AgeP3 nap_bi_n
i.medical_history_n vediogames DineOut, || School:
xtmixed logLeptin_Se_P3 i.SJL_bi sex_n AgeP3 nap_bi_n
i.medical_history_n vediogames DineOut, || School:


xtmixed logLeptin_Se_P3 bed12new sex_n AgeP3 nap_bi_n
i.medical_history_n vediogames DineOut, || School:
*marginal significance
xtmixed logLeptin_Se_P3 bedtimecat sex_n AgeP3 nap_bi_n
i.medical_history_n vediogames DineOut, || School:
*sig

xtmixed logLeptin_Se_P3 bed12 sex_n AgeP3 nap_bi_n i.medical_history_n
vediogames DineOut, || School:
xtmixed logLeptin_Se_P3 bed12bin_n sex_n AgeP3 nap_bi_n
i.medical_history_n vediogames DineOut, || School:
xtmixed logLeptin_Se_P3 bed12_weekend_bi sex_n AgeP3 nap_bi_n
i.medical_history_n vediogames DineOut, || School:

xtmixed logLeptin_Se_P3 i.bedtime123 sex_n AgeP3 nap_bi_n
i.medical_history_n vediogames DineOut, || School:
xtmixed logLeptin_Se_P3 i.bedtimeWDcat sex_n AgeP3 nap_bi_n
i.medical_history_n vediogames DineOut, || School:
xtmixed logLeptin_Se_P3 i.bedtimeWEcat sex_n AgeP3 nap_bi_n
i.medical_history_n vediogames DineOut, || School:

xtmixed logLeptin_Se_P3 i.timeandduration sex_n AgeP3 nap_bi_n
i.medical_history_n vediogames DineOut, || School:
xtmixed logLeptin_Se_P3 sleepdept sex_n AgeP3 nap_bi_n
i.medical_history_n vediogames DineOut, || School:
xtmixed logLeptin_Se_P3 social_getlag sex_n AgeP3 nap_bi_n
i.medical_history_n vediogames DineOut, || School:

xtmixed logLeptin_Se_P3 logsocial_getlag sex_n AgeP3 nap_bi_n
i.medical_history_n vediogames DineOut, || School:
```

```
xtmixed logLeptin_Se_P3 i.durtime sex_n AgeP3 nap_bi_n
i.medical_history_n vediogames DineOut, || School:


*Twelveth: VEGF_P3
xtmixed VEGF_P3 AverageSleepDuration sex_n AgeP3 nap_bi_n
i.medical_history_n vediogames DineOut, || School:
xtmixed VEGF_P3 Sleep_time_weekday_n sex_n AgeP3 nap_bi_n
i.medical_history_n vediogames DineOut, || School:
xtmixed VEGF_P3 Sleep_time_weekend_n sex_n AgeP3 nap_bi_n
i.medical_history_n vediogames DineOut, || School:

xtmixed VEGF_P3 bedtime_both2 sex_n AgeP3 nap_bi_n i.medical_history_n
vediogames DineOut, || School:
xtmixed VEGF_P3 bed_hour sex_n AgeP3 nap_bi_n i.medical_history_n
vediogames DineOut, || School:
xtmixed VEGF_P3 bed_weekend sex_n AgeP3 nap_bi_n i.medical_history_n
vediogames DineOut, || School:

xtmixed VEGF_P3 i.bedtimecat2 sex_n AgeP3 nap_bi_n i.medical_history_n
vediogames DineOut, || School:
xtmixed VEGF_P3 i.bedtimeWDcat2 sex_n AgeP3 nap_bi_n
i.medical_history_n vediogames DineOut, || School:
xtmixed VEGF_P3 i.bedtimeWEcat2 sex_n AgeP3 nap_bi_n
i.medical_history_n vediogames DineOut, || School:

xtmixed VEGF_P3 i.sleepdeptcat sex_n AgeP3 nap_bi_n
i.medical_history_n vediogames DineOut, || School:
xtmixed VEGF_P3 i.sleepdeptsevere sex_n AgeP3 nap_bi_n
i.medical_history_n vediogames DineOut, || School:
xtmixed VEGF_P3 i.socialgetlag_bi sex_n AgeP3 nap_bi_n
i.medical_history_n vediogames DineOut, || School:

xtmixed VEGF_P3 bed12new sex_n AgeP3 nap_bi_n i.medical_history_n
vediogames DineOut, || School:
xtmixed VEGF_P3 bedtimecat sex_n AgeP3 nap_bi_n i.medical_history_n
vediogames DineOut, || School:

xtmixed VEGF_P3 bed12 sex_n AgeP3 nap_bi_n i.medical_history_n
vediogames DineOut, || School:
xtmixed VEGF_P3 bed12bin_n sex_n AgeP3 nap_bi_n i.medical_history_n
vediogames DineOut, || School:
xtmixed VEGF_P3 bed12_weekend_bi sex_n AgeP3 nap_bi_n
i.medical_history_n vediogames DineOut, || School:

xtmixed VEGF_P3 i.bedtime123 sex_n AgeP3 nap_bi_n i.medical_history_n
vediogames DineOut, || School:
xtmixed VEGF_P3 i.bedtimeWDcat sex_n AgeP3 nap_bi_n
i.medical_history_n vediogames DineOut, || School:
```

```
xtmixed VEGF_P3 i.bedtimeWEcat sex_n AgeP3 nap_bi_n
i.medical_history_n vediogames DineOut, || School:

xtmixed VEGF_P3 i.timeandduration sex_n AgeP3 nap_bi_n
i.medical_history_n vediogames DineOut, || School:
xtmixed VEGF_P3 sleepdept sex_n AgeP3 nap_bi_n i.medical_history_n
vediogames DineOut, || School:
xtmixed VEGF_P3 social_getlag sex_n AgeP3 nap_bi_n i.medical_history_n
vediogames DineOut, || School:
xtmixed VEGF_P3 social_getlag sex_n AgeP3 nap_bi_n i.medical_history_n
vediogames BMI3 DineOut, || School:

xtmixed VEGF_P3 i.durtime sex_n AgeP3 nap_bi_n i.medical_history_n
vediogames DineOut, || School:


*Thirneeth: logVEGF_Se_P3
xtmixed logVEGF_Se_P3 AverageSleepDuration sex_n AgeP3 nap_bi_n
i.medical_history_n vediogames DineOut, || School:
xtmixed logVEGF_Se_P3 Sleep_time_weekday_n sex_n AgeP3 nap_bi_n
i.medical_history_n vediogames DineOut, || School:
xtmixed logVEGF_Se_P3 Sleep_time_weekend_n sex_n AgeP3 nap_bi_n
i.medical_history_n vediogames DineOut, || School:

xtmixed logVEGF_Se_P3 bedtime_both2 sex_n AgeP3 nap_bi_n
i.medical_history_n vediogames DineOut, || School:
xtmixed logVEGF_Se_P3 bed_hour sex_n AgeP3 nap_bi_n
i.medical_history_n vediogames DineOut, || School:
xtmixed logVEGF_Se_P3 bed_weekend sex_n AgeP3 nap_bi_n
i.medical_history_n vediogames DineOut, || School:

xtmixed logVEGF_Se_P3 i.bedtimecat2 sex_n AgeP3 nap_bi_n
i.medical_history_n vediogames DineOut, || School:
xtmixed logVEGF_Se_P3 i.bedtimeWDcat2 sex_n AgeP3 nap_bi_n
i.medical_history_n vediogames DineOut, || School:
xtmixed logVEGF_Se_P3 i.bedtimeWEcat2 sex_n AgeP3 nap_bi_n
i.medical_history_n vediogames DineOut, || School:

xtmixed logVEGF_Se_P3 i.sleepdeptcat sex_n AgeP3 nap_bi_n
i.medical_history_n vediogames DineOut, || School:
xtmixed logVEGF_Se_P3 i.sleepdeptsevere sex_n AgeP3 nap_bi_n
i.medical_history_n vediogames DineOut, || School:
xtmixed logVEGF_Se_P3 i.socialgetlag_bi sex_n AgeP3 nap_bi_n
i.medical_history_n vediogames DineOut, || School:

xtmixed logVEGF_Se_P3 bed12new sex_n AgeP3 nap_bi_n
i.medical_history_n vediogames DineOut, || School:\
xtmixed logVEGF_Se_P3 bedtimecat sex_n AgeP3 nap_bi_n
i.medical_history_n vediogames DineOut, || School:
```

```
xtmixed logVEGF_Se_P3 bed12 sex_n AgeP3 nap_bi_n i.medical_history_n
vediogames DineOut, || School:
xtmixed logVEGF_Se_P3 bed12bin_n sex_n AgeP3 nap_bi_n
i.medical_history_n vediogames DineOut, || School:
xtmixed logVEGF_Se_P3 bed12_weekend_bi sex_n AgeP3 nap_bi_n
i.medical_history_n vediogames DineOut, || School:

xtmixed logVEGF_Se_P3 i.bedtime123 sex_n AgeP3 nap_bi_n
i.medical_history_n vediogames DineOut, || School:
xtmixed logVEGF_Se_P3 i.bedtimeWDcat sex_n AgeP3 nap_bi_n
i.medical_history_n vediogames DineOut, || School:
xtmixed logVEGF_Se_P3 i.bedtimeWEcat sex_n AgeP3 nap_bi_n
i.medical_history_n vediogames DineOut, || School:

xtmixed logVEGF_Se_P3 i.timeandduration sex_n AgeP3 nap_bi_n
i.medical_history_n vediogames DineOut, || School:
xtmixed logVEGF_Se_P3 sleepdept sex_n AgeP3 nap_bi_n
i.medical_history_n vediogames DineOut, || School:
xtmixed logVEGF_Se_P3 social_getlag sex_n AgeP3 nap_bi_n
i.medical_history_n vediogames DineOut, || School:

xtmixed logVEGF_Se_P3 i.durtime sex_n AgeP3 nap_bi_n
i.medical_history_n vediogames DineOut, || School:


*Fourteenth: logInsulin_P3
*MIXED EFFECT MODEL:
xtmixed logInsulin_P3 AverageSleepDuration sex_n AgeP3 nap_bi_n
i.medical_history_n vediogames DineOut, || School:
xtmixed logInsulin_P3 AverageSleepDuration sex_n AgeP3 nap_bi_n
i.medical_history_n vediogames BMI3 DineOut, || School:

xtmixed logInsulin_P3 Sleep_time_weekday_n sex_n AgeP3 nap_bi_n
i.medical_history_n vediogames DineOut, || School:
xtmixed logInsulin_P3 Sleep_time_weekend_n sex_n AgeP3 nap_bi_n
i.medical_history_n vediogames DineOut, || School:

xtmixed logInsulin_P3 bedtime_both2 sex_n AgeP3 nap_bi_n
i.medical_history_n vediogames DineOut, || School:
xtmixed logInsulin_P3 bed_hour sex_n AgeP3 nap_bi_n
i.medical_history_n vediogames DineOut, || School:
xtmixed logInsulin_P3 bed_weekend sex_n AgeP3 nap_bi_n
i.medical_history_n vediogames DineOut, || School:

xtmixed logInsulin_P3 i.bedtimecat2 sex_n AgeP3 nap_bi_n
i.medical_history_n vediogames DineOut, || School:
xtmixed logInsulin_P3 i.bedtimeWDcat2 sex_n AgeP3 nap_bi_n
i.medical_history_n vediogames DineOut, || School:
xtmixed logInsulin_P3 i.bedtimeWEcat2 sex_n AgeP3 nap_bi_n
i.medical_history_n vediogames DineOut, || School:
```

```
*sig

xtmixed logInsulin_P3 i.bedtimecat2 sex_n AgeP3 nap_bi_n
i.medical_history_n vediogames BMI3 DineOut, || School:


xtmixed logInsulin_P3 i.sleepdeptcat sex_n AgeP3 nap_bi_n
i.medical_history_n vediogames DineOut, || School:
xtmixed logInsulin_P3 i.sleepdeptsevere sex_n AgeP3 nap_bi_n
i.medical_history_n vediogames DineOut, || School:
xtmixed logInsulin_P3 i.socialgetlag_bi sex_n AgeP3 nap_bi_n
i.medical_history_n vediogames DineOut, || School:
xtmixed logInsulin_P3 i.SJL_bi sex_n AgeP3 nap_bi_n
i.medical_history_n vediogames DineOut, || School:


xtmixed logInsulin_P3 bed12new sex_n AgeP3 nap_bi_n
i.medical_history_n vediogames DineOut, || School:
xtmixed logInsulin_P3 bedtimecat sex_n AgeP3 nap_bi_n
i.medical_history_n vediogames DineOut, || School:

xtmixed logInsulin_P3 bed12 sex_n AgeP3 nap_bi_n i.medical_history_n
vediogames DineOut, || School:
xtmixed logInsulin_P3 bed12bin_n sex_n AgeP3 nap_bi_n
i.medical_history_n vediogames DineOut, || School:
xtmixed logInsulin_P3 bed12_weekend_bi sex_n AgeP3 nap_bi_n
i.medical_history_n vediogames DineOut, || School:

xtmixed logInsulin_P3 i.bedtime123 sex_n AgeP3 nap_bi_n
i.medical_history_n vediogames DineOut, || School:
xtmixed logInsulin_P3 i.bedtimeWDcat sex_n AgeP3 nap_bi_n
i.medical_history_n vediogames DineOut, || School:
xtmixed logInsulin_P3 i.bedtimeWEcat sex_n AgeP3 nap_bi_n
i.medical_history_n vediogames DineOut, || School:

xtmixed logInsulin_P3 i.timeandduration sex_n AgeP3 nap_bi_n
i.medical_history_n vediogames DineOut, || School:
xtmixed logInsulin_P3 sleepdept sex_n AgeP3 nap_bi_n
i.medical_history_n vediogames DineOut, || School:
xtmixed logInsulin_P3 social_getlag sex_n AgeP3 nap_bi_n
i.medical_history_n vediogames DineOut, || School:

xtmixed logInsulin_P3 i.durtime sex_n AgeP3 nap_bi_n
i.medical_history_n vediogames DineOut, || School:

*Fifteenth: logInsulin_Se_P3
xtmixed logInsulin_Se_P3 AverageSleepDuration sex_n AgeP3 nap_bi_n
i.medical_history_n vediogames DineOut, || School:
xtmixed logInsulin_Se_P3 AverageSleepDuration sex_n AgeP3 nap_bi_n
i.medical_history_n vediogames BMI3 DineOut, || School:
```

```
xtmixed logInsulin_Se_P3 Sleep_time_weekday_n sex_n AgeP3 nap_bi_n
i.medical_history_n vediogames DineOut, || School:
xtmixed logInsulin_Se_P3 Sleep_time_weekend_n sex_n AgeP3 nap_bi_n
i.medical_history_n vediogames DineOut, || School:

xtmixed logInsulin_Se_P3 bedtime_both2 sex_n AgeP3 nap_bi_n
i.medical_history_n vediogames DineOut, || School:
xtmixed logInsulin_Se_P3 bed_hour sex_n AgeP3 nap_bi_n
i.medical_history_n vediogames DineOut, || School:
xtmixed logInsulin_Se_P3 bed_weekend sex_n AgeP3 nap_bi_n
i.medical_history_n vediogames DineOut, || School:

xtmixed logInsulin_Se_P3 i.bedtimecat2 sex_n AgeP3 nap_bi_n
i.medical_history_n vediogames DineOut, || School:
*sig
xtmixed logInsulin_Se_P3 i.bedtimeWDcat2 sex_n AgeP3 nap_bi_n
i.medical_history_n vediogames DineOut, || School:
xtmixed logInsulin_Se_P3 i.bedtimeWEcat2 sex_n AgeP3 nap_bi_n
i.medical_history_n vediogames DineOut, || School:

xtmixed logInsulin_Se_P3 i.bedtimecat2 sex_n AgeP3 nap_bi_n
i.medical_history_n vediogames BMI3 DineOut, || School:
xtmixed logInsulin_Se_P3 i.bedtimecat2 sex_n AgeP3 nap_bi_n
i.medical_history_n vediogames  Waist_cm3 BMI3 DineOut, || School:


xtmixed logInsulin_Se_P3 i.sleepdeptcat sex_n AgeP3 nap_bi_n
i.medical_history_n vediogames DineOut, || School:
xtmixed logInsulin_Se_P3 i.sleepdeptsevere sex_n AgeP3 nap_bi_n
i.medical_history_n vediogames DineOut, || School:
xtmixed logInsulin_Se_P3 i.socialgetlag_bi sex_n AgeP3 nap_bi_n
i.medical_history_n vediogames DineOut, || School:

xtmixed logInsulin_Se_P3 i.durtime sex_n AgeP3 nap_bi_n
i.medical_history_n vediogames DineOut, || School:


xtmixed logInsulin_Se_P3 bed12new sex_n AgeP3 nap_bi_n
i.medical_history_n vediogames DineOut, || School:
xtmixed logInsulin_Se_P3 bedtimecat sex_n AgeP3 nap_bi_n
i.medical_history_n vediogames DineOut, || School:
*sig

xtmixed logInsulin_Se_P3 bed12 sex_n AgeP3 nap_bi_n
i.medical_history_n vediogames DineOut, || School:
xtmixed logInsulin_Se_P3 bed12bin_n sex_n AgeP3 nap_bi_n
i.medical_history_n vediogames DineOut, || School:
*Significant
xtmixed logInsulin_Se_P3 bed12_weekend_bi sex_n AgeP3 nap_bi_n
```

```
i.medical_history_n vediogames DineOut, || School:

xtmixed logInsulin_Se_P3 i.bedtime123 sex_n AgeP3 nap_bi_n
i.medical_history_n vediogames DineOut, || School:
xtmixed logInsulin_Se_P3 i.bedtimeWDcat sex_n AgeP3 nap_bi_n
i.medical_history_n vediogames DineOut, || School:
xtmixed logInsulin_Se_P3 i.bedtimeWEcat sex_n AgeP3 nap_bi_n
i.medical_history_n vediogames DineOut, || School:

xtmixed logInsulin_Se_P3 sleepdept sex_n AgeP3 nap_bi_n
i.medical_history_n vediogames DineOut, || School:
xtmixed logInsulin_Se_P3 social_getlag sex_n AgeP3 nap_bi_n
i.medical_history_n vediogames DineOut, || School:
xtmixed logMCP1_Se_P3 i.timeandduration sex_n AgeP3 nap_bi_n
i.medical_history_n vediogames DineOut, || School:


*17: logMCP1_P3
*Mixed Effect model:
xtmixed logMCP1_P3  AverageSleepDuration sex_n AgeP3 nap_bi_n
i.medical_history_n vediogames DineOut, || School:
xtmixed logMCP1_P3  Sleep_time_weekday_n sex_n AgeP3 nap_bi_n
i.medical_history_n vediogames DineOut, || School:
xtmixed logMCP1_P3  Sleep_time_weekend_n sex_n AgeP3 nap_bi_n
i.medical_history_n vediogames DineOut, || School:

xtmixed logMCP1_P3  bedtime_both2 sex_n AgeP3 nap_bi_n
i.medical_history_n vediogames DineOut, || School:
xtmixed logMCP1_P3  bed_hour sex_n AgeP3 nap_bi_n i.medical_history_n
vediogames DineOut, || School:
xtmixed logMCP1_P3  bed_weekend sex_n AgeP3 nap_bi_n
i.medical_history_n vediogames DineOut, || School:

xtmixed logMCP1_P3 i.bedtimecat2 sex_n AgeP3 nap_bi_n
i.medical_history_n vediogames DineOut, || School:
xtmixed logMCP1_P3  i.bedtimeWDcat2 sex_n AgeP3 nap_bi_n
i.medical_history_n vediogames DineOut, || School:
xtmixed logMCP1_P3  i.bedtimeWEcat2 sex_n AgeP3 nap_bi_n
i.medical_history_n vediogames DineOut, || School:

xtmixed logMCP1_P3 i.sleepdeptcat sex_n AgeP3 nap_bi_n
i.medical_history_n vediogames DineOut, || School:
xtmixed logMCP1_P3 i.sleepdeptsevere sex_n AgeP3 nap_bi_n
i.medical_history_n vediogames DineOut, || School:
xtmixed logMCP1_P3 i.socialgetlag_bi sex_n AgeP3 nap_bi_n
i.medical_history_n vediogames DineOut, || School:

xtmixed logMCP1_P3 bed12new sex_n AgeP3 nap_bi_n i.medical_history_n
vediogames DineOut, || School:
```

```
xtmixed logMCP1_P3 bed12 sex_n AgeP3 nap_bi_n i.medical_history_n
vediogames DineOut, || School:
xtmixed logMCP1_P3  bed12bin_n sex_n AgeP3 nap_bi_n
i.medical_history_n vediogames DineOut, || School:
xtmixed logMCP1_P3  bed12_weekend_bi sex_n AgeP3 nap_bi_n
i.medical_history_n vediogames DineOut, || School:

xtmixed logMCP1_P3  i.bedtime123 sex_n AgeP3 nap_bi_n
i.medical_history_n vediogames DineOut, || School:
xtmixed logMCP1_P3  i.bedtimeWDcat sex_n AgeP3 nap_bi_n
i.medical_history_n vediogames DineOut, || School:
xtmixed logMCP1_P3  i.bedtimeWEcat sex_n AgeP3 nap_bi_n
i.medical_history_n vediogames DineOut, || School:

xtmixed logMCP1_P3 i.timeandduration sex_n AgeP3 nap_bi_n
i.medical_history_n vediogames DineOut, || School:
xtmixed logMCP1_P3 sleepdept sex_n AgeP3 nap_bi_n i.medical_history_n
vediogames DineOut, || School:
xtmixed logMCP1_P3 social_getlag sex_n AgeP3 nap_bi_n
i.medical_history_n vediogames DineOut, || School:

xtmixed logMCP1_P3 i.durtime sex_n AgeP3 nap_bi_n i.medical_history_n
vediogames DineOut, || School:


*18: logMCP1_Se_P3
xtmixed logMCP1_Se_P3  AverageSleepDuration sex_n AgeP3 nap_bi_n
i.medical_history_n vediogames DineOut, || School:
xtmixed logMCP1_Se_P3  Sleep_time_weekday_n sex_n AgeP3 nap_bi_n
i.medical_history_n vediogames DineOut, || School:
xtmixed logMCP1_Se_P3  Sleep_time_weekend_n sex_n AgeP3 nap_bi_n
i.medical_history_n vediogames DineOut, || School:

xtmixed logMCP1_Se_P3  bedtime_both2 sex_n AgeP3 nap_bi_n
i.medical_history_n vediogames DineOut, || School:
xtmixed logMCP1_Se_P3  bed_hour sex_n AgeP3 nap_bi_n
i.medical_history_n vediogames DineOut, || School:
xtmixed logMCP1_Se_P3  bed_weekend sex_n AgeP3 nap_bi_n
i.medical_history_n vediogames BMI3 DineOut, || School:

xtmixed logMCP1_Se_P3 i.bedtimecat2 sex_n AgeP3 nap_bi_n
i.medical_history_n vediogames DineOut, || School:
xtmixed logMCP1_Se_P3 i.bedtimeWDcat2 sex_n AgeP3 nap_bi_n
i.medical_history_n vediogames DineOut, || School:
xtmixed logMCP1_Se_P3  i.bedtimeWEcat2 sex_n AgeP3 nap_bi_n
i.medical_history_n vediogames DineOut, || School:

xtmixed logMCP1_Se_P3 i.sleepdeptcat sex_n AgeP3 nap_bi_n
i.medical_history_n vediogames DineOut, || School:
xtmixed logMCP1_Se_P3 i.sleepdeptsevere sex_n AgeP3 nap_bi_n
```

```
i.medical_history_n vediogames DineOut, || School:
xtmixed logMCP1_Se_P3 i.socialgetlag_bi sex_n AgeP3 nap_bi_n
i.medical_history_n vediogames DineOut, || School:

xtmixed logMCP1_Se_P3 bed12new sex_n AgeP3 nap_bi_n
i.medical_history_n vediogames DineOut, || School:

xtmixed logMCP1_Se_P3 bed12 sex_n AgeP3 nap_bi_n i.medical_history_n
vediogames DineOut, || School:
xtmixed logMCP1_Se_P3  bed12bin_n sex_n AgeP3 nap_bi_n
i.medical_history_n vediogames DineOut, || School:
xtmixed logMCP1_Se_P3  bed12_weekend_bi sex_n AgeP3 nap_bi_n
i.medical_history_n vediogames DineOut, || School:

xtmixed logMCP1_Se_P3  i.bedtime123 sex_n AgeP3 nap_bi_n
i.medical_history_n vediogames DineOut, || School:
xtmixed logMCP1_Se_P3  i.bedtimeWDcat sex_n AgeP3 nap_bi_n
i.medical_history_n vediogames DineOut, || School:
xtmixed logMCP1_Se_P3  i.bedtimeWEcat sex_n AgeP3 nap_bi_n
i.medical_history_n vediogames DineOut, || School:

xtmixed logMCP1_Se_P3 i.timeandduration sex_n AgeP3 nap_bi_n
i.medical_history_n vediogames DineOut, || School:
xtmixed logMCP1_Se_P3 sleepdept sex_n AgeP3 nap_bi_n
i.medical_history_n vediogames DineOut, || School:
xtmixed logMCP1_Se_P3 social_getlag sex_n AgeP3 nap_bi_n
i.medical_history_n vediogames DineOut, || School:

xtmixed logMCP1_Se_P3 i.durtime sex_n AgeP3 nap_bi_n
i.medical_history_n vediogames DineOut, || School:


xtmixed logMCP1_Se_P3 sleepdept sex_n AgeP3 nap_bi_n
i.medical_history_n vediogames DineOut, || School:
xtmixed logMCP1_Se_P3 i.sleepdeptcat sex_n AgeP3 nap_bi_n
i.medical_history_n vediogames DineOut, || School:
xtmixed logMCP1_Se_P3 i.sleepdeptsevere sex_n AgeP3 nap_bi_n
i.medical_history_n vediogames DineOut, || School:

xtmixed logMCP1_Se_P3 social_getlag sex_n AgeP3 nap_bi_n
i.medical_history_n vediogames DineOut, || School:
xtmixed logMCP1_Se_P3 i.socialgetlag_bi sex_n AgeP3 nap_bi_n
i.medical_history_n vediogames DineOut, || School:


************************************************Mediation
Analysis*******************************************************
*SEM Method for the analysis for mediation***
ssc install medsem
**********************************************************************
```

```
**************************
**********************************************************************
********************
************************************* BMI as a possible
mediator*********************************
Average categorical but not used for this thesis
* 1: CRP Saliva
*Categorical Average bedtime:
sem (logCRP_P3<-BMI3 bedtime123) (BMI3<-bedtime123), nocapslatent
sem (vediogames DineOut AgeP3 sex_n nap_bi_n bedtime123 -> logCRP_P3)
( vediogames DineOut AgeP3 sex_n nap_bi_n bedtime123-> BMI3)
(vediogames DineOut BMI3 AgeP3 sex_n nap_bi_n -> logCRP_P3),
vce(bootstrap, reps(5000)) nocapslatent
estat teffects
medsem, indep(bedtime123) med(BMI3) dep(logCRP_P3) mcreps(500) rit rid
estat gof, stats(indices residuals)
* BMI not mediator (indirect effect is not stat significant)

* 2: HSCRP
*Categorical Average bedtime:
sem (HSCRPugml<-BMI3 bedtime123) (BMI3<-bedtime123), nocapslatent
sem (vediogames DineOut AgeP3 sex_n nap_bi_n bedtime123 -> HSCRPugml)
( vediogames DineOut AgeP3 sex_n nap_bi_n bedtime123-> BMI3)
(vediogames DineOut BMI3 AgeP3 sex_n nap_bi_n -> HSCRPugml),
vce(bootstrap, reps(5000)) nocapslatent
estat teffects
medsem, indep(bedtime123) med(BMI3) dep(HSCRPugml) mcreps(500) rit rid
estat gof, stats(indices residuals)
* BMI is a mediator for the association between

* 3- IL6 Serum
*Categorical Average bedtime:
sem (IL6_Se_P3<-BMI3 bedtime123) (BMI3<-bedtime123), nocapslatent
sem (vediogames DineOut AgeP3 sex_n nap_bi_n bedtime123 -> IL6_Se_P3)
( vediogames DineOut AgeP3 sex_n nap_bi_n bedtime123-> BMI3)
(vediogames DineOut BMI3 AgeP3 sex_n nap_bi_n -> IL6_Se_P3),
vce(bootstrap, reps(5000)) nocapslatent
estat teffects
medsem, indep(bedtime123) med(BMI3) dep(IL6_Se_P3) mcreps(500) rit rid
estat gof, stats(indices residuals)


* 4- IL-6 saliva
*Categorical Average bedtime:
sem (logIL6_P3<-BMI3 bedtime123) (BMI3<-bedtime123), nocapslatent
sem (vediogames DineOut AgeP3 sex_n nap_bi_n bedtime123 -> logIL6_P3)
( vediogames DineOut AgeP3 sex_n nap_bi_n bedtime123-> BMI3)
(vediogames DineOut BMI3 AgeP3 sex_n nap_bi_n -> logIL6_P3),
vce(bootstrap, reps(5000)) nocapslatent
estat teffects
```

```
medsem, indep(bedtime123) med(BMI3) dep(logIL6_P3) mcreps(500) rit rid
estat gof, stats(indices residuals)

* 5- IL_8 saliva
sem (logIL8_P3<-BMI3 bedtime123) (BMI3<-bedtime123), nocapslatent
sem (vediogames DineOut AgeP3 sex_n nap_bi_n bedtime123 -> logIL8_P3)
( vediogames DineOut AgeP3 sex_n nap_bi_n bedtime123-> BMI3)
(vediogames DineOut BMI3 AgeP3 sex_n nap_bi_n -> logIL8_P3),
vce(bootstrap, reps(5000)) nocapslatent
estat teffects
medsem, indep(bedtime123) med(BMI3) dep(logIL8_P3) mcreps(500) rit rid
estat gof, stats(indices residuals)

* 5- IL_8 serum
sem (logIL8_Se_P3<-BMI3 bedtime123) (BMI3<-bedtime123), nocapslatent
sem (vediogames DineOut AgeP3 sex_n nap_bi_n bedtime123 ->
logIL8_Se_P3) ( vediogames DineOut AgeP3 sex_n nap_bi_n bedtime123->
BMI3) (vediogames DineOut BMI3 AgeP3 sex_n nap_bi_n -> logIL8_Se_P3),
vce(bootstrap, reps(5000)) nocapslatent
estat teffects
medsem, indep(bedtime123) med(BMI3) dep(logIL8_Se_P3) mcreps(500) rit
rid
estat gof, stats(indices residuals)

* 6- IL_10 serum
sem (logIL10_Se <-BMI3 bedtime123) (BMI3<-bedtime123), nocapslatent
sem (vediogames DineOut AgeP3 sex_n nap_bi_n bedtime123 ->
logIL10_Se ) ( vediogames DineOut AgeP3 sex_n nap_bi_n bedtime123->
BMI3) (vediogames DineOut BMI3 AgeP3 sex_n nap_bi_n -> logIL10_Se),
vce(bootstrap, reps(5000)) nocapslatent
estat teffects
medsem, indep(bedtime123) med(BMI3) dep(logIL10_Se ) mcreps(500) rit
rid
estat gof, stats(indices residuals)

* 6- VEGF saliva
sem (VEGF_P3 <-BMI3 bedtime123) (BMI3<-bedtime123), nocapslatent
sem (vediogames DineOut AgeP3 sex_n nap_bi_n bedtime123 -> VEGF_P3 )
( vediogames DineOut AgeP3 sex_n nap_bi_n bedtime123-> BMI3)
(vediogames DineOut BMI3 AgeP3 sex_n nap_bi_n -> VEGF_P3),
vce(bootstrap, reps(5000)) nocapslatent
estat teffects
medsem, indep(bedtime123) med(BMI3) dep(VEGF_P3) mcreps(500) rit rid
estat gof, stats(indices residuals)

* 6- VEGF Serum
sem (logVEGF_Se_P3 <-BMI3 bedtime123) (BMI3<-bedtime123), nocapslatent
sem (vediogames DineOut AgeP3 sex_n nap_bi_n bedtime123 ->
logVEGF_Se_P3 ) ( vediogames DineOut AgeP3 sex_n nap_bi_n bedtime123->
BMI3) (vediogames DineOut BMI3 AgeP3 sex_n nap_bi_n -> logVEGF_Se_P3),
```

```
vce(bootstrap, reps(5000)) nocapslatent
estat teffects
medsem, indep(bedtime123) med(BMI3) dep(logVEGF_Se_P3) mcreps(500) rit
rid
estat gof, stats(indices residuals)

* 6- MCP-1 saliva
sem (logMCP1_P3 <-BMI3 bedtime123) (BMI3<-bedtime123), nocapslatent
sem (vediogames DineOut AgeP3 sex_n nap_bi_n bedtime123 ->
logMCP1_P3 ) ( vediogames DineOut AgeP3 sex_n nap_bi_n bedtime123->
BMI3) (vediogames DineOut BMI3 AgeP3 sex_n nap_bi_n -> logMCP1_P3),
vce(bootstrap, reps(5000)) nocapslatent
estat teffects
medsem, indep(bedtime123) med(BMI3) dep(logMCP1_P3) mcreps(500) rit
rid
estat gof, stats(indices residuals)

* 6- MCP-1 serum
sem (logMCP1_Se_P3 <-BMI3 bedtime123) (BMI3<-bedtime123), nocapslatent
sem (vediogames DineOut AgeP3 sex_n nap_bi_n bedtime123 ->
logMCP1_Se_P3 ) ( vediogames DineOut AgeP3 sex_n nap_bi_n bedtime123->
BMI3) (vediogames DineOut BMI3 AgeP3 sex_n nap_bi_n -> logMCP1_Se_P3),
vce(bootstrap, reps(5000)) nocapslatent
estat teffects
medsem, indep(bedtime123) med(BMI3) dep(logMCP1_Se_P3) mcreps(500) rit
rid
estat gof, stats(indices residuals)

*7- Insulin saliva:
sem (logInsulin_P3 <-BMI3 bedtime123) (BMI3<-bedtime123), nocapslatent
sem (vediogames DineOut AgeP3 sex_n nap_bi_n bedtime123 ->
logInsulin_P3 ) ( vediogames DineOut AgeP3 sex_n nap_bi_n bedtime123->
BMI3) (vediogames DineOut BMI3 AgeP3 sex_n nap_bi_n -> logInsulin_P3),
vce(bootstrap, reps(5000)) nocapslatent
estat teffects
medsem, indep(bedtime123) med(BMI3) dep(logInsulin_P3 ) mcreps(500)
rit rid
estat gof, stats(indices residuals)

*7- Insulin serum:
sem (logInsulin_Se_P3 <-BMI3 bedtime123) (BMI3<-bedtime123),
nocapslatent
sem (vediogames DineOut AgeP3 sex_n nap_bi_n bedtime123 ->
logInsulin_Se_P3 ) ( vediogames DineOut AgeP3 sex_n nap_bi_n
bedtime123-> BMI3) (vediogames DineOut BMI3 AgeP3 sex_n nap_bi_n ->
logInsulin_Se_P3), vce(bootstrap, reps(5000)) nocapslatent
estat teffects
medsem, indep(bedtime123) med(BMI3) dep(logInsulin_Se_P3) mcreps(500)
rit rid
estat gof, stats(indices residuals)
```

```
*7- Adiponectin saliva:
sem (logAdiponectin_P3 <-BMI3 bedtime123) (BMI3<-bedtime123),
nocapslatent
estat teffects
medsem, indep(bedtime123) med(BMI3) dep(logAdiponectin_P3) mcreps(500)
rit rid
estat gof, stats(indices residuals)

*8- Adiponectin serum
sem (logAdiponectin_Se_P3 <-BMI3 bedtime123) (BMI3<-bedtime123),
nocapslatent
estat teffects
medsem, indep(bedtime123) med(BMI3) dep(logAdiponectin_Se_P3)
mcreps(500) rit rid
estat gof, stats(indices residuals)

* leptin saliva:
sem (logLeptin_P3 <-BMI3 bedtime123) (BMI3<-bedtime123), nocapslatent
estat teffects
medsem, indep(bedtime123) med(BMI3) dep(logLeptin_P3) mcreps(500) rit
rid
estat gof, stats(indices residuals)
***********************
* 3- IL6 Serum (log)
*Categorical Average bedtime:
sem (logIL6_Se_P3<-BMI3 bedtime123) (BMI3<-bedtime123), nocapslatent
sem (vediogames DineOut AgeP3 sex_n nap_bi_n bedtime123 ->
logIL6_Se_P3) ( vediogames DineOut AgeP3 sex_n nap_bi_n bedtime123->
BMI3) (vediogames DineOut BMI3 AgeP3 sex_n nap_bi_n -> logIL6_Se_P3),
vce(bootstrap, reps(5000)) nocapslatent
estat teffects
medsem, indep(bedtime123) med(BMI3) dep(logIL6_Se_P3) mcreps(500) rit
rid
estat gof, stats(indices residuals)

* 6- IL_10 saliva (log)
sem (logIL10_P3 <-BMI3 bedtime123) (BMI3<-bedtime123), nocapslatent
sem (vediogames DineOut AgeP3 sex_n nap_bi_n bedtime123 ->
logIL10_P3 ) ( vediogames DineOut AgeP3 sex_n nap_bi_n bedtime123->
BMI3) (vediogames DineOut BMI3 AgeP3 sex_n nap_bi_n -> logIL10_P3),
vce(bootstrap, reps(5000)) nocapslatent
estat teffects
medsem, indep(bedtime123) med(BMI3) dep(logIL10_P3) mcreps(500) rit
rid
estat gof, stats(indices residuals)

*7- Leptin serum:
sem (logLeptin_Se_P3 <-BMI3 bedtime123) (BMI3<-bedtime123),
nocapslatent
```

```
sem (vediogames DineOut AgeP3 sex_n nap_bi_n bedtime123 ->
logLeptin_Se_P3 ) ( vediogames DineOut AgeP3 sex_n nap_bi_n
bedtime123-> BMI3) (vediogames DineOut BMI3 AgeP3 sex_n nap_bi_n ->
logLeptin_Se_P3), vce(bootstrap, reps(5000)) nocapslatent
estat teffects
medsem, indep(bedtime123) med(BMI3) dep(logLeptin_Se_P3) mcreps(500)
rit rid
estat gof, stats(indices residuals)

**********************************************************************
*******************
********************************** Waist Circumference as a possible
mediator************
*IL-10 serum
sem (logIL10_Se<-Waist_cm3 bedtime123) (Waist_cm3<-bedtime123),
nocapslatent
sem (vediogames DineOut AgeP3 sex_n nap_bi_n bedtime123 -> logIL10_Se)
( vediogames DineOut AgeP3 sex_n nap_bi_n bedtime123-> Waist_cm3)
(vediogames DineOut Waist_cm3 AgeP3 sex_n nap_bi_n -> logIL10_Se),
vce(bootstrap, reps(5000)) nocapslatent
estat teffects
medsem, indep(bedtime123) med(Waist_cm3) dep(logIL10_Se) mcreps(500)
rit rid zlc
estat gof, stats(indices residuals)

*Insulin saliva:
sem (logInsulin_P3<-Waist_cm3 bedtime123) (Waist_cm3<-bedtime123),
nocapslatent
sem (vediogames DineOut AgeP3 sex_n nap_bi_n bedtime123 ->
logInsulin_P3) ( vediogames DineOut AgeP3 sex_n nap_bi_n bedtime123->
Waist_cm3) (vediogames DineOut Waist_cm3 AgeP3 sex_n nap_bi_n ->
logInsulin_P3), vce(bootstrap, reps(5000)) nocapslatent
estat teffects
medsem, indep(bedtime123) med(Waist_cm3) dep(logInsulin_P3)
mcreps(500) rit rid zlc
estat gof, stats(indices residuals)
*********************
* 3- IL6 Serum (log)
*Categorical Average bedtime:
sem (logIL6_Se_P3<-Waist_cm3 bedtime123) (Waist_cm3<-bedtime123),
nocapslatent
sem (vediogames DineOut AgeP3 sex_n nap_bi_n bedtime123 ->
logIL6_Se_P3) ( vediogames DineOut AgeP3 sex_n nap_bi_n bedtime123->
Waist_cm3) (vediogames DineOut Waist_cm3 AgeP3 sex_n nap_bi_n ->
logIL6_Se_P3), vce(bootstrap, reps(5000)) nocapslatent
estat teffects
medsem, indep(bedtime123) med(Waist_cm3) dep(logIL6_Se_P3) mcreps(500)
rit rid
estat gof, stats(indices residuals)
```

```
* 6- IL_10 saliva (log)
sem (logIL10_P3 <-Waist_cm3 bedtime123) (Waist_cm3<-bedtime123),
nocapslatent
sem (vediogames DineOut AgeP3 sex_n nap_bi_n bedtime123 ->
logIL10_P3 ) ( vediogames DineOut AgeP3 sex_n nap_bi_n bedtime123->
Waist_cm3) (vediogames DineOut Waist_cm3 AgeP3 sex_n nap_bi_n ->
logIL10_P3), vce(bootstrap, reps(5000)) nocapslatent
estat teffects
medsem, indep(bedtime123) med(Waist_cm3) dep(logIL10_P3) mcreps(500)
rit rid
estat gof, stats(indices residuals)

*7- Leptin serum:
sem (logLeptin_Se_P3 <-Waist_cm3 bedtime123) (Waist_cm3<-bedtime123),
nocapslatent
sem (vediogames DineOut AgeP3 sex_n nap_bi_n bedtime123 ->
logLeptin_Se_P3 ) ( vediogames DineOut AgeP3 sex_n nap_bi_n
bedtime123-> Waist_cm3) (vediogames DineOut Waist_cm3 AgeP3 sex_n
nap_bi_n -> logLeptin_Se_P3), vce(bootstrap, reps(5000)) nocapslatent
estat teffects
medsem, indep(bedtime123) med(Waist_cm3) dep(logLeptin_Se_P3)
mcreps(500) rit rid
estat gof, stats(indices residuals)

* 6- VEGF saliva
sem (VEGF_P3 <-Waist_cm3 bedtime123) (Waist_cm3<-bedtime123),
nocapslatent
estat teffects
medsem, indep(bedtime123) med(Waist_cm3) dep(VEGF_P3) mcreps(500) rit
rid
estat gof, stats(indices residuals)

* 6- VEGF Serum
sem (logVEGF_Se_P3 <-Waist_cm3 bedtime123) (Waist_cm3<-bedtime123),
nocapslatent
estat teffects
medsem, indep(bedtime123) med(Waist_cm3) dep(logVEGF_Se_P3)
mcreps(500) rit rid
estat gof, stats(indices residuals)

* 6- MCP-1 saliva
sem (logMCP1_P3 <-Waist_cm3 bedtime123) (Waist_cm3<-bedtime123),
nocapslatent
estat teffects
medsem, indep(bedtime123) med(Waist_cm3) dep(logMCP1_P3) mcreps(500)
rit rid
estat gof, stats(indices residuals)

* 6- MCP-1 serum
sem (logMCP1_Se_P3 <-Waist_cm3 bedtime123) (Waist_cm3<-bedtime123),
```

```
nocapslatent
estat teffects
medsem, indep(bedtime123) med(Waist_cm3) dep(logMCP1_Se_P3)
mcreps(500) rit rid
estat gof, stats(indices residuals)


*7- Adiponectin saliva:
sem (logAdiponectin_P3 <-Waist_cm3 bedtime123) (Waist_cm3<-
bedtime123), nocapslatent
estat teffects
medsem, indep(bedtime123) med(Waist_cm3) dep(logAdiponectin_P3)
mcreps(500) rit rid
estat gof, stats(indices residuals)

*8- Adiponectin serum
sem (logAdiponectin_Se_P3 <-Waist_cm3 bedtime123) (Waist_cm3<-
bedtime123), nocapslatent
estat teffects
medsem, indep(bedtime123) med(Waist_cm3) dep(logAdiponectin_Se_P3)
mcreps(500) rit rid
estat gof, stats(indices residuals)

* leptin saliva:
sem (logLeptin_P3 <-Waist_cm3 bedtime123) (Waist_cm3<-bedtime123),
nocapslatent
estat teffects
medsem, indep(bedtime123) med(Waist_cm3) dep(logLeptin_P3) mcreps(500)
rit rid
estat gof, stats(indices residuals)

*CRP saliva
sem (logCRP_P3 <-Waist_cm3 bedtime123) (Waist_cm3<-bedtime123),
nocapslatent
estat teffects
medsem, indep(bedtime123) med(Waist_cm3) dep(logCRP_P3) mcreps(500)
rit rid
estat gof, stats(indices residuals)

* IL-8 saliva:
sem (logIL8_P3 <-Waist_cm3 bedtime123) (Waist_cm3<-bedtime123),
nocapslatent
estat teffects
medsem, indep(bedtime123) med(Waist_cm3) dep(logIL8_P3 ) mcreps(500)
rit rid
estat gof, stats(indices residuals)

**********************************************************************
*******************
************************************ Nap as a possible
```

```
mediator************************
*IL-10 serum:
sem (logIL10_Se<-nap_bi_n bedtime123) (nap_bi_n<-bedtime123),
nocapslatent
sem (vediogames DineOut AgeP3 sex_n bedtime123 -> logIL10_Se)
( vediogames DineOut AgeP3 sex_n bedtime123-> nap_bi_n) (vediogames
DineOut nap_bi_n AgeP3 sex_n -> logIL10_Se), vce(bootstrap,
reps(5000)) nocapslatent
estat teffects
medsem, indep(bedtime123) med(nap_bi_n) dep(logIL10_Se) mcreps(500)
rit rid zlc

*Insulin saliva:
sem (logInsulin_P3<-nap_bi_n bedtime123) (nap_bi_n<-bed12bin_n),
nocapslatent
sem (vediogames DineOut AgeP3 sex_n bedtime123 -> logInsulin_P3)
( vediogames DineOut AgeP3 sex_n bedtime123-> nap_bi_n) (vediogames
DineOut nap_bi_n AgeP3 sex_n -> logInsulin_P3), vce(bootstrap,
reps(5000)) nocapslatent
estat teffects
medsem, indep(bedtime123) med(nap_bi_n) dep(logInsulin_P3) mcreps(500)
rit rid zlc
*******************
* 3- IL6 Serum (log)
*Categorical Average bedtime:
sem (logIL6_Se_P3<-nap_bi_n bedtime123) (nap_bi_n<-bed12bin_n),
nocapslatent
sem (vediogames DineOut AgeP3 sex_n bedtime123 -> logIL6_Se_P3)
( vediogames DineOut AgeP3 sex_n bedtime123-> nap_bi_n) (vediogames
DineOut nap_bi_n AgeP3 sex_n -> logIL6_Se_P3), vce(bootstrap,
reps(5000)) nocapslatent
estat teffects
medsem, indep(bedtime123) med(nap_bi_n) dep(logIL6_Se_P3) mcreps(500)
rit rid zlc

* 6- IL_10 saliva (log)
sem (logIL10_P3 <-nap_bi_n bedtime123) (nap_bi_n<-bedtime123),
nocapslatent
sem (vediogames DineOut AgeP3 sex_n bedtime123 -> logIL10_P3 )
( vediogames DineOut AgeP3 sex_n bedtime123-> nap_bi_n) (vediogames
DineOut nap_bi_n AgeP3 sex_n -> logIL10_P3), vce(bootstrap,
reps(5000)) nocapslatent
estat teffects
medsem, indep(bedtime123) med(nap_bi_n) dep(logIL10_P3) mcreps(500)
rit rid
estat gof, stats(indices residuals)

*7- Leptin serum:
sem (logLeptin_Se_P3 <-nap_bi_n bedtime123) (nap_bi_n<-bedtime123),
nocapslatent
```

```
sem (vediogames DineOut AgeP3 sex_n bedtime123 -> logLeptin_Se_P3 )
( vediogames DineOut AgeP3 sex_n bedtime123-> nap_bi_n) (vediogames
DineOut nap_bi_n AgeP3 sex_n -> logLeptin_Se_P3), vce(bootstrap,
reps(5000)) nocapslatent
estat teffects
medsem, indep(bedtime123) med(nap_bi_n) dep(logLeptin_Se_P3)
mcreps(500) rit rid
estat gof, stats(indices residuals)

* 6- VEGF saliva
sem (VEGF_P3 <-nap_bi_n bedtime123) (nap_bi_n<-bedtime123),
nocapslatent
sem (vediogames DineOut AgeP3 sex_n bedtime123 ->VEGF_P3) ( vediogames
DineOut AgeP3 sex_n bedtime123-> nap_bi_n) (vediogames DineOut
nap_bi_n AgeP3 sex_n -> VEGF_P3), vce(bootstrap, reps(5000))
nocapslatent
estat teffects
medsem, indep(bedtime123) med(nap_bi_n) dep(VEGF_P3) mcreps(500) rit
rid
estat gof, stats(indices residuals)

* 6- VEGF Serum
sem (logVEGF_Se_P3 <-nap_bi_n bedtime123) (nap_bi_n<-bedtime123),
nocapslatent
sem (vediogames DineOut AgeP3 sex_n bedtime123 -> logVEGF_Se_P3)
( vediogames DineOut AgeP3 sex_n bedtime123-> nap_bi_n) (vediogames
DineOut nap_bi_n AgeP3 sex_n -> logVEGF_Se_P3), vce(bootstrap,
reps(5000)) nocapslatent
estat teffects
medsem, indep(bedtime123) med(nap_bi_n) dep(logVEGF_Se_P3) mcreps(500)
rit rid
estat gof, stats(indices residuals)

* 6- MCP-1 saliva
sem (logMCP1_P3 <-nap_bi_n bedtime123) (nap_bi_n<-bedtime123),
nocapslatent
sem (vediogames DineOut AgeP3 sex_n bedtime123 ->logMCP1_P3)
( vediogames DineOut AgeP3 sex_n bedtime123-> nap_bi_n) (vediogames
DineOut nap_bi_n AgeP3 sex_n -> logMCP1_P3), vce(bootstrap,
reps(5000)) nocapslatent
estat teffects
medsem, indep(bedtime123) med(nap_bi_n) dep(logMCP1_P3) mcreps(500)
rit rid
estat gof, stats(indices residuals)

* 6- MCP-1 serum
sem (logMCP1_Se_P3 <-nap_bi_n bedtime123) (nap_bi_n<-bedtime123),
nocapslatent
sem (vediogames DineOut AgeP3 sex_n bedtime123 ->logMCP1_Se_P3)
( vediogames DineOut AgeP3 sex_n bedtime123-> nap_bi_n) (vediogames
```

```
DineOut nap_bi_n AgeP3 sex_n -> logMCP1_Se_P3), vce(bootstrap,
reps(5000)) nocapslatent
estat teffects
medsem, indep(bedtime123) med(nap_bi_n) dep(logMCP1_Se_P3) mcreps(500)
rit rid
estat gof, stats(indices residuals)


*7- Adiponectin saliva:
sem (logAdiponectin_P3 <-nap_bi_n bedtime123) (nap_bi_n<-bedtime123),
nocapslatent
sem (vediogames DineOut AgeP3 sex_n bedtime123 ->logAdiponectin_P3)
( vediogames DineOut AgeP3 sex_n bedtime123-> nap_bi_n) (vediogames
DineOut nap_bi_n AgeP3 sex_n -> logAdiponectin_P3), vce(bootstrap,
reps(5000)) nocapslatent
estat teffects
medsem, indep(bedtime123) med(nap_bi_n) dep(logAdiponectin_P3)
mcreps(500) rit rid
estat gof, stats(indices residuals)

*8- Adiponectin serum
sem (logAdiponectin_Se_P3 <-nap_bi_n bedtime123) (nap_bi_n<-
bedtime123), nocapslatent
estat teffects
medsem, indep(bedtime123) med(nap_bi_n) dep(logAdiponectin_Se_P3)
mcreps(500) rit rid
estat gof, stats(indices residuals)

* leptin saliva:
sem (logLeptin_P3 <-nap_bi_n bedtime123) (nap_bi_n<-bedtime123),
nocapslatent
estat teffects
medsem, indep(bedtime123) med(nap_bi_n) dep(logLeptin_P3) mcreps(500)
rit rid
estat gof, stats(indices residuals)

*CRP saliva
sem (logCRP_P3 <-nap_bi_n bedtime123) (nap_bi_n<-bedtime123),
nocapslatent
estat teffects
medsem, indep(bedtime123) med(nap_bi_n) dep(logCRP_P3) mcreps(500) rit
rid
estat gof, stats(indices residuals)

* IL-8 saliva:
sem (logIL8_P3 <-nap_bi_n bedtime123) (nap_bi_n<-bedtime123),
nocapslatent
estat teffects
medsem, indep(bedtime123) med(nap_bi_n) dep(logIL8_P3 ) mcreps(500)
rit rid
```

```
estat gof, stats(indices residuals)
**********************************************************************
********************
**********************************************************************
********************

*Check if BMI is a confounders or EM

*First: Confounding testing:
*Serum CRP
xtmixed logHSCRPugml i.bedtimecat2 sex_n AgeP3 nap_bi_n
i.medical_history_n vediogames DineOut, || School:
estat ic
xtmixed logHSCRPugml i.bedtimecat2 sex_n AgeP3 nap_bi_n
i.medical_history_n vediogames BMI3 DineOut, || School:
estat ic
*BMI is a confounder
xtmixed logCRP_P3 i.bedtimecat2 sex_n AgeP3 nap_bi_n
i.medical_history_n vediogames DineOut, || School:
estat ic
xtmixed logCRP_P3 i.bedtimecat2 sex_n AgeP3 nap_bi_n
i.medical_history_n vediogames BMI3 DineOut, || School:
estat ic
* is a confounder
*IL-6 saliva
xtmixed logIL6_P3 bedtimecat2 sex_n AgeP3 nap_bi_n i.medical_history_n
vediogames DineOut, || School:
xtmixed logIL6_P3 bedtimecat2 sex_n AgeP3 nap_bi_n i.medical_history_n
vediogames BMI3 DineOut, || School:
*BMI not confounder
xtmixed logIL8_Se_P3 bedtimecat2 sex_n AgeP3 nap_bi_n
i.medical_history_n vediogames DineOut, || School:
xtmixed logIL8_Se_P3 bedtimecat2 sex_n AgeP3 nap_bi_n
i.medical_history_n vediogames BMI3 DineOut, || School:
*BMI not confounder
xtmixed logIL10_Se bedtimecat2 sex_n AgeP3 nap_bi_n
i.medical_history_n vediogames DineOut, || School:
estat ic
xtmixed logIL10_Se bedtimecat2 sex_n AgeP3 nap_bi_n
i.medical_history_n vediogames BMI3 DineOut, || School:
estat ic
*BMI not confounder
xtmixed logIL10_P3 bedtimecat2 sex_n AgeP3 nap_bi_n
i.medical_history_n vediogames DineOut, || School:
estat ic
xtmixed logIL10_P3 bedtimecat2 sex_n AgeP3 nap_bi_n
i.medical_history_n vediogames BMI3 DineOut, || School:
estat ic
xtmixed VEGF_P3 i.bedtimecat2 sex_n AgeP3 nap_bi_n i.medical_history_n
vediogames DineOut, || School:
```

```
estat ic
xtmixed VEGF_P3 i.bedtimecat2 sex_n AgeP3 nap_bi_n i.medical_history_n
vediogames BMI3 DineOut, || School:
estat ic
*in a confounder
xtmixed logVEGF_Se_P3 i.bedtimecat2 sex_n AgeP3 nap_bi_n
i.medical_history_n vediogames DineOut, || School:
estat ic
xtmixed logVEGF_Se_P3 i.bedtimecat2 sex_n AgeP3 nap_bi_n
i.medical_history_n vediogames BMI3 DineOut, || School:
estat ic
*Not a confounder
xtmixed logMCP1_P3 i.bedtimecat2 sex_n AgeP3 nap_bi_n
i.medical_history_n vediogames DineOut, || School:
estat ic
xtmixed logMCP1_P3 i.bedtimecat2 sex_n AgeP3 nap_bi_n
i.medical_history_n vediogames BMI3 DineOut, || School:
estat ic
*A confounder
xtmixed logMCP1_Se_P3 i.bedtimecat2 sex_n AgeP3 nap_bi_n
i.medical_history_n vediogames DineOut, || School:
estat ic
xtmixed logMCP1_Se_P3 i.bedtimecat2 sex_n AgeP3 nap_bi_n
i.medical_history_n vediogames BMI3 DineOut, || School:
estat ic
*A confounder
xtmixed logAdiponectin_P3 i.bedtimecat2 sex_n AgeP3 nap_bi_n
i.medical_history_n vediogames DineOut, || School:
estat ic
xtmixed logAdiponectin_P3 i.bedtimecat2 sex_n AgeP3 nap_bi_n
i.medical_history_n vediogames BMI3 DineOut, || School:
estat ic
*A confounder
xtmixed logAdiponectin_Se_P3 i.bedtimecat2 sex_n AgeP3 nap_bi_n
i.medical_history_n vediogames DineOut, || School:
estat ic
xtmixed logAdiponectin_Se_P3 i.bedtimecat2 sex_n AgeP3 nap_bi_n
i.medical_history_n vediogames BMI3 DineOut, || School:
estat ic
*A confounder
xtmixed logLeptin_P3 bedtimecat2 sex_n AgeP3 nap_bi_n
i.medical_history_n vediogames DineOut, || School:
estat ic
xtmixed logLeptin_P3 bedtimecat2 sex_n AgeP3 nap_bi_n
i.medical_history_n vediogames BMI3 DineOut, || School:
estat ic
*not a confounder
xtmixed logLeptin_Se_P3 bedtimecat2 sex_n AgeP3 nap_bi_n
i.medical_history_n vediogames DineOut, || School:
estat ic
```

```
xtmixed logLeptin_Se_P3 bedtimecat2 sex_n AgeP3 nap_bi_n
i.medical_history_n vediogames BMI3 DineOut, || School:
estat ic
*BMI is Confounder
xtmixed logInsulin_P3 bedtimecat2 sex_n AgeP3 nap_bi_n
i.medical_history_n vediogames DineOut, || School:
estat ic
xtmixed logInsulin_P3 bedtimecat2 sex_n AgeP3 nap_bi_n
i.medical_history_n vediogames BMI3 DineOut, || School:
estat ic
*BMI is a confounder
xtmixed logInsulin_Se_P3 bedtimecat2 sex_n AgeP3 nap_bi_n
i.medical_history_n vediogames DineOut, || School:
estat ic
xtmixed logInsulin_Se_P3 bedtimecat2 sex_n AgeP3 nap_bi_n
i.medical_history_n vediogames BMI3 DineOut, || School:
estat ic
*BMI is a confounder
xtmixed logIL6_Se_P3 bedtimecat2 sex_n AgeP3 nap_bi_n
i.medical_history_n vediogames DineOut, || School:
estat ic
xtmixed logIL6_Se_P3 bedtimecat2 sex_n AgeP3 nap_bi_n
i.medical_history_n vediogames BMI3 DineOut, || School:
estat ic
*BMI is a confounder



*second: check if BMI in an Effect modifier:
gen bedbmi = bedtimecat2 * BMI3
xtmixed logIL6_P3 bedtimecat2 sex_n AgeP3 nap_bi_n i.medical_history_n
vediogames BMI3 bedbmi DineOut, || School:
xtmixed logIL8_Se_P3 bedtimecat2 sex_n AgeP3 nap_bi_n
i.medical_history_n vediogames BMI3 bedbmi DineOut, || School:
xtmixed logIL10_Se bedtimecat2 sex_n AgeP3 nap_bi_n
i.medical_history_n vediogames BMI3 bedbmi DineOut, || School:
xtmixed logIL10_P3 bedtimecat2 sex_n AgeP3 nap_bi_n
i.medical_history_n vediogames BMI3 bedbmi DineOut, || School:
xtmixed logInsulin_P3 bedtimecat2 sex_n AgeP3 nap_bi_n
i.medical_history_n vediogames BMI3 bedbmi DineOut, || School:

gen bedbmi_binary = bedtimecat2 * OBWHO3_n
xtmixed logIL6_P3 bedtimecat2 sex_n AgeP3 nap_bi_n i.medical_history_n
vediogames BMI3 bedbmi_binary DineOut, || School:
xtmixed logIL8_Se_P3 bedtimecat2 sex_n AgeP3 nap_bi_n
i.medical_history_n vediogames BMI3 bedbmi_binary DineOut, || School:
xtmixed logIL10_Se bedtimecat2 sex_n AgeP3 nap_bi_n
i.medical_history_n vediogames BMI3 bedbmi_binary DineOut, || School:
xtmixed logIL10_P3 bedtimecat2 sex_n AgeP3 nap_bi_n
i.medical_history_n vediogames BMI3 bedbmi_binary DineOut, || School:
```

```
xtmixed logInsulin_P3 bedtimecat2 sex_n AgeP3 nap_bi_n
i.medical_history_n vediogames BMI3 bedbmi_binary DineOut, || School:

xtmixed logIL10_Se i.bedtimecat2 sex_n AgeP3 nap_bi_n
i.medical_history_n vediogames BMI3 bedbmi_binary DineOut, || School:


regress logIL10_Se bedtimecat2 School sex_n AgeP3 nap_bi_n
i.medical_history_n vediogames DineOut if OBWHO3_n == 0
regress logIL10_Se bedtimecat2 School sex_n AgeP3 nap_bi_n
i.medical_history_n vediogames DineOut if OBWHO3_n == 1
regress logIL10_Se bedtimecat2 School sex_n AgeP3 nap_bi_n
i.medical_history_n vediogames DineOut OBWHO3_n
regress logIL10_Se bedtimecat2 OBWHO3_n

gen bedtine_con_BMI = bedtime_both2 * OBWHO3_n
xtmixed logIL10_Se bedtime_both2 sex_n AgeP3 nap_bi_n
i.medical_history_n vediogames BMI3 bedtine_con_BMI DineOut, ||
School:

regress logIL10_Se bedtime_both2 School sex_n AgeP3 nap_bi_n
i.medical_history_n vediogames DineOut if OBWHO3_n == 0
regress logIL10_Se bedtime_both2 School sex_n AgeP3 nap_bi_n
i.medical_history_n vediogames DineOut if OBWHO3_n == 1
regress logIL10_Se bedtime_both2 School sex_n AgeP3 nap_bi_n
i.medical_history_n vediogames DineOut
regress logIL10_Se bedtime_both2

xtmixed logIL10_P3 bedtime_both2 sex_n AgeP3 nap_bi_n
i.medical_history_n vediogames BMI3 bedtine_con_BMI DineOut, ||
School:

if OBWHO3_n == 0, robust
**********************************************************************
**

*checking the correlation between BMI and WC to see if there is co-
linearity or not:
regress logInsulin_Se_P3 Waist_cm3 BMI3 DineOut
vif
*This produces a VIF value for each of the explanatory variables in
the model. The value for VIF starts at 1 and has no upper limit. A
general rule of thumb for interpreting VIFs is as follows:
*A value of 1 indicates there is no correlation between a given
explanatory variable and any other explanatory variables in the model.
*A value between 1 and 5 indicates moderate correlation between a
given explanatory variable and other explanatory variables in the
model, but this is often not severe enough to require attention.
*A value greater than 5 indicates potentially severe correlation
between a given explanatory variable and other explanatory variables
```

```
in the model. In this case, the coefficient estimates and p-values in
the regression output are likely unreliable.
*Both VIF values for waist circumferenc and BMI are below 5, which
indicates that multicollinearity is no longer a problem in the model.

corr Waist_cm3 BMI3


**********************************************************************
***********************************
*Dr. Redline
**********************NAP as : (Abeer ==> suspected effect
modifier)***********************
*Generating interaction term:
gen napavesleep = nap_bi_n * AverageSleepDuration
gen napSleep_time_wekday = nap_bi_n * Sleep_time_weekday
gen napbed12 = nap_bi_n * bed12
gen napbed12bin_n = nap * bed12bin_n

*First: HSCRP
regress HSCRPugml AverageSleepDuration sex_n i.School AgeP3 nap_bi_n
napavesleep i.medical_history_n vediogames DineOut, robust
xtmixed HSCRPugml AverageSleepDuration sex_n AgeP3 nap_bi_n
napavesleep i.medical_history_n vediogames DineOut, || School:

regress HSCRPugml Sleep_time_weekday sex_n i.School AgeP3 nap_bi_n
napSleep_time_wekday i.medical_history_n vediogames DineOut, robust
xtmixed HSCRPugml Sleep_time_weekday sex_n AgeP3 nap_bi_n
napSleep_time_wekday i.medical_history_n vediogames DineOut, ||
School:

regress HSCRPugml bed12 sex_n i.School AgeP3 nap_bi_n napbed12
i.medical_history_n vediogames DineOut, robust
xtmixed HSCRPugml bed12 sex_n AgeP3 nap_bi_n napbed12
i.medical_history_n vediogames DineOut, || School:

regress HSCRPugml bed12bin_n sex_n i.School AgeP3 nap_bi_n
napbed12bin_n i.medical_history_n vediogames DineOut, robust
xtmixed HSCRPugml bed12bin_n sex_n AgeP3 nap_bi_n napbed12bin_n
i.medical_history_n vediogames DineOut, || School:

*Second: IL6 Serum
regress IL6_Se_P3 AverageSleepDuration sex_n i.School AgeP3 nap_bi_n
napavesleep i.medical_history_n vediogames DineOut, robust

regress IL6_Se_P3 Sleep_time_weekday sex_n i.School AgeP3 nap_bi_n
napSleep_time_wekday i.medical_history_n vediogames DineOut, robust

regress IL6_Se_P3 bed12 sex_n i.School AgeP3 nap_bi_n napbed12
i.medical_history_n vediogames DineOut, robust
```

```
regress IL6_Se_P3 bed12bin_n sex_n i.School AgeP3 nap_bi_n
napbed12bin_n i.medical_history_n vediogames DineOut, robust

*Third: IL6 Saliva ==> log
regress logIL6_P3 AverageSleepDuration sex_n i.School AgeP3 nap_bi_n
napavesleep i.medical_history_n vediogames DineOut, robust

regress logIL6_P3 Sleep_time_weekday sex_n i.School AgeP3 nap_bi_n
napSleep_time_wekday i.medical_history_n vediogames DineOut, robust

regress logIL6_P3 bed12 sex_n i.School AgeP3 nap_bi_n napbed12
i.medical_history_n vediogames DineOut, robust

regress logIL6_P3 bed12bin_n sex_n i.School AgeP3 nap_bi_n
napbed12bin_n i.medical_history_n vediogames DineOut, robust

***************************************************
**********************************************************
*Check if WC is a confounder or EM:
*First: checking confounding:
xtmixed HSCRPugml i.bedtimecat2 sex_n AgeP3 nap_bi_n
i.medical_history_n vediogames DineOut, || School:
estat ic
xtmixed HSCRPugml i.bedtimecat2 sex_n AgeP3 nap_bi_n
i.medical_history_n vediogames Waist_cm3 DineOut, || School:
estat ic
*WC is a confounder

xtmixed logCRP_P3 i.bedtimecat2 sex_n AgeP3 nap_bi_n
i.medical_history_n vediogames DineOut, || School:
estat ic
xtmixed logCRP_P3 i.bedtimecat2 sex_n AgeP3 nap_bi_n
i.medical_history_n vediogames Waist_cm3 DineOut, || School:
estat ic
*is a confounder
xtmixed logIL6_P3 i.bedtimecat2 sex_n AgeP3 nap_bi_n
i.medical_history_n vediogames DineOut, || School:
estat ic
xtmixed logIL6_P3 i.bedtimecat2 sex_n AgeP3 nap_bi_n
i.medical_history_n vediogames Waist_cm3 DineOut, || School:
estat ic

xtmixed IL6_Se_P3 bedtimecat2 sex_n AgeP3 nap_bi_n i.medical_history_n
vediogames DineOut, || School:
estat ic
xtmixed IL6_Se_P3 i.bedtimecat2 sex_n AgeP3 nap_bi_n
i.medical_history_n vediogames Waist_cm3 DineOut, || School:
estat ic
```

```
xtmixed logIL8_P3 i.bedtimecat2 sex_n AgeP3 nap_bi_n
i.medical_history_n vediogames DineOut, || School:
estat ic
xtmixed logIL8_P3 i.bedtimecat2 sex_n AgeP3 nap_bi_n
i.medical_history_n vediogames Waist_cm3 DineOut, || School:
estat ic

xtmixed logIL8_Se_P3 i.bedtimecat2 sex_n AgeP3 nap_bi_n
i.medical_history_n vediogames DineOut, || School:
estat ic
xtmixed logIL8_Se_P3 i.bedtimecat2 sex_n AgeP3 nap_bi_n
i.medical_history_n vediogames Waist_cm3 DineOut, || School:
estat ic

xtmixed logIL10_P3 i.bedtimecat2 sex_n AgeP3 nap_bi_n
i.medical_history_n vediogames DineOut, || School:
estat ic
xtmixed logIL10_P3 i.bedtimecat2 sex_n AgeP3 nap_bi_n
i.medical_history_n vediogames Waist_cm3 DineOut, || School:
estat ic

xtmixed logIL10_Se i.bedtimecat2 sex_n AgeP3 nap_bi_n
i.medical_history_n vediogames DineOut, || School:
estat ic
xtmixed logIL10_Se i.bedtimecat2 sex_n AgeP3 nap_bi_n
i.medical_history_n vediogames Waist_cm3 DineOut, || School:
estat ic

xtmixed VEGF_P3 i.bedtimecat2 sex_n AgeP3 nap_bi_n i.medical_history_n
vediogames DineOut, || School:
estat ic
xtmixed VEGF_P3 i.bedtimecat2 sex_n AgeP3 nap_bi_n i.medical_history_n
vediogames Waist_cm3 DineOut, || School:
estat ic
*Not a confounder
xtmixed logVEGF_Se_P3 i.bedtimecat2 sex_n AgeP3 nap_bi_n
i.medical_history_n vediogames DineOut, || School:
estat ic
xtmixed logVEGF_Se_P3 i.bedtimecat2 sex_n AgeP3 nap_bi_n
i.medical_history_n vediogames Waist_cm3 DineOut, || School:
estat ic
*Not a confounder
xtmixed logMCP1_P3 i.bedtimecat2 sex_n AgeP3 nap_bi_n
i.medical_history_n vediogames DineOut, || School:
estat ic
xtmixed logMCP1_P3 i.bedtimecat2 sex_n AgeP3 nap_bi_n
i.medical_history_n vediogames Waist_cm3 DineOut, || School:
estat ic
*Not confounder
xtmixed logMCP1_Se_P3 i.bedtimecat2 sex_n AgeP3 nap_bi_n
```

```
i.medical_history_n vediogames DineOut, || School:
estat ic
xtmixed logMCP1_Se_P3 i.bedtimecat2 sex_n AgeP3 nap_bi_n
i.medical_history_n vediogames Waist_cm3 DineOut, || School:
estat ic
*Not a confounder
xtmixed logAdiponectin_P3 i.bedtimecat2 sex_n AgeP3 nap_bi_n
i.medical_history_n vediogames DineOut, || School:
estat ic
xtmixed logAdiponectin_P3 i.bedtimecat2 sex_n AgeP3 nap_bi_n
i.medical_history_n vediogames Waist_cm3 DineOut, || School:
estat ic
*A confounder
xtmixed logAdiponectin_Se_P3 i.bedtimecat2 sex_n AgeP3 nap_bi_n
i.medical_history_n vediogames DineOut, || School:
estat ic
xtmixed logAdiponectin_Se_P3 i.bedtimecat2 sex_n AgeP3 nap_bi_n
i.medical_history_n vediogames Waist_cm3 DineOut, || School:
estat ic
* A confounder
xtmixed logInsulin_P3 i.bedtimecat2 sex_n AgeP3 nap_bi_n
i.medical_history_n vediogames DineOut, || School:
estat ic
xtmixed logInsulin_P3 i.bedtimecat2 sex_n AgeP3 nap_bi_n
i.medical_history_n vediogames Waist_cm3 DineOut, || School:
estat ic
* a confounder
xtmixed logInsulin_Se_P3 i.bedtimecat2 sex_n AgeP3 nap_bi_n
i.medical_history_n vediogames DineOut, || School:
estat ic
xtmixed logInsulin_Se_P3 i.bedtimecat2 sex_n AgeP3 nap_bi_n
i.medical_history_n vediogames Waist_cm3 DineOut, || School:
estat ic
* a confounder
xtmixed logLeptin_P3 bedtimecat2 sex_n AgeP3 nap_bi_n
i.medical_history_n vediogames DineOut, || School:
estat ic
xtmixed logLeptin_P3 bedtimecat2 sex_n AgeP3 nap_bi_n
i.medical_history_n vediogames Waist_cm3 DineOut, || School:
estat ic
*not a confounder
xtmixed logLeptin_Se_P3 i.bedtimecat2 sex_n AgeP3 nap_bi_n
i.medical_history_n vediogames DineOut, || School:
estat ic
xtmixed logLeptin_Se_P3 i.bedtimecat2 sex_n AgeP3 nap_bi_n
i.medical_history_n vediogames Waist_cm3 DineOut, || School:
estat ic

*Second: checking for EM:
gen bedwc = bedtimecat2 * Waist_cm3
```

```
xtmixed HSCRPugml bedtimecat2 sex_n AgeP3 nap_bi_n i.medical_history_n
vediogames Waist_cm3 bedwc DineOut, || School:
xtmixed logIL6_P3 bedtimecat2 sex_n AgeP3 nap_bi_n i.medical_history_n
vediogames Waist_cm3 bedwc DineOut, || School:
xtmixed IL6_Se_P3 bedtimecat2 sex_n AgeP3 nap_bi_n i.medical_history_n
vediogames Waist_cm3 bedwc DineOut, || School:
xtmixed logIL8_Se bedtimecat2 sex_n AgeP3 nap_bi_n i.medical_history_n
vediogames Waist_cm3 bedwc DineOut, || School:
xtmixed logIL10_Se bedtimecat2 sex_n AgeP3 nap_bi_n
i.medical_history_n vediogames Waist_cm3 bedwc DineOut, || School:
xtmixed logInsulin_P3 bedtimecat2 sex_n AgeP3 nap_bi_n
i.medical_history_n vediogames Waist_cm3 bedwc DineOut, || School:
xtmixed logInsulin_Se_P3 bedtimecat2 sex_n AgeP3 nap_bi_n
i.medical_history_n vediogames Waist_cm3 bedwc DineOut, || School:
xtmixed logLeptin_Se_P3 bedtimecat2 sex_n AgeP3 nap_bi_n
i.medical_history_n vediogames Waist_cm3 bedwc DineOut, || School:

****************************************************
**********************************************************

*Creating interaction terms between sleep variables
gen weekendboth = Sleep_time_weekend_n * bed_weekend
gen weekdayboth = Sleep_time_weekday_n * bed_hour
gen averageboth = AverageSleepDuration * bed_hour

regress Sleep_time_weekend_n bed_weekend weekendboth sex_n i.School
AgeP3 nap_bi_n i.medical_history_n vediogames DineOut, robust
regress Sleep_time_weekday_n bed_hour weekdayboth sex_n i.School AgeP3
nap_bi_n i.medical_history_n vediogames DineOut, robust
regress AverageSleepDuration bed_hour averageboth sex_n i.School AgeP3
nap_bi_n i.medical_history_n vediogames DineOut, robust
*Now with the biomarkers:
*First: HSCRP
regress HSCRPugml Sleep_time_weekend_n bed_weekend  weekdayboth sex_n
i.School  AgeP3 nap_bi_n i.medical_history_n vediogames DineOut,
robust
regress HSCRPugml Sleep_time_weekday_n bed_hour weekdayboth sex_n
i.School AgeP3 nap_bi_n i.medical_history_n vediogames DineOut, robust
regress HSCRPugml AverageSleepDuration bed_hour averageboth sex_n
i.School AgeP3 nap_bi_n i.medical_history_n vediogames DineOut, robust

*Dr.Redline: Adding SNORE on the association between BMI and Bedtime
logistic OBWHO3_n bed12bin_n snore_bi_n
logit OBWHO3_n bedtime_both2 snore_bi_n
logit OBWHO3_n bedtime_both2 snore_bi_n

**********************************************
Extra Analyses for the thesis:

logit sleepdeptcat  i.bedtimecat2, or
```

```
*the later the bedtime the higher the odds for sleep debt.

logit socialgetlag_bi  i.bedtimecat2, or
*The late rthe bedtime the lowert eh odds for SJL







*********************************************************************
****************************
*********************************************************************
*********************************************************************
************
*************************************PAPER 2
**************************************************
*Diet and Sleep
hist DineOut
hist EatHome

*One-Sample Kolmogorov-Smirnov Test to check for normality of
distribution:
sum DineOut
ksmirnov DineOut = normal((DineOut- -2.63e-17 )/1  )
*Normally distributed

sum EatHome
ksmirnov EatHome = normal((EatHome- -1.15e-17)/ 1)
*Normally distributed

*Nine Categories:
tab Eat_Restaurant
tab Fast_Foods
tab Snacks
tab Physical_Ex
tab Coffee_Tea
tab Milk_All
tab Breakfat
tab Family_meal
tab Eat_TV

*Checking for normality and log transformaitons if not normally
distrbuted.
sum Eat_Restaurant, detail
ksmirnov Eat_Restaurant = normal((Eat_Restaurant- 4.097143 )/
2.645587  )
*not normally
gen logEatRest = log(Eat_Restaurant)
```

```
sum Fast_Foods, detail
ksmirnov Fast_Foods = normal((Fast_Foods- 9.714286  )/4.322735  )
*normal

sum Snacks, detail
ksmirnov Snacks = normal((Snacks- 8.174286)/3.461772  )
*not Normal
gen logSnacks = log(Snacks)

sum Physical_Ex, detail
ksmirnov Physical_Ex = normal((Physical_Ex- 2.474286 )/2.215644  )
*not normal
gen logPhysicalEx = log(Physical_Ex)

sum Coffee_Tea, detail
ksmirnov Coffee_Tea = normal((Coffee_Tea- 2.977143 )/2.118155 )
*Not normal
gen logCoffeeTea= log(Coffee_Tea)

sum Milk_All, detail
ksmirnov Milk_All = normal((Milk_All- 5.471429)/3.164639 )
*Not normal
gen logMilk= log(Milk_All)

sum Breakfat, detail
ksmirnov Breakfat = normal((Breakfat-  3.38)/2.685235 )
*Not normal
gen logBreakfat = log(Breakfat)

sum Family_meal, detail
ksmirnov Family_meal = normal((Family_meal- 5.421203)/2.180822)
*Not normal
gen logFamilyMeal = log(Family_meal)

sum Eat_TV, detail
ksmirnov Eat_TV = normal((Eat_TV- 2.698864 )/2.897135)
*Not normal
gen logEatTV= log(Eat_TV)

*Descriptive statistics:
tabstat Eat_Restaurant,  s(n mean SD) c(s) by(bedtimecat2)
tabstat Fast_Foods,  s(n mean SD) c(s) by(bedtimecat2)
tabstat Snacks,  s(n mean SD) c(s) by(bedtimecat2)
tabstat Physical_Ex,  s(n mean SD) c(s) by(bedtimecat2)
tabstat Coffee_Tea,  s(n mean SD) c(s) by(bedtimecat2)
tabstat Milk_All,  s(n mean SD) c(s) by(bedtimecat2)
tabstat Breakfat,  s(n mean SD) c(s) by(bedtimecat2)
tabstat Family_meal,  s(n mean SD) c(s) by(bedtimecat2)
tabstat Eat_TV,  s(n mean SD) c(s) by(bedtimecat2)
```

```
regress logEatRest i.bedtimecat2
regress Fast_Foods i.bedtimecat2
regress logSnacks i.bedtimecat2
regress logPhysicalEx i.bedtimecat2
regress logCoffeeTea i.bedtimecat2
regress logMilk i.bedtimecat2
regress logBreakfat i.bedtimecat2
regress logFamilyMeal i.bedtimecat2
regress logEatTV i.bedtimecat2


*DineOut
xtmixed DineOut i.bedtimecat2 sex_n AgeP3 nap_bi_n
i.medical_history_n, || School:
*Meidaiton Analysis:
*Vediogames as suspected mediator:
sem (DineOut<-vediogames bedtimecat2) (vediogames<-bedtimecat2),
nocapslatent
sem (AgeP3 sex_n nap_bi_n bedtimecat2 -> DineOut) (AgeP3 sex_n
nap_bi_n bedtimecat2-> vediogames) (vediogames vediogames AgeP3 sex_n
nap_bi_n -> DineOut), vce(bootstrap, reps(5000)) nocapslatent
estat teffects
medsem, indep(bedtimecat2) med(vediogames) dep(DineOut) mcreps(500)
rit rid zlc
estat gof, stats(indices residuals)

*EatHome:
xtmixed EatHome i.bedtimecat2 sex_n AgeP3 nap_bi_n
i.medical_history_n,|| School:
*Meidaiton Analysis:
*Vediogames as suspected mediator:
sem (EatHome<-vediogames bedtimecat2) (vediogames<-bedtimecat2),
nocapslatent
sem (AgeP3 sex_n nap_bi_n bedtimecat2 -> EatHome) (AgeP3 sex_n
nap_bi_n bedtimecat2-> vediogames) (vediogames vediogames AgeP3 sex_n
nap_bi_n -> EatHome), vce(bootstrap, reps(5000)) nocapslatent
estat teffects
medsem, indep(bedtimecat2) med(vediogames) dep(EatHome) mcreps(500)
rit rid zlc
estat gof, stats(indices residuals)
*Vediogames as a confounder:
xtmixed EatHome i.bedtimecat2 sex_n AgeP3 nap_bi_n i.medical_history_n
vediogames,|| School:
```

```
**************************************************

**Other analyses not used in the thesis:
*Diet and bedtime:
ologit bedtimeWDcat EatHome OBWHO3_n sex_n i.medical_history_n School
brant, detail
*Testing Model Assumptions. There are several ways to test the
proportional odds/ parallel
*lines assumption of the ordered logit model. We will start with the
Brant test, which also helps to
*clarify exactly what the assumption is. We will explain the
assumption further when we discuss
*generalized ordered logit (GOLOGIT) models. Appendix A will show
other methods.
*Brant Test. If you have downloaded and installed spost13, you can use
the brant command
*to do Brant’s test of ologit’s parallel regression/ proportional odds
assumption:
*==>The insignificant overall chi-square value (given in the row
labeled All) suggests that ologit’s
*assumptions are met. (Of course, this sample is very small; with
larger samples it is not at all
*unusual to find that the proportional odds assumption is violated.)
brant also gives tests for each
*individual independent variable.
xtmixed bedtimeWDcat EatHome OBWHO3_n sex_n i.medical_history_n, ||
School:

*Mixed effect model for ordinal (Categorical,ordinal) outcome(Mixed
Effect Ordinal Regression)
meologit bedtime123 DineOut sex_n AgeP3 i.medical_history_n vediogames
nap_bi ||School:
meologit bedtime123 EatHome sex_n AgeP3 i.medical_history_n vediogames
nap_bi ||School:

*This command (xtmelogit) for BINARY outcome:
xtmelogit bed12 DineOut OBWHO3_n sex_n i.medical_history_n nap_bi ||
School:
xtmelogit bed12 DineOut sex_n i.medical_history_n nap_bi ||School:

sem (bedtime123<-nap_bi DineOut) (nap_bi<-DineOut), nocapslatent
sem (vediogames DineOut AgeP3 sex_n nap_bi_n DineOut -> bedtime123)
( vediogames DineOut AgeP3 sex_n nap_bi_n DineOut-> nap_bi)
(vediogames DineOut nap_bi AgeP3 sex_n nap_bi_n -> bedtime123),
vce(bootstrap, reps(5000)) nocapslatent
```

```
estat teffects
medsem, indep(DineOut) med( nap_bi ) dep(bedtime123) mcreps(500) rit
rid




**********************************************************************
**********************************************************************
*************
**************************************PAPER
3****************************************************
******************************Obesity, BMI and
bedtime**************************
*Mixed effect linear regression model:
xtmixed BMI3 i.bedtimecat2 sex_n AgeP3 nap_bi_n i.medical_history_n
vediogames DineOut, || School:

*Mixed effect logistic regression model:
xtmelogit OBWHO3_n i.bedtimecat2 sex_n AgeP3 nap_bi_n
i.medical_history_n vediogames DineOut, || School:
xtmelogit, or

*Mixed effect ordinal regression model:
meologit WHOcatP3 i.bedtimecat2 sex_n AgeP3 nap_bi_n
i.medical_history_n vediogames DineOut, || School:
meologit, or
```

```
**********************************************************************
********************************************************************
**********************************************************************
*******************************************************
*********************************Mediation
Analysis****************************
*Barron and Kenny Method:
*First:  HSCRPugml
*1- Estimate the relationship between X on Y (hours since dawn on
degree of wakefulness) -Path “c” must be significantly different from
0; must have a total effect between the IV & DV
regress HSCRPugml bed12bin_n sex_n i.School AgeP3 nap_bi_n
i.medical_history_n vediogames DineOut, robust
xtreg HSCRPugml bed12bin_n sex_n AgeP3 nap_bi_n i.medical_history_n
vediogames DineOut
xtreg HSCRPugml bedtimeWDcat sex_n AgeP3 nap_bi_n i.medical_history_n
vediogames DineOut

*2- Estimate the relationship between X on M (hours since dawn on
coffee consumption) -Path “a” must be significantly different from 0;
IV and mediator must be related.
regress BMI3 bed12bin_n sex_n i.School AgeP3 nap_bi_n
i.medical_history_n vediogames DineOut, robust
xtreg BMI3 bed12bin_n sex_n AgeP3 nap_bi_n i.medical_history_n
vediogames DineOut
xtreg BMI3 bedtimeWDcat sex_n AgeP3 nap_bi_n i.medical_history_n
vediogames DineOut

*3- Estimate the relationship between M on Y controlling for X (coffee
consumption on wakefulness, controlling for hours since dawn) -Path
“b” must be significantly different from 0; mediator and DV must be
related. -The effect of X on Y decreases with the inclusion of M in
the model
regress HSCRPugml BMI3 bed12bin_n sex_n i.School AgeP3 nap_bi_n
i.medical_history_n vediogames DineOut, robust
xtreg HSCRPugml BMI3 bed12bin_n sex_n AgeP3 nap_bi_n
i.medical_history_n vediogames DineOut
xtreg HSCRPugml BMI3 bedtimeWDcat sex_n AgeP3 nap_bi_n
```

```
i.medical_history_n vediogames DineOut

*4- Estimate the relationship between Y on X controlling for M
(wakefulness on hours since dawn, controlling for coffee consumption)
-Should be non-significant and nearly 0.
regress bed12bin_n HSCRPugml BMI3 sex_n i.School AgeP3 nap_bi_n
i.medical_history_n vediogames DineOut, robust
***YES BMI3 is a mediator for the HSCRPugml *****

*Second:IL6 Serum
*1- Estimate the relationship between X on Y (hours since dawn on
degree of wakefulness) -Path “c” must be significantly different from
0; must have a total effect between the IV & DV
regress IL6_Se_P3 bed12bin_n sex_n i.School AgeP3 nap_bi_n
i.medical_history_n vediogames DineOut, robust
*2- Estimate the relationship between X on M (hours since dawn on
coffee consumption) -Path “a” must be significantly different from 0;
IV and mediator must be related.
regress BMI3 bed12bin_n sex_n i.School AgeP3 nap_bi_n
i.medical_history_n vediogames DineOut, robust
*3- Estimate the relationship between M on Y controlling for X (coffee
consumption on wakefulness, controlling for hours since dawn) -Path
“b” must be significantly different from 0; mediator and DV must be
related. -The effect of X on Y decreases with the inclusion of M in
the model
regress IL6_Se_P3 BMI3 bed12bin_n sex_n i.School AgeP3 nap_bi_n
i.medical_history_n vediogames DineOut, robust
*4- Estimate the relationship between Y on X controlling for M
(wakefulness on hours since dawn, controlling for coffee consumption)
-Should be non-significant and nearly 0.
regress bed12bin_n IL6_Se_P3 BMI3 sex_n i.School AgeP3 nap_bi_n
i.medical_history_n vediogames DineOut, robust
***YES BMI3 is a mediator for the IL6 serum *****

*Third:IL6 Saliva ==> log
*1- Estimate the relationship between X on Y (hours since dawn on
degree of wakefulness) -Path “c” must be significantly different from
0; must have a total effect between the IV & DV
regress logIL6_P3 bed12bin_n sex_n i.School AgeP3 nap_bi_n
i.medical_history_n vediogames DineOut, robust
*2- Estimate the relationship between X on M (hours since dawn on
coffee consumption) -Path “a” must be significantly different from 0;
IV and mediator must be related.
regress BMI3 bed12bin_n sex_n i.School AgeP3 nap_bi_n
i.medical_history_n vediogames DineOut, robust
*3- Estimate the relationship between M on Y controlling for X (coffee
consumption on wakefulness, controlling for hours since dawn) -Path
“b” must be significantly different from 0; mediator and DV must be
related. -The effect of X on Y decreases with the inclusion of M in
the model
```

```
regress logIL6_P3 BMI3 bed12bin_n sex_n i.School AgeP3 nap_bi_n
i.medical_history_n vediogames DineOut, robust
*4- Estimate the relationship between Y on X controlling for M
(wakefulness on hours since dawn, controlling for coffee consumption)
-Should be non-significant and nearly 0.
regress bed12bin_n logIL6_P3 BMI3 sex_n i.School AgeP3 nap_bi_n
i.medical_history_n vediogames DineOut, robust
***NO BMI3 is not a mediator for the IL6 saliva****


*Mediation analysis for CRP serum and bedtime WEEKDAY SEM method
*Binary bedtime weekDAYS
sem (HSCRPugml<-BMI3 bed12bin_n) (BMI3<-bed12bin_n), nocapslatent
sem (vediogames DineOut AgeP3 sex_n nap_bi_n bed12bin_n -> HSCRPugml)
( vediogames DineOut AgeP3 sex_n nap_bi_n bed12bin_n-> BMI3)
(vediogames DineOut BMI3 AgeP3 sex_n nap_bi_n -> HSCRPugml),
vce(bootstrap, reps(5000)) nocapslatent
estat teffects
medsem, indep(bed12bin_n) med(BMI3) dep(HSCRPugml) mcreps(500) rit rid

*Categorical bedtime weekDAYS
sem (HSCRPugml<-BMI3 bedtimeWDcat) (BMI3<-bedtimeWDcat), nocapslatent
sem (vediogames DineOut AgeP3 sex_n nap_bi_n bedtimeWDcat ->
HSCRPugml) ( vediogames DineOut AgeP3 sex_n nap_bi_n bedtimeWDcat->
BMI3) (vediogames DineOut BMI3 AgeP3 sex_n nap_bi_n -> HSCRPugml),
vce(bootstrap, reps(5000)) nocapslatent
estat teffects
medsem, indep(bedtimeWDcat) med(BMI3) dep(HSCRPugml) mcreps(500) rit
rid

*Mediation analysis using mixed effect:
ml_mediation, dv(HSCRPugml) iv(bed12bin_n) mv(BMI3) l2id(School)
ml_mediation, dv(HSCRPugml) iv(bedtimeWDcat) mv(BMI3) l2id(School)

* Third: IL6 Slaiva
*Binary bedtime weekdays
sem (logIL6_P3<-BMI3 bed12bin_n) (BMI3<-bed12bin_n), nocapslatent
sem (vediogames DineOut AgeP3 sex_n nap_bi_n bed12bin_n -> logIL6_P3)
( vediogames DineOut AgeP3 sex_n nap_bi_n bed12bin_n-> BMI3)
(vediogames DineOut BMI3 AgeP3 sex_n nap_bi_n -> logIL6_P3),
vce(bootstrap, reps(5000)) nocapslatent
estat teffects
medsem, indep(bed12bin_n) med(BMI3) dep(logIL6_P3) mcreps(500) rit rid
zlc

*Categorical bedtime weekdays
sem (logIL6_P3<-BMI3 bedtimeWDcat) (BMI3<-bedtimeWDcat), nocapslatent
sem (vediogames DineOut AgeP3 sex_n nap_bi_n bedtimeWDcat ->
logIL6_P3) ( vediogames DineOut AgeP3 sex_n nap_bi_n bedtimeWDcat->
BMI3) (vediogames DineOut BMI3 AgeP3 sex_n nap_bi_n -> logIL6_P3),
```

```
vce(bootstrap, reps(5000)) nocapslatent
estat teffects
medsem, indep(bedtimeWDcat) med(BMI3) dep(logIL6_P3) mcreps(500) rit
rid zlc

*Mediation analysis using mixed effect:
ml_mediation, dv(logIL6_P3) iv(bed12bin_n) mv(BMI3) l2id(School)
ml_mediation, dv(logIL6_P3) iv(bedtimeWDcat) mv(BMI3) l2id(School)


* Fourth: IL8 Slaiva
*Binary bedtime weekdays
sem (logIL8_P3 <-BMI3 bed12bin_n) (BMI3<-bed12bin_n), nocapslatent
sem (vediogames DineOut AgeP3 sex_n nap_bi_n bed12bin_n -> logIL8_P3 )
( vediogames DineOut AgeP3 sex_n nap_bi_n bed12bin_n-> BMI3)
(vediogames DineOut BMI3 AgeP3 sex_n nap_bi_n -> logIL8_P3 ),
vce(bootstrap, reps(5000)) nocapslatent
estat teffects
medsem, indep(bed12bin_n) med(BMI3) dep(logIL8_P3 ) mcreps(500) rit
rid zlc

*Categorical bedtime weekdays
sem (logIL8_P3 <-BMI3 bedtimeWDcat) (BMI3<-bedtimeWDcat), nocapslatent
sem (vediogames DineOut AgeP3 sex_n nap_bi_n bedtimeWDcat ->
logIL8_P3 ) ( vediogames DineOut AgeP3 sex_n nap_bi_n bedtimeWDcat->
BMI3) (vediogames DineOut BMI3 AgeP3 sex_n nap_bi_n -> logIL8_P3 ),
vce(bootstrap, reps(5000)) nocapslatent
estat teffects
medsem, indep(bedtimeWDcat) med(BMI3) dep(logIL8_P3 ) mcreps(500) rit
rid zlc

*Mediation analysis using mixed effect:
ml_mediation, dv(logIL8_P3) iv(bed12bin_n) mv(BMI3) l2id(School)
ml_mediation, dv(logIL8_P3 ) iv(bedtimeWDcat) mv(BMI3) l2id(School)


* Fifth: IL8 Serum
*Binary bedtime weekdays
sem (logIL8_Se_P3<-BMI3 bed12bin_n) (BMI3<-bed12bin_n), nocapslatent
sem (vediogames DineOut AgeP3 sex_n nap_bi_n bed12bin_n ->
logIL8_Se_P3) ( vediogames DineOut AgeP3 sex_n nap_bi_n bed12bin_n->
BMI3) (vediogames DineOut BMI3 AgeP3 sex_n nap_bi_n -> logIL8_Se_P3),
vce(bootstrap, reps(5000)) nocapslatent
estat teffects
medsem, indep(bed12bin_n) med(BMI3) dep(logIL8_Se_P3) mcreps(500) rit
rid zlc

*Categorical bedtime weekdays
sem (logIL8_Se_P3<-BMI3 bedtimeWDcat) (BMI3<-bedtimeWDcat),
nocapslatent
```

```
sem (vediogames DineOut AgeP3 sex_n nap_bi_n bedtimeWDcat ->
logIL8_Se_P3) ( vediogames DineOut AgeP3 sex_n nap_bi_n bedtimeWDcat->
BMI3) (vediogames DineOut BMI3 AgeP3 sex_n nap_bi_n -> logIL8_Se_P3),
vce(bootstrap, reps(5000)) nocapslatent
estat teffects
medsem, indep(bedtimeWDcat) med(BMI3) dep(logIL8_Se_P3) mcreps(500)
rit rid zlc

*Mediation analysis using mixed effect:
ml_mediation, dv(logIL8_Se_P3) iv(bed12bin_n) mv(BMI3) l2id(School)
ml_mediation, dv(logIL8_Se_P3) iv(bedtimeWDcat) mv(BMI3) l2id(School)
**********************************************************************
************
This is the mediator supposed to use with this average cat bedtime
variable but not used.

************************************ BMI as a possible
mediator*********************************
Average bedtime used in this study
* 1: CRP Saliva
*Categorical Average bedtime:
sem (logCRP_P3<-BMI3 bedtimecat2) (BMI3<-bedtimecat2), nocapslatent
sem (vediogames DineOut AgeP3 sex_n nap_bi_n bedtimecat2 -> logCRP_P3)
( vediogames DineOut AgeP3 sex_n nap_bi_n bedtimecat2-> BMI3)
(vediogames DineOut BMI3 AgeP3 sex_n nap_bi_n -> logCRP_P3),
vce(bootstrap, reps(5000)) nocapslatent
estat teffects
medsem, indep(bedtimecat2) med(BMI3) dep(logCRP_P3) mcreps(500) rit
rid
estat gof, stats(indices residuals)
* BMI not mediator (indirect effect is not stat significant)

* 2: HSCRP
*Categorical Average bedtime:
sem (HSCRPugml<-BMI3 bedtimecat2) (BMI3<-bedtimecat2), nocapslatent
sem (vediogames DineOut AgeP3 sex_n nap_bi_n bedtimecat2 -> HSCRPugml)
( vediogames DineOut AgeP3 sex_n nap_bi_n bedtimecat2-> BMI3)
(vediogames DineOut BMI3 AgeP3 sex_n nap_bi_n -> HSCRPugml),
vce(bootstrap, reps(5000)) nocapslatent
estat teffects
medsem, indep(bedtimecat2) med(BMI3) dep(HSCRPugml) mcreps(500) rit
rid
estat gof, stats(indices residuals)
* BMI is a mediator for the association between

* 3- IL6 Serum
*Categorical Average bedtime:
sem (IL6_Se_P3<-BMI3 bedtime123) (BMI3<-bedtime123), nocapslatent
sem (vediogames DineOut AgeP3 sex_n nap_bi_n bedtime123 -> IL6_Se_P3)
( vediogames DineOut AgeP3 sex_n nap_bi_n bedtime123-> BMI3)
```

```
(vediogames DineOut BMI3 AgeP3 sex_n nap_bi_n -> IL6_Se_P3),
vce(bootstrap, reps(5000)) nocapslatent
estat teffects
medsem, indep(bedtime123) med(BMI3) dep(IL6_Se_P3) mcreps(500) rit rid
estat gof, stats(indices residuals)

* 4- IL-6 saliva
*Categorical Average bedtime:
sem (logIL6_P3<-BMI3 bedtime123) (BMI3<-bedtime123), nocapslatent
sem (vediogames DineOut AgeP3 sex_n nap_bi_n bedtime123 -> logIL6_P3)
( vediogames DineOut AgeP3 sex_n nap_bi_n bedtime123-> BMI3)
(vediogames DineOut BMI3 AgeP3 sex_n nap_bi_n -> logIL6_P3),
vce(bootstrap, reps(5000)) nocapslatent
estat teffects
medsem, indep(bedtime123) med(BMI3) dep(logIL6_P3) mcreps(500) rit rid
estat gof, stats(indices residuals)

* 5- IL_8 serum
sem (logIL8_Se_P3<-BMI3 bedtime123) (BMI3<-bedtime123), nocapslatent
sem (vediogames DineOut AgeP3 sex_n nap_bi_n bedtime123 ->
logIL8_Se_P3) ( vediogames DineOut AgeP3 sex_n nap_bi_n bedtime123->
BMI3) (vediogames DineOut BMI3 AgeP3 sex_n nap_bi_n -> logIL8_Se_P3),
vce(bootstrap, reps(5000)) nocapslatent
estat teffects
medsem, indep(bedtime123) med(BMI3) dep(logIL8_Se_P3) mcreps(500) rit
rid
estat gof, stats(indices residuals)

* 6- IL_10 serum
sem (logIL10_Se <-BMI3 bedtime123) (BMI3<-bedtime123), nocapslatent
sem (vediogames DineOut AgeP3 sex_n nap_bi_n bedtime123 ->
logIL10_Se ) ( vediogames DineOut AgeP3 sex_n nap_bi_n bedtime123->
BMI3) (vediogames DineOut BMI3 AgeP3 sex_n nap_bi_n -> logIL10_Se),
vce(bootstrap, reps(5000)) nocapslatent
estat teffects
medsem, indep(bedtime123) med(BMI3) dep(logIL10_Se ) mcreps(500) rit
rid
estat gof, stats(indices residuals)
**********************************************************************
******************
********************************* Waist Circumference as a possible
mediator************
* First: HSCRP
sem (HSCRPugml<-Waist_cm3 bed12bin_n) (Waist_cm3<-bed12bin_n),
nocapslatent
sem (vediogames DineOut AgeP3 sex_n nap_bi_n bed12bin_n -> HSCRPugml)
( vediogames DineOut AgeP3 sex_n nap_bi_n bed12bin_n-> Waist_cm3)
(vediogames DineOut Waist_cm3 AgeP3 sex_n nap_bi_n -> HSCRPugml),
vce(bootstrap, reps(5000)) nocapslatent
estat teffects
```

```
medsem, indep(bed12bin_n) med(Waist_cm3) dep(HSCRPugml) mcreps(500)
rit rid
* WC is not a mediator for the association

* Second: IL6 Serum
sem (IL6_Se_P3<-Waist_cm3 bed12bin_n) (Waist_cm3<-bed12bin_n),
nocapslatent
sem (vediogames DineOut AgeP3 sex_n nap_bi_n bed12bin_n -> IL6_Se_P3)
( vediogames DineOut AgeP3 sex_n nap_bi_n bed12bin_n-> Waist_cm3)
(vediogames DineOut Waist_cm3 AgeP3 sex_n nap_bi_n -> IL6_Se_P3),
vce(bootstrap, reps(5000)) nocapslatent
estat teffects
medsem, indep(bed12bin_n) med(Waist_cm3) dep(IL6_Se_P3) mcreps(500)
rit rid zlc
* WC is not a mediator for the association

* Third: IL6 Slaiva
sem (logIL6_P3<-Waist_cm3 bed12bin_n) (Waist_cm3<-bed12bin_n),
nocapslatent
sem (vediogames DineOut AgeP3 sex_n nap_bi_n bed12bin_n -> logIL6_P3)
( vediogames DineOut AgeP3 sex_n nap_bi_n bed12bin_n-> Waist_cm3)
(vediogames DineOut Waist_cm3 AgeP3 sex_n nap_bi_n -> logIL6_P3),
vce(bootstrap, reps(5000)) nocapslatent
estat teffects
medsem, indep(bed12bin_n) med(Waist_cm3) dep(logIL6_P3) mcreps(500)
rit rid zlc
* WC is not a mediator for the association

*IL-10 serum
sem (logIL10_Se<-Waist_cm3 bed12bin_n) (Waist_cm3<-bed12bin_n),
nocapslatent
sem (vediogames DineOut AgeP3 sex_n nap_bi_n bed12bin_n -> logIL10_Se)
( vediogames DineOut AgeP3 sex_n nap_bi_n bed12bin_n-> Waist_cm3)
(vediogames DineOut Waist_cm3 AgeP3 sex_n nap_bi_n -> logIL10_Se),
vce(bootstrap, reps(5000)) nocapslatent
estat teffects
medsem, indep(bed12bin_n) med(Waist_cm3) dep(logIL10_Se) mcreps(500)
rit rid zlc

*Insulin saliva:
sem (logInsulin_P3<-Waist_cm3 bed12bin_n) (Waist_cm3<-bed12bin_n),
nocapslatent
sem (vediogames DineOut AgeP3 sex_n nap_bi_n bed12bin_n ->
logInsulin_P3) ( vediogames DineOut AgeP3 sex_n nap_bi_n bed12bin_n->
Waist_cm3) (vediogames DineOut Waist_cm3 AgeP3 sex_n nap_bi_n ->
logInsulin_P3), vce(bootstrap, reps(5000)) nocapslatent
estat teffects
medsem, indep(bed12bin_n) med(Waist_cm3) dep(logInsulin_P3)
mcreps(500) rit rid zlc
*********************************** Nap as a possible
```

```
mediator*************************
* First: HSCRP
sem (HSCRPugml<-nap_bi_n bed12bin_n) (nap_bi_n<-bed12bin_n),
nocapslatent
sem (vediogames DineOut AgeP3 sex_n bed12bin_n -> HSCRPugml)
( vediogames DineOut AgeP3 sex_n bed12bin_n-> nap_bi_n) (vediogames
DineOut nap_bi_n AgeP3 sex_n -> HSCRPugml), vce(bootstrap, reps(5000))
nocapslatent
estat teffects
medsem, indep(bed12bin_n) med(nap_bi_n) dep(HSCRPugml) mcreps(500) rit
rid
* NAP is not a mediator for the association

* Second: IL6 Serum
sem (IL6_Se_P3<-nap_bi_n bed12bin_n) (nap_bi_n<-bed12bin_n),
nocapslatent
sem (vediogames DineOut AgeP3 sex_n bed12bin_n -> IL6_Se_P3)
( vediogames DineOut AgeP3 sex_n bed12bin_n-> nap_bi_n) (vediogames
DineOut nap_bi_n AgeP3 sex_n -> IL6_Se_P3), vce(bootstrap, reps(5000))
nocapslatent
estat teffects
medsem, indep(bed12bin_n) med(nap_bi_n) dep(IL6_Se_P3) mcreps(500) rit
rid zlc
* NAP is not a mediator for the association

* Third: IL6 Slaiva
sem (logIL6_P3<-nap_bi_n bed12bin_n) (nap_bi_n<-bed12bin_n),
nocapslatent
sem (vediogames DineOut AgeP3 sex_n bed12bin_n -> logIL6_P3)
( vediogames DineOut AgeP3 sex_n bed12bin_n-> nap_bi_n) (vediogames
DineOut nap_bi_n AgeP3 sex_n -> logIL6_P3), vce(bootstrap, reps(5000))
nocapslatent
estat teffects
medsem, indep(bed12bin_n) med(nap_bi_n) dep(logIL6_P3) mcreps(500) rit
rid zlc
* NAP is not a mediator for the association

* Second: IL10 Serum
sem (IL6_Se_P3<-nap_bi_n bed12bin_n) (nap_bi_n<-bed12bin_n),
nocapslatent
sem (vediogames DineOut AgeP3 sex_n bed12bin_n -> IL6_Se_P3)
( vediogames DineOut AgeP3 sex_n bed12bin_n-> nap_bi_n) (vediogames
DineOut nap_bi_n AgeP3 sex_n -> IL6_Se_P3), vce(bootstrap, reps(5000))
nocapslatent
estat teffects
medsem, indep(bed12bin_n) med(nap_bi_n) dep(IL6_Se_P3) mcreps(500) rit
rid zlc
* NAP is not a mediator for the association

* Third: Insulin Slaiva
```

```
sem (logIL6_P3<-nap_bi_n bed12bin_n) (nap_bi_n<-bed12bin_n),
nocapslatent
sem (vediogames DineOut AgeP3 sex_n bed12bin_n -> logIL6_P3)
( vediogames DineOut AgeP3 sex_n bed12bin_n-> nap_bi_n) (vediogames
DineOut nap_bi_n AgeP3 sex_n -> logIL6_P3), vce(bootstrap, reps(5000))
nocapslatent
estat teffects
medsem, indep(bed12bin_n) med(nap_bi_n) dep(logIL6_P3) mcreps(500) rit
rid zlc
* NAP is not a mediator for the association
**********************************************************************
*************************************
*Second check for effect modification:

**********************************************************************
*****************************************
**************************EFFECT
MODIFIERS**************************************

*****************OBWHO3_n as suspected effect modifier for with
Sleep_time_weekday*******************************
*generating interaction term:
gen intSLEP12OBE = bed12bin_n * OBWHO3_n
*Third: IL6 saliva
regress logIL6_P3 bed12bin_n sex_n i.School AgeP3 nap_bi_n
i.medical_history_n vediogames DineOut OBWHO3_n c.intSLEP12OBE, robust

*Another way to generate interactin term
regress CRP_P3 bed12bin_n##OBWHO3_n
regress IL6_Se_P3 bed12bin_n##OBWHO3_n
regress logIL6_P3 bed12bin_n##OBWHO3_n
*OBWHO3_n is not an effect modifier for IL6 saliva with bed12bin_n
because the interaction term was not statistically significant
**********************************************************************
***********
*Dr. Redline:
*Consider the analysis with and without the NAP variable: (Abeer ==>
suspected confounding variable)
* First: HSCRP
regress HSCRPugml Sleep_time_weekday sex_n i.School AgeP3 nap_bi_n
i.medical_history_n vediogames DineOut, robust
regress HSCRPugml Sleep_time_weekday sex_n i.School AgeP3
i.medical_history_n vediogames DineOut, robust
* when applying the 10% rule we found that NAP is a confounding
variable (12%)
regress HSCRPugml bed12bin_n sex_n i.School AgeP3 nap_bi_n
i.medical_history_n vediogames DineOut, robust
regress HSCRPugml bed12bin_n sex_n i.School AgeP3 i.medical_history_n
vediogames DineOut, robust
* when applying the 10% rule we found that NAP is a confounding
```

```
variable (15%)

* Second: IL6 Serum
regress logIL6_P3 Sleep_time_weekday sex_n i.School AgeP3 nap_bi_n
i.medical_history_n vediogames DineOut, robust
regress logIL6_P3 Sleep_time_weekday sex_n i.School AgeP3
i.medical_history_n vediogames DineOut, robust
* when applying the 10% rule we found that NAP is NOT a confounding
variable (5.7% but when we look at the definition of conofunder we can
say that NAP is a confounding variable)
regress IL6_Se_P3 bed12bin_n sex_n i.School AgeP3 nap_bi_n
i.medical_history_n vediogames DineOut, robust
regress IL6_Se_P3 bed12bin_n sex_n i.School AgeP3 i.medical_history_n
vediogames DineOut, robust
* when applying the 10% rule we found that NAP is a confounding
variable (14.5%).

* Third: IL6 Slaiva
regress logIL6_P3 Sleep_time_weekday sex_n i.School AgeP3 nap_bi_n
i.medical_history_n vediogames DineOut, robust
regress logIL6_P3 Sleep_time_weekday sex_n i.School AgeP3
i.medical_history_n vediogames DineOut, robust
* when applying the 10% rule we found that NAP is NOT a confounding
variable (5.7% but when we look at the definition of conofunder we can
say that NAP is a confounding variable)
regress logIL6_P3 bed12bin_n sex_n i.School AgeP3 nap_bi_n
i.medical_history_n vediogames DineOut, robust
regress logIL6_P3 bed12bin_n sex_n i.School AgeP3 i.medical_history_n
vediogames DineOut, robust
* when applying the 10% rule we found that NAP is a confounding
variable (11.6%)

**********************************************************************
***********************************

xtmixed logIL10 AverageSleepDuration sex_n AgeP3 nap_bi_n
i.medical_history_n vediogames DineOut, || School:
xtmixed logIL10 Sleep_time_weekday_n sex_n AgeP3 nap_bi_n
i.medical_history_n vediogames DineOut, || School:
xtmixed logIL10 Sleep_time_weekend_n sex_n AgeP3 nap_bi_n
i.medical_history_n vediogames DineOut, || School:

xtmixed logIL10 bedtime_both2 sex_n AgeP3 nap_bi_n i.medical_history_n
vediogames DineOut, || School:
xtmixed logIL10 bed_hour sex_n AgeP3 nap_bi_n i.medical_history_n
vediogames DineOut, || School:
xtmixed logIL10 bed_weekend sex_n AgeP3 nap_bi_n i.medical_history_n
vediogames DineOut, || School:

xtmixed logIL10 bed12 sex_n AgeP3 nap_bi_n i.medical_history_n
```

```
vediogames DineOut, || School:
xtmixed logIL10 bed12bin_n sex_n AgeP3 nap_bi_n i.medical_history_n
vediogames DineOut, || School:
xtmixed logIL10 bed12_weekend_bi sex_n AgeP3 nap_bi_n
i.medical_history_n vediogames DineOut, || School:

xtmixed logIL10 i.bedtime123 sex_n AgeP3 nap_bi_n i.medical_history_n
vediogames DineOut, || School:
xtmixed logIL10 i.bedtimeWDcat sex_n AgeP3 nap_bi_n
i.medical_history_n vediogames DineOut, || School:
xtmixed logIL10 i.bedtimeWEcat sex_n AgeP3 nap_bi_n
i.medical_history_n vediogames DineOut, || School:

xtmixed logIL10 i.timeandduration sex_n AgeP3 nap_bi_n
i.medical_history_n vediogames DineOut, || School:

xtmixed logIL10 sleepdept sex_n AgeP3 nap_bi_n i.medical_history_n
vediogames DineOut, || School:
xtmixed logIL10 i.sleepdeptcat sex_n AgeP3 nap_bi_n
i.medical_history_n vediogames DineOut, || School:
xtmixed logIL10 i.sleepdeptsevere sex_n AgeP3 nap_bi_n
i.medical_history_n vediogames DineOut, || School:

xtmixed logIL10 social_getlag sex_n AgeP3 nap_bi_n i.medical_history_n
vediogames DineOut, || School:
xtmixed logIL10 i.socialgetlag_bi sex_n AgeP3 nap_bi_n
i.medical_history_n vediogames DineOut, || School:

**********************************************************************
***********************************
*RANDOM EFFECT MODELS for the biomarkers saliva and serum
*RANDOM EFFECT MODEL
xtreg logInsulin_P3 AverageSleepDuration sex_n AgeP3 nap_bi_n
i.medical_history_n vediogames DineOut
xtreg logInsulin_P3 Sleep_time_weekday_n sex_n AgeP3 nap_bi_n
i.medical_history_n vediogames DineOut
xtreg logInsulin_P3 Sleep_time_weekend_n sex_n AgeP3 nap_bi_n
i.medical_history_n vediogames DineOut

xtreg logInsulin_P3 bedtime_both2 sex_n AgeP3 nap_bi_n
i.medical_history_n vediogames DineOut
xtreg logInsulin_P3 bed_hour sex_n AgeP3 nap_bi_n i.medical_history_n
vediogames DineOut
xtreg logInsulin_P3 bed_weekend sex_n AgeP3 nap_bi_n
i.medical_history_n vediogames DineOut

xtreg logInsulin_P3 bed12 sex_n AgeP3 nap_bi_n i.medical_history_n
vediogames DineOut
xtreg logInsulin_P3 bed12bin_n sex_n AgeP3 nap_bi_n
i.medical_history_n vediogames DineOut
```

```
xtreg logInsulin_P3 bed12_weekend_bi sex_n AgeP3 nap_bi_n
i.medical_history_n vediogames DineOut

xtreg logInsulin_P3 i.bedtime123 sex_n AgeP3 nap_bi_n
i.medical_history_n vediogames DineOut
xtreg logInsulin_P3 i.bedtimeWDcat sex_n AgeP3 nap_bi_n
i.medical_history_n vediogames DineOut
xtreg logInsulin_P3 i.bedtimeWEcat sex_n AgeP3 nap_bi_n
i.medical_history_n vediogames DineOut

xtreg logInsulin_P3 i.timeandduration sex_n AgeP3 nap_bi_n
i.medical_history_n vediogames DineOut

xtreg logInsulin_P3 sleepdept sex_n AgeP3 nap_bi_n i.medical_history_n
vediogames DineOut
xtreg logInsulin_P3 i.sleepdeptcat sex_n AgeP3 nap_bi_n
i.medical_history_n vediogames DineOut
xtreg logInsulin_P3 i.sleepdeptsevere sex_n AgeP3 nap_bi_n
i.medical_history_n vediogames DineOut

xtreg logInsulin_P3 social_getlag sex_n AgeP3 nap_bi_n
i.medical_history_n vediogames DineOut
xtreg logInsulin_P3 i.socialgetlag_bi sex_n AgeP3 nap_bi_n
i.medical_history_n vediogames DineOut

*RANDOM EFFECT MODEL
xtreg logInsulin_Se_P3 AverageSleepDuration sex_n AgeP3 nap_bi_n
i.medical_history_n vediogames DineOut
xtreg logInsulin_Se_P3 Sleep_time_weekday_n sex_n AgeP3 nap_bi_n
i.medical_history_n vediogames DineOut
xtreg logInsulin_Se_P3 Sleep_time_weekend_n sex_n AgeP3 nap_bi_n
i.medical_history_n vediogames DineOut

xtreg logInsulin_Se_P3 bedtime_both2 sex_n AgeP3 nap_bi_n
i.medical_history_n vediogames DineOut
xtreg logInsulin_Se_P3 bed_hour sex_n AgeP3 nap_bi_n
i.medical_history_n vediogames DineOut
xtreg logInsulin_Se_P3 bed_weekend sex_n AgeP3 nap_bi_n
i.medical_history_n vediogames DineOut

xtreg logInsulin_Se_P3 bed12 sex_n AgeP3 nap_bi_n i.medical_history_n
vediogames DineOut
xtreg logInsulin_Se_P3 bed12bin_n sex_n AgeP3 nap_bi_n
i.medical_history_n vediogames DineOut
xtreg logInsulin_Se_P3 bed12_weekend_bi sex_n AgeP3 nap_bi_n
i.medical_history_n vediogames DineOut

xtreg logInsulin_Se_P3 i.bedtime123 sex_n AgeP3 nap_bi_n
i.medical_history_n vediogames DineOut
xtreg logInsulin_Se_P3 i.bedtimeWDcat sex_n AgeP3 nap_bi_n
```

```
i.medical_history_n vediogames DineOut
xtreg logInsulin_Se_P3 i.bedtimeWEcat sex_n AgeP3 nap_bi_n
i.medical_history_n vediogames DineOut

xtreg logInsulin_Se_P3 i.timeandduration sex_n AgeP3 nap_bi_n
i.medical_history_n vediogames DineOut

xtreg logInsulin_Se_P3 sleepdept sex_n AgeP3 nap_bi_n
i.medical_history_n vediogames DineOut
xtreg logInsulin_Se_P3 i.sleepdeptcat sex_n AgeP3 nap_bi_n
i.medical_history_n vediogames DineOut
xtreg logInsulin_Se_P3 i.sleepdeptsevere sex_n AgeP3 nap_bi_n
i.medical_history_n vediogames DineOut

xtreg logInsulin_Se_P3 social_getlag sex_n AgeP3 nap_bi_n
i.medical_history_n vediogames DineOut
xtreg logInsulin_Se_P3 i.socialgetlag_bi sex_n AgeP3 nap_bi_n
i.medical_history_n vediogames DineOut

*RANDOM EFFECT MODEL
xtreg logMCP1_P3  AverageSleepDuration sex_n AgeP3 nap_bi_n
i.medical_history_n vediogames DineOut
xtreg logMCP1_P3  Sleep_time_weekday_n sex_n AgeP3 nap_bi_n
i.medical_history_n vediogames DineOut
xtreg logMCP1_P3  Sleep_time_weekend_n sex_n AgeP3 nap_bi_n
i.medical_history_n vediogames DineOut

xtreg logMCP1_P3  bedtime_both2 sex_n AgeP3 nap_bi_n
i.medical_history_n vediogames DineOut
xtreg logMCP1_P3  bed_hour sex_n AgeP3 nap_bi_n i.medical_history_n
vediogames DineOut
xtreg logMCP1_P3  bed_weekend sex_n AgeP3 nap_bi_n i.medical_history_n
vediogames DineOut

xtreg logMCP1_P3  bed12 sex_n AgeP3 nap_bi_n i.medical_history_n
vediogames DineOut
xtreg logMCP1_P3  bed12bin_n sex_n AgeP3 nap_bi_n i.medical_history_n
vediogames DineOut
xtreg logMCP1_P3  bed12_weekend_bi sex_n AgeP3 nap_bi_n
i.medical_history_n vediogames DineOut

xtreg logMCP1_P3  i.bedtime123 sex_n AgeP3 nap_bi_n
i.medical_history_n vediogames DineOut
xtreg logMCP1_P3  i.bedtimeWDcat sex_n AgeP3 nap_bi_n
i.medical_history_n vediogames DineOut
xtreg logMCP1_P3  i.bedtimeWEcat sex_n AgeP3 nap_bi_n
i.medical_history_n vediogames DineOut

xtreg logMCP1_P3  i.timeandduration sex_n AgeP3 nap_bi_n
i.medical_history_n vediogames DineOut
```

```
xtreg logMCP1_P3 sleepdept sex_n AgeP3 nap_bi_n i.medical_history_n
vediogames DineOut
xtreg logMCP1_P3 i.sleepdeptcat sex_n AgeP3 nap_bi_n
i.medical_history_n vediogames DineOut
xtreg logMCP1_P3 i.sleepdeptsevere sex_n AgeP3 nap_bi_n
i.medical_history_n vediogames DineOut

xtreg logMCP1_P3 social_getlag sex_n AgeP3 nap_bi_n
i.medical_history_n vediogames DineOut
xtreg logMCP1_P3 i.socialgetlag_bi sex_n AgeP3 nap_bi_n
i.medical_history_n vediogames DineOut

*RANDOM EFFECT MODEL
xtreg logMCP1_Se_P3 AverageSleepDuration sex_n AgeP3 nap_bi_n
i.medical_history_n vediogames DineOut
xtreg logMCP1_Se_P3 Sleep_time_weekday_n sex_n AgeP3 nap_bi_n
i.medical_history_n vediogames DineOut
xtreg logMCP1_Se_P3 Sleep_time_weekend_n sex_n AgeP3 nap_bi_n
i.medical_history_n vediogames DineOut

xtreg logMCP1_Se_P3 bedtime_both2 sex_n AgeP3 nap_bi_n
i.medical_history_n vediogames DineOut
xtreg logMCP1_Se_P3 bed_hour sex_n AgeP3 nap_bi_n i.medical_history_n
vediogames DineOut
xtreg logMCP1_Se_P3 bed_weekend sex_n AgeP3 nap_bi_n
i.medical_history_n vediogames DineOut

xtreg logMCP1_Se_P3 bed12 sex_n AgeP3 nap_bi_n i.medical_history_n
vediogames DineOut
xtreg logMCP1_Se_P3 bed12bin_n sex_n AgeP3 nap_bi_n
i.medical_history_n vediogames DineOut
xtreg logMCP1_Se_P3 bed12_weekend_bi sex_n AgeP3 nap_bi_n
i.medical_history_n vediogames DineOut

xtreg logMCP1_Se_P3 i.bedtime123 sex_n AgeP3 nap_bi_n
i.medical_history_n vediogames DineOut
xtreg logMCP1_Se_P3 i.bedtimeWDcat sex_n AgeP3 nap_bi_n
i.medical_history_n vediogames DineOut
xtreg logMCP1_Se_P3 i.bedtimeWEcat sex_n AgeP3 nap_bi_n
i.medical_history_n vediogames DineOut

xtreg logMCP1_Se_P3 i.timeandduration sex_n AgeP3 nap_bi_n
i.medical_history_n vediogames DineOut

xtreg logMCP1_Se_P3 sleepdept sex_n AgeP3 nap_bi_n i.medical_history_n
vediogames DineOut
xtreg logMCP1_Se_P3 i.sleepdeptcat sex_n AgeP3 nap_bi_n
i.medical_history_n vediogames DineOut
xtreg logMCP1_Se_P3 i.sleepdeptsevere sex_n AgeP3 nap_bi_n
```

```
i.medical_history_n vediogames DineOut

xtreg logMCP1_Se_P3 social_getlag sex_n AgeP3 nap_bi_n
i.medical_history_n vediogames DineOut
xtreg logMCP1_Se_P3 i.socialgetlag_bi sex_n AgeP3 nap_bi_n
i.medical_history_n vediogames DineOut

*RANDOM EFFECT MODEL
xtreg logLeptin_P3  AverageSleepDuration sex_n AgeP3 nap_bi_n
i.medical_history_n vediogames DineOut
xtreg logLeptin_P3  Sleep_time_weekday_n sex_n AgeP3 nap_bi_n
i.medical_history_n vediogames DineOut
xtreg logLeptin_P3  Sleep_time_weekend_n sex_n AgeP3 nap_bi_n
i.medical_history_n vediogames DineOut

xtreg logLeptin_P3  bedtime_both2 sex_n AgeP3 nap_bi_n
i.medical_history_n vediogames DineOut
xtreg logLeptin_P3  bed_hour sex_n AgeP3 nap_bi_n i.medical_history_n
vediogames DineOut
xtreg logLeptin_P3  bed_weekend sex_n AgeP3 nap_bi_n
i.medical_history_n vediogames DineOut

xtreg logLeptin_P3  bed12 sex_n AgeP3 nap_bi_n i.medical_history_n
vediogames DineOut
xtreg logLeptin_P3  bed12bin_n sex_n AgeP3 nap_bi_n
i.medical_history_n vediogames DineOut
xtreg logLeptin_P3  bed12_weekend_bi sex_n AgeP3 nap_bi_n
i.medical_history_n vediogames DineOut

xtreg logLeptin_P3  i.bedtime123 sex_n AgeP3 nap_bi_n
i.medical_history_n vediogames DineOut
xtreg logLeptin_P3  i.bedtimeWDcat sex_n AgeP3 nap_bi_n
i.medical_history_n vediogames DineOut
xtreg logLeptin_P3  i.bedtimeWEcat sex_n AgeP3 nap_bi_n
i.medical_history_n vediogames DineOut

xtreg logLeptin_P3 i.timeandduration sex_n AgeP3 nap_bi_n
i.medical_history_n vediogames DineOut

xtreg logLeptin_P3 sleepdept sex_n AgeP3 nap_bi_n i.medical_history_n
vediogames DineOut
xtreg logLeptin_P3 i.sleepdeptcat sex_n AgeP3 nap_bi_n
i.medical_history_n vediogames DineOut
xtreg logLeptin_P3 i.sleepdeptsevere sex_n AgeP3 nap_bi_n
i.medical_history_n vediogames DineOut

xtreg logLeptin_P3 social_getlag sex_n AgeP3 nap_bi_n
i.medical_history_n vediogames DineOut
xtreg logLeptin_P3 i.socialgetlag_bi sex_n AgeP3 nap_bi_n
i.medical_history_n vediogames DineOut
```

```
*RANDOM EFFECT MODEL
xtreg logLeptin_Se_P3 AverageSleepDuration sex_n AgeP3 nap_bi_n
i.medical_history_n vediogames DineOut
xtreg logLeptin_Se_P3 Sleep_time_weekday_n sex_n AgeP3 nap_bi_n
i.medical_history_n vediogames DineOut
xtreg logLeptin_Se_P3 Sleep_time_weekend_n sex_n AgeP3 nap_bi_n
i.medical_history_n vediogames DineOut

xtreg logLeptin_Se_P3 bedtime_both2 sex_n AgeP3 nap_bi_n
i.medical_history_n vediogames DineOut
xtreg logLeptin_Se_P3 bed_hour sex_n AgeP3 nap_bi_n
i.medical_history_n vediogames DineOut
xtreg logLeptin_Se_P3 bed_weekend sex_n AgeP3 nap_bi_n
i.medical_history_n vediogames DineOut

xtreg logLeptin_Se_P3 bed12 sex_n AgeP3 nap_bi_n i.medical_history_n
vediogames DineOut
xtreg logLeptin_Se_P3 bed12bin_n sex_n AgeP3 nap_bi_n
i.medical_history_n vediogames DineOut
xtreg logLeptin_Se_P3 bed12_weekend_bi sex_n AgeP3 nap_bi_n
i.medical_history_n vediogames DineOut

xtreg logLeptin_Se_P3 i.bedtime123 sex_n AgeP3 nap_bi_n
i.medical_history_n vediogames DineOut
xtreg logLeptin_Se_P3 i.bedtimeWDcat sex_n AgeP3 nap_bi_n
i.medical_history_n vediogames DineOut
xtreg logLeptin_Se_P3 i.bedtimeWEcat sex_n AgeP3 nap_bi_n
i.medical_history_n vediogames DineOut

xtreg logLeptin_Se_P3 i.timeandduration sex_n AgeP3 nap_bi_n
i.medical_history_n vediogames DineOut

xtreg logLeptin_Se_P3 sleepdept sex_n AgeP3 nap_bi_n
i.medical_history_n vediogames DineOut
xtreg logLeptin_Se_P3 i.sleepdeptcat sex_n AgeP3 nap_bi_n
i.medical_history_n vediogames DineOut
xtreg logLeptin_Se_P3 i.sleepdeptsevere sex_n AgeP3 nap_bi_n
i.medical_history_n vediogames DineOut

xtreg logLeptin_Se_P3 social_getlag sex_n AgeP3 nap_bi_n
i.medical_history_n vediogames DineOut
xtreg logLeptin_Se_P3 i.socialgetlag_bi sex_n AgeP3 nap_bi_n
i.medical_history_n vediogames DineOut

*RANDOM EFFECT MODEL
xtreg VEGF_P3 AverageSleepDuration sex_n AgeP3 nap_bi_n
i.medical_history_n vediogames DineOut
xtreg VEGF_P3 Sleep_time_weekday_n sex_n AgeP3 nap_bi_n
i.medical_history_n vediogames DineOut
```

```
xtreg VEGF_P3 Sleep_time_weekend_n sex_n AgeP3 nap_bi_n
i.medical_history_n vediogames DineOut

xtreg VEGF_P3 bedtime_both2 sex_n AgeP3 nap_bi_n i.medical_history_n
vediogames DineOut
xtreg VEGF_P3 bed_hour sex_n AgeP3 nap_bi_n i.medical_history_n
vediogames DineOut
xtreg VEGF_P3 bed_weekend sex_n AgeP3 nap_bi_n i.medical_history_n
vediogames DineOut

xtreg VEGF_P3 bed12 sex_n AgeP3 nap_bi_n i.medical_history_n
vediogames DineOut
xtreg VEGF_P3 bed12bin_n sex_n AgeP3 nap_bi_n i.medical_history_n
vediogames DineOut
xtreg VEGF_P3 bed12_weekend_bi sex_n AgeP3 nap_bi_n
i.medical_history_n vediogames DineOut

xtreg VEGF_P3 i.bedtime123 sex_n AgeP3 nap_bi_n i.medical_history_n
vediogames DineOut
xtreg VEGF_P3 i.bedtimeWDcat sex_n AgeP3 nap_bi_n i.medical_history_n
vediogames DineOut
xtreg VEGF_P3 i.bedtimeWEcat sex_n AgeP3 nap_bi_n i.medical_history_n
vediogames DineOut

xtreg VEGF_P3 i.timeandduration sex_n AgeP3 nap_bi_n
i.medical_history_n vediogames DineOut

xtreg VEGF_P3 sleepdept sex_n AgeP3 nap_bi_n i.medical_history_n
vediogames DineOut
xtreg VEGF_P3 i.sleepdeptcat sex_n AgeP3 nap_bi_n i.medical_history_n
vediogames DineOut
xtreg VEGF_P3 i.sleepdeptsevere sex_n AgeP3 nap_bi_n
i.medical_history_n vediogames DineOut

xtreg VEGF_P3 social_getlag sex_n AgeP3 nap_bi_n i.medical_history_n
vediogames DineOut
xtreg VEGF_P3 i.socialgetlag_bi sex_n AgeP3 nap_bi_n
i.medical_history_n vediogames DineOut

*RANDOM EFFECT MODEL
xtreg logVEGF_Se_P3 AverageSleepDuration sex_n AgeP3 nap_bi_n
i.medical_history_n vediogames DineOut
xtreg logVEGF_Se_P3 Sleep_time_weekday_n sex_n AgeP3 nap_bi_n
i.medical_history_n vediogames DineOut
xtreg logVEGF_Se_P3 Sleep_time_weekend_n sex_n AgeP3 nap_bi_n
i.medical_history_n vediogames DineOut

xtreg logVEGF_Se_P3 bedtime_both2 sex_n AgeP3 nap_bi_n
i.medical_history_n vediogames DineOut
xtreg logVEGF_Se_P3 bed_hour sex_n AgeP3 nap_bi_n i.medical_history_n
```

```
vediogames DineOut
xtreg logVEGF_Se_P3 bed_weekend sex_n AgeP3 nap_bi_n
i.medical_history_n vediogames DineOut

xtreg logVEGF_Se_P3 bed12 sex_n AgeP3 nap_bi_n i.medical_history_n
vediogames DineOut
xtreg logVEGF_Se_P3 bed12bin_n sex_n AgeP3 nap_bi_n
i.medical_history_n vediogames DineOut
xtreg logVEGF_Se_P3 bed12_weekend_bi sex_n AgeP3 nap_bi_n
i.medical_history_n vediogames DineOut

xtreg logVEGF_Se_P3 i.bedtime123 sex_n AgeP3 nap_bi_n
i.medical_history_n vediogames DineOut
xtreg logVEGF_Se_P3 i.bedtimeWDcat sex_n AgeP3 nap_bi_n
i.medical_history_n vediogames DineOut
xtreg logVEGF_Se_P3 i.bedtimeWEcat sex_n AgeP3 nap_bi_n
i.medical_history_n vediogames DineOut

xtreg logVEGF_Se_P3 i.timeandduration sex_n AgeP3 nap_bi_n
i.medical_history_n vediogames DineOut

xtreg logVEGF_Se_P3 sleepdept sex_n AgeP3 nap_bi_n i.medical_history_n
vediogames DineOut
xtreg logVEGF_Se_P3 i.sleepdeptcat sex_n AgeP3 nap_bi_n
i.medical_history_n vediogames DineOut
xtreg logVEGF_Se_P3 i.sleepdeptsevere sex_n AgeP3 nap_bi_n
i.medical_history_n vediogames DineOut

xtreg logVEGF_Se_P3 social_getlag sex_n AgeP3 nap_bi_n
i.medical_history_n vediogames DineOut
xtreg logVEGF_Se_P3 i.socialgetlag_bi sex_n AgeP3 nap_bi_n
i.medical_history_n vediogames DineOut

*RANDOM EFFECT MODEL
xtset
xtreg logIL10 AverageSleepDuration sex_n AgeP3 nap_bi_n
i.medical_history_n vediogames DineOut
xtreg logIL10 Sleep_time_weekday_n sex_n AgeP3 nap_bi_n
i.medical_history_n vediogames DineOut
xtreg logIL10 Sleep_time_weekend_n sex_n AgeP3 nap_bi_n
i.medical_history_n vediogames DineOut

xtreg logIL10 bedtime_both2 sex_n AgeP3 nap_bi_n i.medical_history_n
vediogames DineOut
xtreg logIL10 bed_hour sex_n AgeP3 nap_bi_n i.medical_history_n
vediogames DineOut
xtreg logIL10 bed_weekend sex_n AgeP3 nap_bi_n i.medical_history_n
vediogames DineOut

xtreg logIL10 bed12 sex_n AgeP3 nap_bi_n i.medical_history_n
```

```
vediogames DineOut
xtreg logIL10 bed12bin_n sex_n AgeP3 nap_bi_n i.medical_history_n
vediogames DineOut
xtreg logIL10 bed12_weekend_bi sex_n AgeP3 nap_bi_n
i.medical_history_n vediogames DineOut

xtreg logIL10 i.bedtime123 sex_n AgeP3 nap_bi_n i.medical_history_n
vediogames DineOut
xtreg logIL10 i.bedtimeWDcat sex_n AgeP3 nap_bi_n i.medical_history_n
vediogames DineOut
xtreg logIL10 i.bedtimeWEcat sex_n AgeP3 nap_bi_n i.medical_history_n
vediogames DineOut

xtreg logIL10 i.timeandduration sex_n AgeP3 nap_bi_n
i.medical_history_n vediogames DineOut

xtreg logIL10 sleepdept sex_n AgeP3 nap_bi_n i.medical_history_n
vediogames DineOut
xtreg logIL10 i.sleepdeptcat sex_n AgeP3 nap_bi_n i.medical_history_n
vediogames DineOut
xtreg logIL10 i.sleepdeptsevere sex_n AgeP3 nap_bi_n
i.medical_history_n vediogames DineOut

xtreg logIL10 social_getlag sex_n AgeP3 nap_bi_n i.medical_history_n
vediogames DineOut
xtreg logIL10 i.socialgetlag_bi sex_n AgeP3 nap_bi_n
i.medical_history_n vediogames DineOut

*RANDOM EFFECT MODEL
xtreg logIL10_Se AverageSleepDuration sex_n AgeP3 nap_bi_n
i.medical_history_n vediogames DineOut
xtreg logIL10_Se Sleep_time_weekday_n sex_n AgeP3 nap_bi_n
i.medical_history_n vediogames DineOut
xtreg logIL10_Se Sleep_time_weekend_n sex_n AgeP3 nap_bi_n
i.medical_history_n vediogames DineOut

xtreg logIL10_Se bedtime_both2 sex_n AgeP3 nap_bi_n
i.medical_history_n vediogames DineOut
xtreg logIL10_Se bed_hour sex_n AgeP3 nap_bi_n i.medical_history_n
vediogames DineOut
xtreg logIL10_Se bed_weekend sex_n AgeP3 nap_bi_n i.medical_history_n
vediogames DineOut

xtreg logIL10_Se bed12 sex_n AgeP3 nap_bi_n i.medical_history_n
vediogames DineOut
xtreg logIL10_Se bed12bin_n sex_n AgeP3 nap_bi_n i.medical_history_n
vediogames DineOut
xtreg logIL10_Se bed12_weekend_bi sex_n AgeP3 nap_bi_n
i.medical_history_n vediogames DineOut
```

```
xtreg logIL10_Se i.bedtime123 sex_n AgeP3 nap_bi_n i.medical_history_n
vediogames DineOut
xtreg logIL10_Se i.bedtimeWDcat sex_n AgeP3 nap_bi_n
i.medical_history_n vediogames DineOut
xtreg logIL10_Se i.bedtimeWEcat sex_n AgeP3 nap_bi_n
i.medical_history_n vediogames DineOut

xtreg logIL10_Se i.timeandduration sex_n AgeP3 nap_bi_n
i.medical_history_n vediogames DineOut

xtreg logIL10_Se sleepdept sex_n AgeP3 nap_bi_n i.medical_history_n
vediogames DineOut
xtreg logIL10_Se i.sleepdeptcat sex_n AgeP3 nap_bi_n
i.medical_history_n vediogames DineOut
xtreg logIL10_Se i.sleepdeptsevere sex_n AgeP3 nap_bi_n
i.medical_history_n vediogames DineOut

xtreg logIL10_Se social_getlag sex_n AgeP3 nap_bi_n
i.medical_history_n vediogames DineOut
xtreg logIL10_Se i.socialgetlag_bi sex_n AgeP3 nap_bi_n
i.medical_history_n vediogames DineOut

*RANDOM EFFECT MODEL
xtreg logIL8_P3 AverageSleepDuration sex_n AgeP3 nap_bi_n
i.medical_history_n vediogames DineOut
xtreg logIL8_P3 Sleep_time_weekday_n sex_n AgeP3 nap_bi_n
i.medical_history_n vediogames DineOut
xtreg logIL8_P3 Sleep_time_weekend_n sex_n AgeP3 nap_bi_n
i.medical_history_n vediogames DineOut

xtreg logIL8_P3 bedtime_both2 sex_n AgeP3 nap_bi_n i.medical_history_n
vediogames DineOut
xtreg logIL8_P3 bed_hour sex_n AgeP3 nap_bi_n i.medical_history_n
vediogames DineOut
xtreg logIL8_P3 bed_weekend sex_n AgeP3 nap_bi_n i.medical_history_n
vediogames DineOut

xtreg logIL8_P3 bed12 sex_n AgeP3 nap_bi_n i.medical_history_n
vediogames DineOut
xtreg logIL8_P3 bed12bin_n sex_n AgeP3 nap_bi_n i.medical_history_n
vediogames DineOut
xtreg logIL8_P3 bed12_weekend_bi sex_n AgeP3 nap_bi_n
i.medical_history_n vediogames DineOut

xtreg logIL8_P3 i.bedtime123 sex_n AgeP3 nap_bi_n i.medical_history_n
vediogames DineOut
xtreg logIL8_P3 i.bedtimeWDcat sex_n AgeP3 nap_bi_n
i.medical_history_n vediogames DineOut
xtreg logIL8_P3 i.bedtimeWEcat sex_n AgeP3 nap_bi_n
i.medical_history_n vediogames DineOut
```

```
xtreg logIL8_P3 i.timeandduration sex_n AgeP3 nap_bi_n
i.medical_history_n vediogames DineOut

xtreg logIL8_P3 sleepdept sex_n AgeP3 nap_bi_n i.medical_history_n
vediogames DineOut
xtreg logIL8_P3 i.sleepdeptcat sex_n AgeP3 nap_bi_n
i.medical_history_n vediogames DineOut
xtreg logIL8_P3 i.sleepdeptsevere sex_n AgeP3 nap_bi_n
i.medical_history_n vediogames DineOut

xtreg logIL8_P3 social_getlag sex_n AgeP3 nap_bi_n i.medical_history_n
vediogames DineOut
xtreg logIL8_P3 i.socialgetlag_bi sex_n AgeP3 nap_bi_n
i.medical_history_n vediogames DineOut

*RANDOM EFFECT MODEL
xtreg logIL8_Se_P3 AverageSleepDuration sex_n AgeP3 nap_bi_n
i.medical_history_n vediogames DineOut
xtreg logIL8_Se_P3 Sleep_time_weekday_n sex_n AgeP3 nap_bi_n
i.medical_history_n vediogames DineOut
xtreg logIL8_Se_P3 Sleep_time_weekend_n sex_n AgeP3 nap_bi_n
i.medical_history_n vediogames DineOut

xtreg logIL8_Se_P3 bedtime_both2 sex_n AgeP3 nap_bi_n
i.medical_history_n vediogames DineOut
xtreg logIL8_Se_P3 bed_hour sex_n AgeP3 nap_bi_n i.medical_history_n
vediogames DineOut
xtreg logIL8_Se_P3 bed_weekend sex_n AgeP3 nap_bi_n
i.medical_history_n vediogames DineOut

xtreg logIL8_Se_P3 bed12 sex_n AgeP3 nap_bi_n i.medical_history_n
vediogames DineOut
xtreg logIL8_Se_P3 bed12bin_n sex_n AgeP3 nap_bi_n i.medical_history_n
vediogames DineOut
xtreg logIL8_Se_P3 bed12_weekend_bi sex_n AgeP3 nap_bi_n
i.medical_history_n vediogames DineOut

xtreg logIL8_Se_P3 i.bedtime123 sex_n AgeP3 nap_bi_n
i.medical_history_n vediogames DineOut
xtreg logIL8_Se_P3 i.bedtimeWDcat sex_n AgeP3 nap_bi_n
i.medical_history_n vediogames DineOut
xtreg logIL8_Se_P3 i.bedtimeWEcat sex_n AgeP3 nap_bi_n
i.medical_history_n vediogames DineOut

xtreg logIL8_Se_P3 i.timeandduration sex_n AgeP3 nap_bi_n
i.medical_history_n vediogames DineOut

xtreg logIL8_Se_P3 sleepdept sex_n AgeP3 nap_bi_n i.medical_history_n
vediogames DineOut
```

```
xtreg logIL8_Se_P3 i.sleepdeptcat sex_n AgeP3 nap_bi_n
i.medical_history_n vediogames DineOut
xtreg logIL8_Se_P3 i.sleepdeptsevere sex_n AgeP3 nap_bi_n
i.medical_history_n vediogames DineOut

xtreg logIL8_Se_P3 social_getlag sex_n AgeP3 nap_bi_n
i.medical_history_n vediogames DineOut
xtreg logIL8_Se_P3 i.socialgetlag_bi sex_n AgeP3 nap_bi_n
i.medical_history_n vediogames DineOut

*RANDOM EFFECT
xtset
xtreg logCRP_P3 AverageSleepDuration sex_n AgeP3 nap_bi_n
i.medical_history_n vediogames DineOut
xtreg logCRP_P3 Sleep_time_weekday_n sex_n AgeP3 nap_bi_n
i.medical_history_n vediogames DineOut
xtreg logCRP_P3 Sleep_time_weekend_n sex_n AgeP3 nap_bi_n
i.medical_history_n vediogames DineOut

xtreg logCRP_P3 bedtime_both2 sex_n AgeP3 nap_bi_n i.medical_history_n
vediogames DineOut
xtreg logCRP_P3 bed_hour sex_n AgeP3 nap_bi_n i.medical_history_n
vediogames DineOut
xtreg logCRP_P3 bed_weekend sex_n AgeP3 nap_bi_n i.medical_history_n
vediogames DineOut

xtreg logCRP_P3 bed12 sex_n AgeP3 nap_bi_n i.medical_history_n
vediogames DineOut
xtreg logCRP_P3 bed12bin_n sex_n AgeP3 nap_bi_n i.medical_history_n
vediogames DineOut
xtreg logCRP_P3 bed12_weekend_bi sex_n AgeP3 nap_bi_n
i.medical_history_n vediogames DineOut

xtreg logCRP_P3 i.bedtime123 sex_n AgeP3 nap_bi_n i.medical_history_n
vediogames DineOut
xtreg logCRP_P3 i.bedtimeWDcat sex_n AgeP3 nap_bi_n
i.medical_history_n vediogames DineOut
xtreg logCRP_P3 i.bedtimeWEcat sex_n AgeP3 nap_bi_n
i.medical_history_n vediogames DineOut

xtreg logCRP_P3 i.timeandduration sex_n AgeP3 nap_bi_n
i.medical_history_n vediogames DineOut
*Significant

xtreg logCRP_P3 sleepdept sex_n AgeP3 nap_bi_n i.medical_history_n
vediogames DineOut
xtreg logCRP_P3 i.sleepdeptcat sex_n AgeP3 nap_bi_n
i.medical_history_n vediogames DineOut
xtreg logCRP_P3 i.sleepdeptsevere sex_n AgeP3 nap_bi_n
i.medical_history_n vediogames DineOut
```

```
xtreg logCRP_P3 social_getlag sex_n AgeP3 nap_bi_n i.medical_history_n
vediogames DineOut
xtreg logCRP_P3 i.socialgetlag_bi sex_n AgeP3 nap_bi_n
i.medical_history_n vediogames DineOut

*RANDOM EFFECT MODEL
xtreg HSCRPugml AverageSleepDuration sex_n AgeP3 nap_bi_n
i.medical_history_n vediogames DineOut
xtreg HSCRPugml Sleep_time_weekday_n sex_n AgeP3 nap_bi_n
i.medical_history_n vediogames DineOut
xtreg HSCRPugml Sleep_time_weekend_n sex_n AgeP3 nap_bi_n
i.medical_history_n vediogames DineOut

xtreg HSCRPugml bedtime_both2 sex_n AgeP3 nap_bi_n i.medical_history_n
vediogames DineOut
xtreg HSCRPugml bed_hour sex_n AgeP3 nap_bi_n i.medical_history_n
vediogames DineOut
xtreg HSCRPugml bed_weekend sex_n AgeP3 nap_bi_n i.medical_history_n
vediogames DineOut

xtreg HSCRPugml bed12 sex_n AgeP3 nap_bi_n i.medical_history_n
vediogames DineOut
*sig
xtreg HSCRPugml bed12bin_n sex_n AgeP3 nap_bi_n i.medical_history_n
vediogames DineOut
*sig
xtreg HSCRPugml bed12_weekend_bi sex_n AgeP3 nap_bi_n
i.medical_history_n vediogames DineOut

xtreg HSCRPugml i.bedtime123 sex_n AgeP3 nap_bi_n i.medical_history_n
vediogames DineOut
xtreg HSCRPugml i.bedtimeWDcat sex_n AgeP3 nap_bi_n
i.medical_history_n vediogames DineOut
xtreg HSCRPugml i.bedtimeWEcat sex_n AgeP3 nap_bi_n
i.medical_history_n vediogames DineOut

xtreg HSCRPugml i.timeandduration sex_n AgeP3 nap_bi_n
i.medical_history_n vediogames DineOut
*sig

xtreg HSCRPugml sleepdept sex_n AgeP3 nap_bi_n i.medical_history_n
vediogames DineOut
xtreg HSCRPugml i.sleepdeptcat sex_n AgeP3 nap_bi_n
i.medical_history_n vediogames DineOut
xtreg HSCRPugml i.sleepdeptsevere sex_n AgeP3 nap_bi_n
i.medical_history_n vediogames DineOut

xtreg HSCRPugml social_getlag sex_n AgeP3 nap_bi_n i.medical_history_n
vediogames DineOut
```

```
xtreg HSCRPugml i.socialgetlag_bi sex_n AgeP3 nap_bi_n
i.medical_history_n vediogames DineOut

*RANDOM EFFECT MODEL
xtreg logIL6_P3 AverageSleepDuration sex_n AgeP3 nap_bi_n
i.medical_history_n vediogames DineOut
xtreg logIL6_P3 Sleep_time_weekday_n sex_n AgeP3 nap_bi_n
i.medical_history_n vediogames DineOut
*sig
xtreg logIL6_P3 Sleep_time_weekend_n sex_n AgeP3 nap_bi_n
i.medical_history_n vediogames DineOut

xtreg logIL6_P3 bedtime_both2 sex_n AgeP3 nap_bi_n i.medical_history_n
vediogames DineOut
xtreg logIL6_P3 bed_hour sex_n AgeP3 nap_bi_n i.medical_history_n
vediogames DineOut
xtreg logIL6_P3 bed_weekend sex_n AgeP3 nap_bi_n i.medical_history_n
vediogames DineOut
*Sig

xtreg logIL6_P3 bed12 sex_n AgeP3 nap_bi_n i.medical_history_n
vediogames DineOut
*sig
xtreg logIL6_P3 bed12bin_n sex_n AgeP3 nap_bi_n i.medical_history_n
vediogames DineOut
*sig
xtreg logIL6_P3 bed12_weekend_bi sex_n AgeP3 nap_bi_n
i.medical_history_n vediogames DineOut

xtreg logIL6_P3 i.bedtime123 sex_n AgeP3 nap_bi_n i.medical_history_n
vediogames DineOut
xtreg logIL6_P3 i.bedtimeWDcat sex_n AgeP3 nap_bi_n
i.medical_history_n vediogames DineOut
*sig
xtreg logIL6_P3 i.bedtimeWEcat sex_n AgeP3 nap_bi_n
i.medical_history_n vediogames DineOut

xtreg logIL6_P3 i.timeandduration sex_n AgeP3 nap_bi_n
i.medical_history_n vediogames DineOut

xtreg logIL6_P3 sleepdept sex_n AgeP3 nap_bi_n i.medical_history_n
vediogames DineOut
xtreg logIL6_P3 i.sleepdeptcat sex_n AgeP3 nap_bi_n
i.medical_history_n vediogames DineOut
xtreg logIL6_P3 i.sleepdeptsevere sex_n AgeP3 nap_bi_n
i.medical_history_n vediogames DineOut

xtreg logIL6_P3 social_getlag sex_n AgeP3 nap_bi_n i.medical_history_n
vediogames DineOut
xtreg logIL6_P3 i.socialgetlag_bi sex_n AgeP3 nap_bi_n
```

```
i.medical_history_n vediogames DineOut

*RANDOM EFFECT MODEL
xtreg IL6_Se_P3 AverageSleepDuration sex_n AgeP3 nap_bi_n
i.medical_history_n vediogames DineOut
xtreg IL6_Se_P3 Sleep_time_weekday_n sex_n AgeP3 nap_bi_n
i.medical_history_n vediogames DineOut
xtreg IL6_Se_P3 Sleep_time_weekend_n sex_n AgeP3 nap_bi_n
i.medical_history_n vediogames DineOut

xtreg IL6_Se_P3 bedtime_both2 sex_n AgeP3 nap_bi_n i.medical_history_n
vediogames DineOut
xtreg IL6_Se_P3 bed_hour sex_n AgeP3 nap_bi_n i.medical_history_n
vediogames DineOut
xtreg IL6_Se_P3 bed_weekend sex_n AgeP3 nap_bi_n i.medical_history_n
vediogames DineOut
*sig

xtreg IL6_Se_P3 bed12 sex_n AgeP3 nap_bi_n i.medical_history_n
vediogames DineOut
*sig
xtreg IL6_Se_P3 bed12bin_n sex_n AgeP3 nap_bi_n i.medical_history_n
vediogames DineOut
*sig
xtreg IL6_Se_P3 bed12_weekend_bi sex_n AgeP3 nap_bi_n
i.medical_history_n vediogames DineOut

xtreg IL6_Se_P3 i.bedtime123 sex_n AgeP3 nap_bi_n i.medical_history_n
vediogames DineOut
xtreg IL6_Se_P3 i.bedtimeWDcat sex_n AgeP3 nap_bi_n
i.medical_history_n vediogames DineOut
*sig
xtreg IL6_Se_P3 i.bedtimeWEcat sex_n AgeP3 nap_bi_n
i.medical_history_n vediogames DineOut

xtreg IL6_Se_P3 i.timeandduration sex_n AgeP3 nap_bi_n
i.medical_history_n vediogames DineOut
*sig

xtreg IL6_Se_P3 sleepdept sex_n AgeP3 nap_bi_n i.medical_history_n
vediogames DineOut
xtreg IL6_Se_P3 i.sleepdeptcat sex_n AgeP3 nap_bi_n
i.medical_history_n vediogames DineOut
xtreg IL6_Se_P3 i.sleepdeptsevere sex_n AgeP3 nap_bi_n
i.medical_history_n vediogames DineOut

xtreg IL6_Se_P3 social_getlag sex_n AgeP3 nap_bi_n i.medical_history_n
vediogames DineOut
xtreg IL6_Se_P3 i.socialgetlag_bi sex_n AgeP3 nap_bi_n
i.medical_history_n vediogames DineOut
```

```
*RANDOM EFFECT MODEL
xtreg logAdiponectin_P3 AverageSleepDuration sex_n AgeP3 nap_bi_n
i.medical_history_n vediogames DineOut
xtreg logAdiponectin_P3 Sleep_time_weekday_n sex_n AgeP3 nap_bi_n
i.medical_history_n vediogames DineOut
xtreg logAdiponectin_P3 Sleep_time_weekend_n sex_n AgeP3 nap_bi_n
i.medical_history_n vediogames DineOut

xtreg logAdiponectin_P3 bedtime_both2 sex_n AgeP3 nap_bi_n
i.medical_history_n vediogames DineOut
xtreg logAdiponectin_P3 bed_hour sex_n AgeP3 nap_bi_n
i.medical_history_n vediogames DineOut
xtreg logAdiponectin_P3 bed_weekend sex_n AgeP3 nap_bi_n
i.medical_history_n vediogames DineOut

xtreg logAdiponectin_P3 bed12 sex_n AgeP3 nap_bi_n i.medical_history_n
vediogames DineOut
xtreg logAdiponectin_P3 bed12bin_n sex_n AgeP3 nap_bi_n
i.medical_history_n vediogames DineOut
xtreg logAdiponectin_P3 bed12_weekend_bi sex_n AgeP3 nap_bi_n
i.medical_history_n vediogames DineOut

xtreg logAdiponectin_P3 i.bedtime123 sex_n AgeP3 nap_bi_n
i.medical_history_n vediogames DineOut
xtreg logAdiponectin_P3 i.bedtimeWDcat sex_n AgeP3 nap_bi_n
i.medical_history_n vediogames DineOut
xtreg logAdiponectin_P3 i.bedtimeWEcat sex_n AgeP3 nap_bi_n
i.medical_history_n vediogames DineOut

xtreg logAdiponectin_P3 i.timeandduration sex_n AgeP3 nap_bi_n
i.medical_history_n vediogames DineOut

xtreg logAdiponectin_P3 sleepdept sex_n AgeP3 nap_bi_n
i.medical_history_n vediogames DineOut
xtreg logAdiponectin_P3 i.sleepdeptcat sex_n AgeP3 nap_bi_n
i.medical_history_n vediogames DineOut
xtreg logAdiponectin_P3 i.sleepdeptsevere sex_n AgeP3 nap_bi_n
i.medical_history_n vediogames DineOut

xtreg logAdiponectin_P3 social_getlag sex_n AgeP3 nap_bi_n
i.medical_history_n vediogames DineOut
xtreg logAdiponectin_P3 i.socialgetlag_bi sex_n AgeP3 nap_bi_n
i.medical_history_n vediogames DineOut


*RANDOM EFFECT MODEL
xtreg logAdiponectin_Se_P3 AverageSleepDuration sex_n AgeP3 nap_bi_n
i.medical_history_n vediogames DineOut
xtreg logAdiponectin_Se_P3 Sleep_time_weekday_n sex_n AgeP3 nap_bi_n
```

```
i.medical_history_n vediogames DineOut
xtreg logAdiponectin_Se_P3 Sleep_time_weekend_n sex_n AgeP3 nap_bi_n
i.medical_history_n vediogames DineOut

xtreg logAdiponectin_Se_P3 bedtime_both2 sex_n AgeP3 nap_bi_n
i.medical_history_n vediogames DineOut
xtreg logAdiponectin_Se_P3 bed_hour sex_n AgeP3 nap_bi_n
i.medical_history_n vediogames DineOut
xtreg logAdiponectin_Se_P3 bed_weekend sex_n AgeP3 nap_bi_n
i.medical_history_n vediogames DineOut

xtreg logAdiponectin_Se_P3 bed12 sex_n AgeP3 nap_bi_n
i.medical_history_n vediogames DineOut
xtreg logAdiponectin_Se_P3 bed12bin_n sex_n AgeP3 nap_bi_n
i.medical_history_n vediogames DineOut
xtreg logAdiponectin_Se_P3 bed12_weekend_bi sex_n AgeP3 nap_bi_n
i.medical_history_n vediogames DineOut

xtreg logAdiponectin_Se_P3 i.bedtime123 sex_n AgeP3 nap_bi_n
i.medical_history_n vediogames DineOut
xtreg logAdiponectin_Se_P3 i.bedtimeWDcat sex_n AgeP3 nap_bi_n
i.medical_history_n vediogames DineOut
xtreg logAdiponectin_Se_P3 i.bedtimeWEcat sex_n AgeP3 nap_bi_n
i.medical_history_n vediogames DineOut

xtreg logAdiponectin_Se_P3 i.timeandduration sex_n AgeP3 nap_bi_n
i.medical_history_n vediogames DineOut

xtreg logAdiponectin_Se_P3 sleepdept sex_n AgeP3 nap_bi_n
i.medical_history_n vediogames DineOut
xtreg logAdiponectin_Se_P3 i.sleepdeptcat sex_n AgeP3 nap_bi_n
i.medical_history_n vediogames DineOut
xtreg logAdiponectin_Se_P3 i.sleepdeptsevere sex_n AgeP3 nap_bi_n
i.medical_history_n vediogames DineOut

xtreg logAdiponectin_Se_P3 social_getlag sex_n AgeP3 nap_bi_n
i.medical_history_n vediogames DineOut
xtreg logAdiponectin_Se_P3 i.socialgetlag_bi sex_n AgeP3 nap_bi_n
i.medical_history_n vediogames DineOut

*Regression Models:
*First: HSCRP
regress HSCRPugml AverageSleepDuration sex_n i.School  AgeP3 nap_bi_n
i.medical_history_n vediogames DineOut, robust
regress HSCRPugml AverageSleepDuration sex_n i.School AgeP3 nap_bi_n
i.medical_history_n vediogames DineOut if OBWHO3_n == 0, robust
regress HSCRPugml AverageSleepDuration sex_n i.School AgeP3 nap_bi_n
i.medical_history_n vediogames DineOut if OBWHO3_n == 1, robust

regress HSCRPugml Sleep_time_weekday sex_n i.School AgeP3 nap_bi_n
```

```
i.medical_history_n vediogames DineOut, robust
regress HSCRPugml Sleep_time_weekday sex_n i.School AgeP3 nap_bi_n
i.medical_history_n vediogames DineOut if OBWHO3_n == 0, robust
regress HSCRPugml Sleep_time_weekday sex_n i.School AgeP3 nap_bi_n
i.medical_history_n vediogames DineOut if OBWHO3_n == 1, robust

regress HSCRPugml bed12 sex_n i.School AgeP3 nap_bi_n
i.medical_history_n vediogames DineOut, robust
regress HSCRPugml bed12 sex_n i.School AgeP3 nap_bi_n
i.medical_history_n vediogames DineOut if OBWHO3_n == 0, robust
regress HSCRPugml bed12 sex_n i.School AgeP3 nap_bi_n
i.medical_history_n vediogames DineOut if OBWHO3_n == 1, robust

regress HSCRPugml bed12bin_n sex_n i.School AgeP3 nap_bi_n
i.medical_history_n vediogames DineOut, robust
regress HSCRPugml bed12bin_n sex_n i.School AgeP3 nap_bi_n
i.medical_history_n vediogames DineOut if OBWHO3_n == 0, robust
regress HSCRPugml bed12bin_n sex_n i.School AgeP3 nap_bi_n
i.medical_history_n vediogames DineOut if OBWHO3_n == 1, robust

regress HSCRPugml bedtime_both2 sex_n i.School AgeP3 nap_bi_n
i.medical_history_n vediogames DineOut, robust
regress HSCRPugml bed_hour sex_n i.School AgeP3 nap_bi_n
i.medical_history_n vediogames DineOut, robust
regress HSCRPugml bed_weekend_ sex_n i.School AgeP3 nap_bi_n
i.medical_history_n vediogames DineOut, robust

regress HSCRPugml sleepdept sex_n i.School AgeP3 nap_bi_n
i.medical_history_n vediogames DineOut, robust
regress HSCRPugml social_getlag sex_n i.School AgeP3 nap_bi_n
i.medical_history_n vediogames DineOut, robust
*Conclusion: when it comes to sleepdept and social jetlag==> both IL-6
salica and CRP act in the same way and IL-6 is opposite, (and was
stat. sig. with IL-6 saliva only)

*Second: IL6 Serum
regress IL6_Se_P3 AverageSleepDuration sex_n i.School AgeP3 nap_bi_n
i.medical_history_n vediogames DineOut, robust
regress IL6_Se_P3 AverageSleepDuration sex_n i.School AgeP3 nap_bi_n
i.medical_history_n vediogames DineOut if OBWHO3_n == 0, robust
regress IL6_Se_P3 AverageSleepDuration sex_n i.School AgeP3 nap_bi_n
i.medical_history_n vediogames DineOut if OBWHO3_n == 1, robust

regress IL6_Se_P3 Sleep_time_weekday sex_n i.School AgeP3 nap_bi_n
i.medical_history_n vediogames DineOut, robust
regress IL6_Se_P3 Sleep_time_weekday sex_n i.School AgeP3 nap_bi_n
i.medical_history_n vediogames DineOut if OBWHO3_n == 0, robust
regress IL6_Se_P3 Sleep_time_weekday sex_n i.School AgeP3 nap_bi_n
i.medical_history_n vediogames DineOut if OBWHO3_n == 1, robust
```

```
regress IL6_Se_P3 bed12 sex_n i.School AgeP3 nap_bi_n
i.medical_history_n vediogames DineOut, robust
regress IL6_Se_P3 bed12 sex_n i.School AgeP3 nap_bi_n
i.medical_history_n vediogames DineOut if OBWHO3_n == 0, robust
regress IL6_Se_P3 bed12 sex_n i.School AgeP3 nap_bi_n
i.medical_history_n vediogames DineOut if OBWHO3_n == 1, robust

regress IL6_Se_P3 bed12bin_n sex_n i.School AgeP3 nap_bi_n
i.medical_history_n vediogames DineOut, robust
regress IL6_Se_P3 bed12bin_n sex_n i.School AgeP3 nap_bi_n
i.medical_history_n vediogames DineOut if OBWHO3_n == 0, robust
regress IL6_Se_P3 bed12bin_n sex_n i.School AgeP3 nap_bi_n
i.medical_history_n vediogames DineOut if OBWHO3_n == 1, robust

regress IL6_Se_P3 bedtime_both2 sex_n i.School AgeP3 nap_bi_n
i.medical_history_n vediogames DineOut, robust
regress IL6_Se_P3 bed_hour sex_n i.School AgeP3 nap_bi_n
i.medical_history_n vediogames DineOut, robust
regress IL6_Se_P3 bed_weekend_ sex_n i.School AgeP3 nap_bi_n
i.medical_history_n vediogames DineOut, robust
*For bedtime as continuous variable==> children go to sleep late (late
bedtime)on weekends have SIGNIFICANTLY higher IL-6 level in SERUM.

regress IL6_Se_P3 sleepdept sex_n i.School AgeP3 nap_bi_n
i.medical_history_n vediogames DineOut, robust
regress IL6_Se_P3 social_getlag sex_n i.School AgeP3 nap_bi_n
i.medical_history_n vediogames DineOut, robust
*Conclusion: when it comes to sleepdept and social jetlag==> both IL-6
salica and CRP act in the same way and IL-6 is opposite, (and was
stat. sig. with IL-6 saliva only)

*Third: IL6 Saliva ==> log
regress logIL6_P3 AverageSleepDuration sex_n i.School AgeP3 nap_bi_n
i.medical_history_n vediogames DineOut, robust
regress logIL6_P3 AverageSleepDuration sex_n i.School AgeP3 nap_bi_n
i.medical_history_n vediogames DineOut if OBWHO3_n == 0, robust
regress logIL6_P3 AverageSleepDuration sex_n i.School AgeP3 nap_bi_n
i.medical_history_n vediogames DineOut if OBWHO3_n == 1, robust

regress logIL6_P3 Sleep_time_weekday sex_n i.School AgeP3 nap_bi_n
i.medical_history_n vediogames DineOut, robust
regress logIL6_P3 Sleep_time_weekday sex_n i.School AgeP3 nap_bi_n
i.medical_history_n vediogames DineOut if OBWHO3_n == 0, robust
regress logIL6_P3 Sleep_time_weekday sex_n i.School AgeP3 nap_bi_n
i.medical_history_n vediogames DineOut if OBWHO3_n == 1, robust

regress logIL6_P3 bed12 sex_n i.School AgeP3 nap_bi_n
i.medical_history_n vediogames DineOut, robust
regress logIL6_P3 bed12 sex_n i.School AgeP3 nap_bi_n
i.medical_history_n vediogames DineOut if OBWHO3_n == 0, robust
```

```
regress logIL6_P3 bed12 sex_n i.School AgeP3 nap_bi_n
i.medical_history_n vediogames DineOut if OBWHO3_n == 1, robust

regress logIL6_P3 bed12bin_n sex_n i.School AgeP3 nap_bi_n
i.medical_history_n vediogames DineOut, robust
regress logIL6_P3 bed12bin_n sex_n i.School AgeP3 nap_bi_n
i.medical_history_n vediogames DineOut if OBWHO3_n == 0, robust
regress logIL6_P3 bed12bin_n sex_n i.School AgeP3 nap_bi_n
i.medical_history_n vediogames DineOut if OBWHO3_n == 1, robust

regress logIL6_P3 bedtime_both2 sex_n i.School AgeP3 nap_bi_n
i.medical_history_n vediogames DineOut, robust
regress logIL6_P3 bed_hour sex_n i.School AgeP3 nap_bi_n
i.medical_history_n vediogames DineOut, robust
regress logIL6_P3 bed_weekend sex_n i.School AgeP3 nap_bi_n
i.medical_history_n vediogames DineOut, robust
*For bedtime as continuous variable==> children go to sleep late (late
bedtime)on weekends have SIGNIFICANTLY lower IL-6 level in SALIVA.
* I can see that IL-6 is serum behaves opposite to that in saliva.

regress logIL6_P3 sleepdept sex_n i.School AgeP3 nap_bi_n
i.medical_history_n vediogames DineOut, robust
regress logIL6_P3 social_getlag sex_n i.School AgeP3 nap_bi_n
i.medical_history_n vediogames DineOut, robust
*Conclusion: when it comes to sleepdept and social jetlag==> both IL-6
salica and CRP act in the same way and IL-6 is opposite, (and was
stat. sig. with IL-6 saliva only)

********************************************************************
*************************Paper 1 Adiponectin************************
********************************************************************
Abeer Add the variable snore_bi_n and compare between effect on serum
and saliva (affected ==> dryness)
then add electronics

*First: Adiponectin Saliva:
regress logAdiponectin_P3 bed12bin_n
regress logAdiponectin_P3 AverageSleepDuration
logistic Adiponectin_P3_75 bed12bin_n
logistic Adiponectin_P3_75 AverageSleepDuration

*For Dr.Hend
regress logAdiponectin_P3 bed12bin_n AgeP3 i.sex_n i.School DineOut
i.nap_n
regress logAdiponectin_P3 AverageSleepDuration AgeP3 i.sex_n i.School
DineOut i.nap_n
logistic Adiponectin_P3_75 bed12bin_n AgeP3 i.sex_n i.School DineOut
i.nap_n
logistic Adiponectin_P3_75 AverageSleepDuration AgeP3 i.sex_n i.School
DineOut i.nap_n
```

```
*For the paper:
regress logAdiponectin_P3 bed12bin_n i.nap_n electronic OBWHO3_n
regress logAdiponectin_P3 AverageSleepDuration
regress logAdiponectin_P3 snore_bi_n
*******************************************************************
logistic Adiponectin_P3_75 bed12bin_n i.sex_n School i.nap_n dinner
electronic snore_bi_n
logistic Adiponectin_P3_75 AverageSleepDuration i.sex_n i.nap_n dinner
electronic snore_bi_n
*******************************************************************

*Second: Adiponectin Serum:
regress logAdiponectin_Se_P3 bed12bin_n
regress logAdiponectin_Se_P3 AverageSleepDuration
logistic Adiponectin_Se_P3_n bed12bin_n
logistic Adiponectin_Se_P3_n AverageSleepDuration

*For Dr.Hend
regress logAdiponectin_Se_P3 bed12bin_n AgeP3 i.sex_n i.School DineOut
i.nap_n
regress logAdiponectin_Se_P3 AverageSleepDuration AgeP3 i.sex_n
i.School DineOut i.nap_n
logistic Adiponectin_Se_P3_n AverageSleepDuration i.sex_n i.School
DineOut i.nap_n

*For the paper:
regress logAdiponectin_Se_P3 bed12bin_n i.nap_n AgeP3
regress logAdiponectin_Se_P3 AverageSleepDuration i.nap_n AgeP3
logistic Adiponectin_Se_P3_n AverageSleepDuration i.sex_n School
DineOut
logistic Adiponectin_Se_P3_n AverageSleepDuration i.sex_n School
i.nap_bi_n dinner
**********************************************************************
logistic Adiponectin_Se_P3_n bed12bin_n AgeP3 i.sex_n School DineOut
i.nap_n electronic snore_bi_n
logistic Adiponectin_Se_P3_n bed12bin_n i.sex_n School i.nap_n DineOut
electronic snore_bi_n
logistic Adiponectin_Se_P3_n bed12bin_n i.sex_n School i.nap_n dinner
electronic snore_bi_n
**********************************************************************

*Third: Saliva vs Serum Adiponectin:
regress logAdiponectin_P3 Adiponectin_Se_P3
**********************************************************************
**************************Paper 2 IL_6 ********************************
**********************************************************************
*First: IL-6 Saliva:
*Bivariate Analysis:
regress IL6_P3 bed12bin_n
```

```
regress IL6_P3 AverageSleepDuration
regress IL6_Se_P3 bed12bin_n
regress IL6_Se_P3 AverageSleepDuration

*For Dr. Hend:
regress IL6_P3 AverageSleepDuration AgeP3 i.sex_n School DineOut
i.nap_n electronic
regress IL6_Se_P3 AverageSleepDuration AgeP3 i.sex_n School DineOut
i.nap_n electronic

*For the paper:
regress IL6_P3 AverageSleepDuration i.nap_n i.sex_n School dinner
electronic
regress IL6_Se_P3 AverageSleepDuration i.nap_n i.sex_n School dinner
electronic
**********************************************************************
regress IL6_P3 bed12bin_n AgeP3 i.sex_n School DineOut i.nap_n
electronic snore_bi_n
regress IL6_Se_P3 bed12bin_n AgeP3 i.sex_n School DineOut i.nap_n
electronic
regress IL6_P3 bed12bin_n i.sex_n School i.nap_n dinner electronic
snore_bi_n
regress IL6_Se_P3 bed12bin_n i.sex_n i.School i.nap_n dinner
electronic
**********************************************************************

**********************************************************************
***************************Paper 3 HSCRPugml *************************
**********************************************************************
regress HSCRPugml bed12bin_n
regress HSCRPugml AverageSleepDuration

*For Dr. Hend:
regress HSCRPugml AverageSleepDuration AgeP3 i.sex_n School DineOut
i.nap_n
******
******

*For the paper:
regress HSCRPugml AverageSleepDuration i.sex_n School i.nap_n DineOut
electronic
**********************************************************************
regress HSCRPugml bed12bin_n AgeP3 i.sex_n i.School DineOut i.nap_n
electronic
regress HSCRPugml bed12bin_n i.sex_n i.School i.nap_n DineOut
electronic
**********************************************************************

* Effect Modifier for Adiponectin:
gen intSLEP12OBE= bed12bin_n * OBWHO3_n
```

```
* Analyses for IL6 saliva and serum:
regress logIL6_P3 bed12bin_n
regress logIL6_Se_P3 bed12bin_n

*by sleeping at or after 12
regress logAdiponectin_P3 bed12bin_n
logistic Adiponectin_P3_75 bed12bin_n
regress logAdiponectin_Se_P3 bed12bin_n
logistic Adiponectin_Se_P3_n bed12bin_n
regress logIL6_P3 bed12bin_n
regress IL6_Se_P3 bed12bin_n
regress logHSCRPugml bed12bin_n

regress logAdiponectin_P3 bed12bin_n OBWHO3_n intSLEP12OBE
electronic_n dinner snore_n
logistic Adiponectin_P3_75 bed12bin_n OBWHO3_n intSLEP12OBE
electronic_n dinner snore_n
regress logAdiponectin_Se_P3 bed12bin_n OBWHO3_n intSLEP12OBE sex_n
electronic_n dinner snore_n physical_act
logistic Adiponectin_Se_P3_n bed12bin_n OBWHO3_n sex_n physical_act
snore_n dinner
regress logIL6_P3 bed12bin_n sex_n physical_act snore_n dinner
regress IL6_Se_P3 bed12bin_n
regress logHSCRPugml bed12bin_n OBWHO3_n intSLEP12OBE

regress logAdiponectin_P3 bed12bin_n dinner physical_act OBWHO3_n
logistic Adiponectin_P3_75 bed12bin_n sex_n medical_history OBWHO3_n
regress logAdiponectin_Se_P3 bed12bin_n dinner AgeP3 sex_n School
nap_bi_n
regress logIL6_P3 bed12bin_n dinner AgeP3 i.sex_n School i.nap_bi_n
regress IL6_Se_P3 bed12bin_n dinner AgeP3 sex_n School nap_bi_n
regress logHSCRPugml bed12bin_n School nap_bi_n AgeP3 sex_n dinner

regress logAdiponectin_P3 bed12bin_n dinner AgeP3 sex_n School
nap_bi_n
logistic Adiponectin_P3_75 bed12bin_n dinner AgeP3 sex_n School
nap_bi_n
regress logAdiponectin_Se_P3 bed12bin_n dinner AgeP3 sex_n School
nap_bi_n
regress logIL6_P3 bed12bin_n dinner AgeP3 i.sex_n School i.nap_bi_n
regress IL6_Se_P3 bed12bin_n dinner AgeP3 sex_n School nap_bi_n
regress logHSCRPugml bed12bin_n School nap_bi_n AgeP3 sex_n dinner

*more confounders
regress logAdiponectin_P3 bed12bin_n electronic_n dinner AgeP3 sex_n
School nap_bi_n physical_act OBWHO3_n
logit Adiponectin_P3_75 bed12bin_n electronic_n dinner AgeP3 sex_n
School nap_bi_n physical_act OBWHO3_n
regress logAdiponectin_Se_P3 bed12bin_n electronic_n dinner AgeP3
```

```
sex_n School nap_bi_n physical_act OBWHO3_n
regress logIL6_P3 bed12bin_n electronic_n dinner AgeP3 i.sex_n School
i.nap_bi_n physical_act OBWHO3_n
regress IL6_Se_P3 bed12bin_n electronic_n dinner AgeP3 sex_n School
nap_bi_n OBWHO3_n physical_act
regress logHSCRPugml bed12bin_n School nap_bi_n AgeP3 sex_n dinner
electronic_n OBWHO3_n physical_act

*By sleep time weekend
regress logAdiponectin_P3 i.Sleep_time_weekend_n electronic_n dinner
mom_edu_n snore_n
logistic Adiponectin_P3_75 i.Sleep_time_weekend_n electronic_n dinner
mom_edu_n snore_n
regress logAdiponectin_Se_P3 i.Sleep_time_weekend_n electronic_n
dinner mom_edu_n snore_n
regress logIL6_P3 i.Sleep_time_weekend_n
regress IL6_Se_P3 i.Sleep_time_weekend_n
regress logHSCRPugml i.Sleep_time_weekend_n
*By sleep time weekday
regress logAdiponectin_P3 i.Sleep_time_weekday_n OBWHO3_n intSLEP12OBE
electronic_n dinner mom_edu_n snore_n
logistic Adiponectin_P3_75 i.Sleep_time_weekday_n OBWHO3_n
intSLEP12OBE electronic_n dinner mom_edu_n snore_n
regress logAdiponectin_Se_P3 i.Sleep_time_weekday_n OBWHO3_n
intSLEP12OBE electronic_n dinner mom_edu_n snore_n
regress logIL6_P3 i.Sleep_time_weekday_n
regress IL6_Se_P3 i.Sleep_time_weekday_n
regress logHSCRPugml i.Sleep_time_weekday_n

**************By biomarker*****************
*First: Salivary adiponectin
regress logAdiponectin_P3 sleep_duration6_n electronic_n dinner AgeP3
sex_n School nap_bi_n physical_act OBWHO3_n
regress logAdiponectin_P3 bed12bin_n School nap_bi_n AgeP3 sex_n
dinner
regress logAdiponectin_P3 bed12bin_n School nap_bi_n AgeP3 sex_n
dinner electronic_n
regress logAdiponectin_P3 AverageSleepDuration School nap_bi_n AgeP3
sex_n dinner
regress logAdiponectin_P3 sleep_duration6_n electronic_n dinner AgeP3
sex_n School nap_bi_n physical_act OBWHO3_n

*Second: Serum Adiponectin
regress logAdiponectin_Se_P3 bed12bin_n School nap_bi_n AgeP3 sex_n
DineOut
regress logAdiponectin_Se_P3 bed12bin_n School nap_bi_n AgeP3 sex_n
dinner
regress logAdiponectin_Se_P3 bed12bin_n School nap_bi_n AgeP3 sex_n
DineOut electronic_n
regress logAdiponectin_Se_P3 AverageSleepDuration School nap_bi_n
```

```
AgeP3 sex_n DineOut
regress logAdiponectin_Se_P3 AverageSleepDuration School i.nap_bi_n
AgeP3 i.sex_n i.dinner
regress logAdiponectin_P3 sleep_duration6_n electronic_n dinner AgeP3
sex_n School nap_bi_n physical_act OBWHO3_n

*Third: Salivary IL6
stepwise, pr(.10): regress IL6_P3 School sex_n AgeP3 med_hist_n
medical_history_n meds_n mom_diab_n dad_diab_n mom_edu_n mother_ed_n
dad_edu_n fath_ed_n sleep_weekday_n wakeup_weekday_n
Sleep_time_weekday_n sleep_weekend_n wakeup_weekend_n
Sleep_time_weekend_n interupted_sleep_n interupted_sleep_bi_n
wakeup_night_n wakeupnight_bin_n sleepy_day_n sleepy_day_bi_n
hard_wakeup_n hard_wakeup_bi_n diff_fall_asleep_n
diff_fall_asleep_bi_n quality_sleep_n snore_n snore_bi_n nap_n
nap_bi_n sleep_apnea_max_n bed12bin_n bed10_bin_n sleep_duration6_n
sleep_duration7_n sleep_duration8_n WHOcat3_n OBWHO3_n electronic_n
dinner physical_act
regress logIL6_P3 sleep_duration6_n electronic_n dinner AgeP3 sex_n
School nap_bi_n physical_act OBWHO3_n
regress logIL6_P3 i.bed12bin_n electronic_n dinner AgeP3 i.sex_n
School i.nap_bi_n physical_act OBWHO3_n
regress logIL6_P3 AverageSleepDuration School i.nap_bi_n AgeP3 i.sex_n
i.dinner
regress logIL6_P3 AverageSleepDuration School i.nap_bi_n AgeP3 i.sex_n
DineOut

*Fourth: Serum IL6
stepwise, pr(.10): regress IL6_Se_P3 School sex_n AgeP3 med_hist_n
medical_history_n meds_n mom_diab_n dad_diab_n mom_edu_n mother_ed_n
dad_edu_n fath_ed_n sleep_weekday_n wakeup_weekday_n
Sleep_time_weekday_n sleep_weekend_n wakeup_weekend_n
Sleep_time_weekend_n interupted_sleep_n interupted_sleep_bi_n
wakeup_night_n wakeupnight_bin_n sleepy_day_n sleepy_day_bi_n
hard_wakeup_n hard_wakeup_bi_n diff_fall_asleep_n
diff_fall_asleep_bi_n quality_sleep_n snore_n snore_bi_n nap_n
nap_bi_n sleep_apnea_max_n bed12bin_n bed10_bin_n sleep_duration6_n
sleep_duration7_n sleep_duration8_n WHOcat3_n OBWHO3_n electronic_n
dinner physical_act
regress IL6_Se_P3 bed12bin_n School nap_bi_n AgeP3 sex_n DineOut
regress IL6_Se_P3 bed12bin_n School nap_bi_n AgeP3 sex_n dinner
regress IL6_Se_P3 bed12bin_n School nap_bi_n AgeP3 sex_n DineOut
electronic_n
regress IL6_Se_P3 AverageSleepDuration School nap_bi_n AgeP3 sex_n
DineOut
regress IL6_Se_P3 AverageSleepDuration School nap_bi_n AgeP3 sex_n
DineOut electronic_n
regress IL6_Se_P3 sleep_duration6_n electronic_n dinner AgeP3 sex_n
School nap_bi_n physical_act OBWHO3_n
```

```
*Fifth: HSCRPugml
stepwise, pr(.10): regress logHSCRPugml School sex_n AgeP3 med_hist_n
medical_history_n meds_n mom_diab_n dad_diab_n mom_edu_n mother_ed_n
dad_edu_n fath_ed_n sleep_weekday_n wakeup_weekday_n
Sleep_time_weekday_n sleep_weekend_n wakeup_weekend_n
Sleep_time_weekend_n interupted_sleep_n interupted_sleep_bi_n
wakeup_night_n wakeupnight_bin_n sleepy_day_n sleepy_day_bi_n
hard_wakeup_n hard_wakeup_bi_n diff_fall_asleep_n
diff_fall_asleep_bi_n quality_sleep_n snore_n snore_bi_n nap_n
nap_bi_n sleep_apnea_max_n bed12bin_n bed10_bin_n sleep_duration6_n
sleep_duration7_n sleep_duration8_n WHOcat3_n OBWHO3_n electronic_n
dinner physical_act
regress logHSCRPugml bed12bin_n School nap_bi_n AgeP3 sex_n DineOut
regress logHSCRPugml bed12bin_n School nap_bi_n AgeP3 sex_n dinner
regress logHSCRPugml bed12bin_n School nap_bi_n AgeP3 sex_n dinner
electronic_n
regress logHSCRPugml AverageSleepDuration nap_bi_n AgeP3 sex_n dinner
regress logHSCRPugml sleep_duration6_n electronic_n dinner AgeP3 sex_n
School nap_bi_n physical_act OBWHO3_n


***Regression almost same above but divided by Sleep variables:
*First: sleep duration 6 hours:
regress logAdiponectin_P3 sleep_duration6_n electronic_n dinner AgeP3
sex_n School nap_bi_n physical_act OBWHO3_n
regress logAdiponectin_Se_P3 sleep_duration6_n electronic_n dinner
AgeP3 sex_n School nap_bi_n physical_act OBWHO3_n
regress logIL6_P3 sleep_duration6_n electronic_n dinner AgeP3 sex_n
School nap_bi_n physical_act OBWHO3_n
regress IL6_Se_P3 sleep_duration6_n electronic_n dinner AgeP3 sex_n
School nap_bi_n physical_act OBWHO3_n
regress logHSCRPugml sleep_duration6_n electronic_n dinner AgeP3 sex_n
School nap_bi_n physical_act OBWHO3_n

*Second: bed12bin_n
regress logAdiponectin_P3 bed12bin_n electronic_n dinner AgeP3 sex_n
School nap_bi_n physical_act OBWHO3_n
logit Adiponectin_P3_75 bed12bin_n electronic_n dinner AgeP3 sex_n
School nap_bi_n physical_act OBWHO3_n
regress logAdiponectin_Se_P3 bed12bin_n electronic_n dinner AgeP3
sex_n School nap_bi_n physical_act OBWHO3_n
regress logIL6_P3 bed12bin_n electronic_n dinner AgeP3 i.sex_n School
i.nap_bi_n physical_act OBWHO3_n
regress IL6_Se_P3 bed12bin_n electronic_n dinner AgeP3 sex_n School
nap_bi_n OBWHO3_n physical_act
regress logHSCRPugml bed12bin_n School nap_bi_n AgeP3 sex_n dinner
electronic_n OBWHO3_n physical_act

regress logAdiponectin_P3 bed12bin_n electronic_n dinner sex_n School
nap_bi_n physical_act OBWHO3_n wakeup_night_n
```

```
logit Adiponectin_P3_75 bed12bin_n electronic_n dinner sex_n School
nap_bi_n physical_act OBWHO3_n wakeup_night_n
regress logAdiponectin_Se_P3 bed12bin_n electronic_n dinner sex_n
School nap_bi_n physical_act OBWHO3_n interupted_sleep_n
regress logIL6_P3 bed12bin_n electronic_n dinner sex_n School
i.nap_bi_n physical_act OBWHO3_n interupted_sleep_n
regress IL6_Se_P3 bed12bin_n electronic_n dinner sex_n School nap_bi_n
OBWHO3_n physical_act interupted_sleep_n wakeup_night_n
regress logHSCRPugml bed12bin_n electronic_n dinner sex_n School
nap_bi_n OBWHO3_n physical_act interupted_sleep_n wakeup_night_n


*Third: AverageSleepDuration
regress logAdiponectin_P3 AverageSleepDuration School nap_bi_n AgeP3
sex_n dinner OBWHO3_n physical_act
regress logAdiponectin_Se_P3 AverageSleepDuration School nap_bi_n
AgeP3 sex_n dinner OBWHO3_n physical_act
regress logIL6_P3 AverageSleepDuration School nap_bi_n AgeP3 sex_n
dinner OBWHO3_n physical_act
regress IL6_Se_P3 AverageSleepDuration School nap_bi_n AgeP3 sex_n
dinner electronic_n OBWHO3_n physical_act
regress logHSCRPugml AverageSleepDuration School nap_bi_n AgeP3 sex_n
dinner OBWHO3_n physical_act electronic


regress logAdiponectin_P3 i.AverageSleepDuration_cat School nap_bi_n
AgeP3 sex_n dinner OBWHO3_n physical_act
regress logAdiponectin_Se_P3 i.AverageSleepDuration_cat School
nap_bi_n AgeP3 sex_n dinner OBWHO3_n physical_act
regress logIL6_P3 i.AverageSleepDuration_cat School nap_bi_n AgeP3
sex_n dinner OBWHO3_n physical_act
regress IL6_Se_P3 i.AverageSleepDuration_cat School nap_bi_n AgeP3
sex_n dinner electronic_n OBWHO3_n physical_act
regress logHSCRPugml i.AverageSleepDuration_cat School nap_bi_n AgeP3
sex_n dinner OBWHO3_n physical_act electronic


*Fourth:
regress logAdiponectin_P3 AverageSleepDuration_bi School nap_bi_n
AgeP3 sex_n dinner OBWHO3_n physical_act
regress logAdiponectin_Se_P3 AverageSleepDuration_bi School nap_bi_n
AgeP3 sex_n dinner OBWHO3_n physical_act
regress logIL6_P3 AverageSleepDuration_bi School nap_bi_n AgeP3 sex_n
dinner OBWHO3_n physical_act
regress IL6_Se_P3 AverageSleepDuration_bi School nap_bi_n AgeP3 sex_n
dinner electronic_n OBWHO3_n physical_act
regress logHSCRPugml AverageSleepDuration_bi School nap_bi_n AgeP3
sex_n dinner OBWHO3_n physical_act electronic

*Fifth: Average sleeptime in a week:
```

```
regress logAdiponectin_P3 Ave_Sleep_time_week School nap_bi_n AgeP3
sex_n dinner OBWHO3_n physical_act
regress logAdiponectin_Se_P3 Ave_Sleep_time_week School nap_bi_n AgeP3
sex_n dinner OBWHO3_n physical_act
regress logIL6_P3 Ave_Sleep_time_week School nap_bi_n AgeP3 sex_n
dinner OBWHO3_n physical_act
regress IL6_Se_P3 Ave_Sleep_time_week School nap_bi_n AgeP3 sex_n
dinner electronic_n OBWHO3_n physical_act
regress logHSCRPugml Ave_Sleep_time_week School nap_bi_n AgeP3 sex_n
dinner OBWHO3_n physical_act electronic

*Sixth:Sleep_time_weekday_n:
regress logAdiponectin_P3 Sleep_time_weekday_n School nap_bi_n AgeP3
sex_n dinner OBWHO3_n physical_act electronic
regress logAdiponectin_Se_P3 Sleep_time_weekday_n School nap_bi_n
AgeP3 sex_n dinner OBWHO3_n physical_act electronic
regress logIL6_P3 Sleep_time_weekday_n School nap_bi_n AgeP3 sex_n
dinner OBWHO3_n physical_act electronic
regress IL6_Se_P3 Sleep_time_weekday_n School nap_bi_n AgeP3 sex_n
dinner OBWHO3_n physical_act electronic
regress logHSCRPugml Sleep_time_weekday_n School nap_bi_n AgeP3 sex_n
dinner OBWHO3_n physical_act electronic


==============================
******Regression almost same above but divided by Sleep variables but
with less confounding variables:
*First: sleep duration 6 hours:
regress logAdiponectin_P3 sleep_duration6_
regress logAdiponectin_Se_P3 sleep_duration6_n
regress logIL6_P3 sleep_duration6_n
regress IL6_Se_P3 sleep_duration6_n
regress logHSCRPugml sleep_duration6_n

regress logAdiponectin_P3 sleep_duration6_n dinner AgeP3 sex_n School
nap_bi_n
regress logAdiponectin_Se_P3 sleep_duration6_n dinner AgeP3 sex_n
School nap_bi
regress logIL6_P3 sleep_duration6_n  dinner AgeP3 sex_n School
nap_bi_n
regress IL6_Se_P3 sleep_duration6_n dinner AgeP3 sex_n School nap_bi_n
regress logHSCRPugml sleep_duration6_n dinner AgeP3 sex_n School
nap_bi_n

*Second: bed12bin_n
regress logAdiponectin_P3 bed12bin_n dinner AgeP3 sex_n School
nap_bi_n
regress logAdiponectin_P3_75 bed12bin_n dinner AgeP3 sex_n School
nap_bi_n
regress logAdiponectin_Se_P3 bed12bin_n dinner AgeP3 sex_n School
```

```
nap_bi_n
regress logIL6_P3 bed12bin_n dinner AgeP3 i.sex_n School i.nap_bi_n
regress IL6_Se_P3 bed12bin_n dinner AgeP3 sex_n School nap_bi_n
regress logHSCRPugml bed12bin_n School nap_bi_n AgeP3 sex_n dinner

*Third: AverageSleepDuration / day
regress logAdiponectin_P3 AverageSleepDuration School nap_bi_n AgeP3
sex_n dinner
regress logAdiponectin_Se_P3 AverageSleepDuration School nap_bi_n
AgeP3 sex_n dinner
regress logIL6_P3 AverageSleepDuration School nap_bi_n AgeP3 sex_n
dinner
regress IL6_Se_P3 AverageSleepDuration School nap_bi_n AgeP3 sex_n
dinner
regress logHSCRPugml AverageSleepDuration School nap_bi_n AgeP3 sex_n
dinner

*Fourth: AverageSleepDuration / day_binary
regress logAdiponectin_P3 AverageSleepDuration_bi School nap_bi_n
AgeP3 sex_n dinner
regress logAdiponectin_Se_P3 AverageSleepDuration_bi School nap_bi_n
AgeP3 sex_n dinner
regress logIL6_P3 AverageSleepDuration_bi School nap_bi_n AgeP3 sex_n
dinner
regress IL6_Se_P3 AverageSleepDuration_bi School nap_bi_n AgeP3 sex_n
dinner
regress logHSCRPugml AverageSleepDuration_bi School nap_bi_n AgeP3
sex_n dinner

*Fifth: Average Sleep time a week
regress logAdiponectin_P3 Ave_Sleep_time_week School nap_bi_n AgeP3
sex_n dinner
regress logAdiponectin_Se_P3 Ave_Sleep_time_week School nap_bi_n AgeP3
sex_n dinner
regress logIL6_P3 Ave_Sleep_time_week School nap_bi_n AgeP3 sex_n
dinner
regress IL6_Se_P3 Ave_Sleep_time_week School nap_bi_n AgeP3 sex_n
dinner
regress logHSCRPugml Ave_Sleep_time_week School nap_bi_n AgeP3 sex_n
dinner

*Sixth:Sleep_time_weekday_n:
regress logAdiponectin_P3 Sleep_time_weekday_n School nap_bi_n AgeP3
sex_n dinner
regress logAdiponectin_Se_P3 Sleep_time_weekday_n School nap_bi_n
AgeP3 sex_n dinner
regress logIL6_P3 Sleep_time_weekday_n School nap_bi_n AgeP3 sex_n
dinner
regress IL6_Se_P3 Sleep_time_weekday_n School nap_bi_n AgeP3 sex_n
dinner
```

```
regress logHSCRPugml Sleep_time_weekday_n School nap_bi_n AgeP3 sex_n
dinner
```

```
*********Multivariate linear and logistic regression
analysi**************
***First: Salivary adiponectin
stepwise, pr(.10): regress logAdiponectin_P3 logUCN3_Se_P3
logAdiponectin_Se_P3 School sex_n AgeP3 medical_history_n meds_n
mom_diab_n dad_diab_n mom_edu_n mother_ed_n dad_edu_n fath_ed_n
sleep_weekday_n wakeup_weekday_n Sleep_time_weekday_n sleep_weekend_n
wakeup_weekend_n Sleep_time_weekend_n interupted_sleep_n
interupted_sleep_bi_n wakeup_night_n wakeupnight_bin_n sleepy_day_n
sleepy_day_bi_n hard_wakeup_n hard_wakeup_bi_n diff_fall_asleep_n
diff_fall_asleep_bi_n quality_sleep_n snore_n snore_bi_n nap_n
nap_bi_n sleep_apnea_max_n bed12bin_n bed10_bin_n sleep_duration6_n
sleep_duration7_n sleep_duration8_n WHOcat3_n OBWHO3_n electronic_n
dinner physical_act

regress logAdiponectin_P3 Sleep_time_weekday_n
regress logAdiponectin_P3 Sleep_time_weekday_n fath_ed_n mother_ed_n
medical_history_n OBWHO3_n sex_n electronic_n dinner
regress logAdiponectin_P3 Sleep_time_weekday_n i.fath_ed_n
i.mother_ed_n i.medical_history_n i.OBWHO3_n i.sex_n i.electronic_n
dinner

tab Adiponectin_P3_n
stepwise, pr(0.10): logit Adiponectin_P3_n logUCN3_Se_P3
logAdiponectin_Se_P3 School sex_n AgeP3 med_hist_n medical_history_n
meds_n mom_diab_n dad_diab_n mom_edu_n mother_ed_n dad_edu_n fath_ed_n
sleep_weekday_n wakeup_weekday_n Sleep_time_weekday_n sleep_weekend_n
wakeup_weekend_n Sleep_time_weekend_n interupted_sleep_n
interupted_sleep_bi_n wakeup_night_n wakeupnight_bin_n sleepy_day_n
sleepy_day_bi_n hard_wakeup_n hard_wakeup_bi_n diff_fall_asleep_n
diff_fall_asleep_bi_n quality_sleep_n snore_n snore_bi_n nap_n
nap_bi_n sleep_apnea_max_n bed12bin_n bed10_bin_n sleep_duration6_n
sleep_duration7_n sleep_duration8_n WHOcat3_n OBWHO3_n electronic_n
dinner physical_act

logit Adiponectin_P3_n Sleep_time_weekday_n, or
logit Adiponectin_P3_n Sleep_time_weekday_n mother_ed_n mom_diab_n
medical_history_n OBWHO3_n electronic_n dinner physical_act, or
logit Adiponectin_P3_n Sleep_time_weekday_n i.mother_ed_n i.mom_diab_n
i.medical_history_n i.OBWHO3_n i.electronic_n i.dinner i.physical_act,
or

regress logAdiponectin_P3 bed12bin_n
```

```
regress logAdiponectin_P3 bed12bin_n sex_n School medical_history_n
mother_ed_n mom_diab_n OBWHO3_n electronic_n dinner physical_act nap_n
regress logAdiponectin_P3 bed12bin_n i.sex_n School
i.medical_history_n i.mother_ed_n i.mom_diab_n i.OBWHO3_n
i.electronic_n i.dinner i.physical_act i.nap_n

tab Adiponectin_P3_n
logit Adiponectin_P3_n bed12bin_n mother_ed_n, or
logit Adiponectin_P3_n bed12bin_n mother_ed_n mom_diab_n
medical_history_n sex_n School OBWHO3_n electronic_n dinner
physical_act snore_n osa, or

regress logAdiponectin_P3 sleep_duration6_n
regress logAdiponectin_P3 sleep_duration6_n mom_diab_n fath_ed_n
mother_ed_n OBWHO3_n electronic_n dinner physical_act

logit Adiponectin_P3_n sleep_duration6_n, or
logit Adiponectin_P3_n sleep_duration6_n mother_ed_n mom_diab_n
electronic_n physical_act dinner snore_n, or


*Second: Serum adiponectin
stepwise, pr(.10): regress logAdiponectin_Se_P3 logUCN3_Se_P3
logAdiponectin_P3 School sex_n AgeP3 med_hist_n medical_history_n
meds_n mom_diab_n dad_diab_n mom_edu_n mother_ed_n dad_edu_n fath_ed_n
sleep_weekday_n wakeup_weekday_n Sleep_time_weekday_n sleep_weekend_n
wakeup_weekend_n Sleep_time_weekend_n interupted_sleep_n
interupted_sleep_bi_n wakeup_night_n wakeupnight_bin_n sleepy_day_n
sleepy_day_bi_n hard_wakeup_n hard_wakeup_bi_n diff_fall_asleep_n
diff_fall_asleep_bi_n quality_sleep_n snore_n snore_bi_n nap_n
nap_bi_n sleep_apnea_max_n bed12bin_n bed10_bin_n sleep_duration6_n
sleep_duration7_n sleep_duration8_n WHOcat3_n OBWHO3_n electronic_n
dinner physical_act

regress logAdiponectin_Se_P3 Sleep_time_weekday_n
regress logAdiponectin_Se_P3 Sleep_time_weekday_n mother_ed_n sex_n
dinner physical_act snore_n nap_n

logit Adiponectin_Se_P3_n Sleep_time_weekday_n, or
logit Adiponectin_Se_P3_n Sleep_time_weekday_n sex_n physical_act
dinner School mother_ed_n fath_ed_n dad_diab_n snore_n, or

regress logAdiponectin_Se_P3 bed12bin_n
regress logAdiponectin_Se_P3 bed12bin_n sex_n AgeP3 mother_ed_n
fath_ed_n physical_act osa

logit Adiponectin_Se_P3_n bed12bin_n, or
logit Adiponectin_Se_P3_n bed12bin_n sex_n mother_ed_n physical_act,
or
```

```
regress logAdiponectin_Se_P3 sleep_duration6_n
regress logAdiponectin_Se_P3 sleep_duration6_n sex_n mother_ed_n
physical_act dinner snore_n

logit Adiponectin_Se_P3_n sleep_duration6_n, or
logit Adiponectin_Se_P3_n sleep_duration6_n sex_n mother_ed_n
dad_diab_n physical_act dinner, or



*Fifth: Serum and salivary adiponectin
regress logAdiponectin_Se_P3 logAdiponectin_P3
regress logAdiponectin_Se_P3 logAdiponectin_P3 sex_n dad_diab_n
physical_act OBWHO3_n nap_n

*Some significant associatins of Adiponectin (serum and saliva), gene
UCN3 (serum)with sleep variables:
logit Adiponectin_P3_n snore_n
logit Adiponectin_P3_n snore_bi_n
logit Adiponectin_P3_n wakeup_night_n
logit Adiponectin_P3_n sleep_duration6_n

logit Adiponectin_Se_P3_n wakeup_weekend_n
logit Adiponectin_Se_P3_n interupted_sleep_n
logit Adiponectin_Se_P3_n interupted_sleep_n interupted_sleep_bi_n
logit Adiponectin_Se_P3_n interupted_sleep_n interupted_sleep_bi_n
wakeup_night_n
logit Adiponectin_Se_P3_n sleepy_day_n
logit Adiponectin_Se_P3_n sleepy_day_bi_n
logit Adiponectin_Se_P3_n snore_n
logit Adiponectin_Se_P3_n snore_n nap_n
logit Adiponectin_Se_P3_n nap_bi_n
logit Adiponectin_Se_P3_n sleep_apnea_max_n
logit Adiponectin_Se_P3_n bed12bin_n
logit Adiponectin_Se_P3_n sleep_duration6_n

logit UCN3_Se_P3_n diff_fall_asleep_bi_n


*Significant Associations:
regress logAdiponectin_P3 Sleep_time_weekday_n i.fath_ed_n
i.mother_ed_n i.medical_history_n i.OBWHO3_n i.sex_n i.electronic_n
dinner
logit Adiponectin_P3_n Sleep_time_weekday_n i.mother_ed_n i.mom_diab_n
i.medical_history_n i.OBWHO3_n i.electronic_n i.dinner i.physical_act,
or
regress logAdiponectin_P3 bed12bin_n i.sex_n School
i.medical_history_n i.mother_ed_n i.mom_diab_n i.OBWHO3_n
i.electronic_n i.dinner i.physical_act i.nap_n
regress logAdiponectin_P3 sleep_duration6_n mom_diab_n fath_ed_n
```

```
mother_ed_n OBWHO3_n electronic_n dinner physical_act
logit Adiponectin_P3_n sleep_duration6_n mother_ed_n mom_diab_n
electronic_n physical_act dinner snore_n, or

logit Adiponectin_Se_P3_n bed12bin_n sex_n mother_ed_n physical_act,
or
logit Adiponectin_Se_P3_n sleep_duration6_n sex_n mother_ed_n
dad_diab_n physical_act dinner, or

regress logAdiponectin_Se_P3 logAdiponectin_P3 sex_n dad_diab_n
physical_act OBWHO3_n nap_n


***************Analyses not to be used anymore *******************
***** Correlation Analysis==> strength of the linear relationship bet
2 varaibles==> does not imply causation
* and Abeer check for colinearity to avoid it in your regression.
*First: Adiponectin in saliva:
scatter Adiponectin_P3 Sleep_time_weekday
pwcorr Adiponectin_P3 Sleep_time_weekday, sig obs
*Lowess Smoothing to assess for linearity==> to check if there is any
possible association
lowess Adiponectin_P3 Sleep_time_weekday
lowess Adiponectin_Se_P3 Sleep_time_weekday
*(Above No Association)
lowess UCN3_Se_P3 Sleep_time_weekday
*(Suggests some sort of association with sleep time weekday)


*****Rsidual analysis to check for outliers, normality of
distribution, homoscadesticity and nonlinearity
*generate a new variable named `res', that contains the residuals
predict res,residuals
label variable res "Residuals"
* generate a new variable named `Adiponectin_Se_P3_hat', that contains
the predicted means
predict Adiponectin_Se_P3_hat, xb
label variable Adiponectin_Se_P3_hat "predicted (mean) outcome"
* generate diagnostic plots using the residuals
histogram res
graph box res
scatter res Adiponectin_Se_P3_hat


*Third: UCN3 gene:
tab UCN3_Se_P3
sum UCN3_Se_P3, detail
gen UCN3_Se_P3_bin = 0 if UCN3_Se_P3 <= 2.55
replace UCN3_Se_P3_bin = 1 if UCN3_Se_P3 > 2.55 & UCN3_Se_P3 <= 41.97
tab UCN3_Se_P3_bin
```

```
gen UCN3_Se_P3_n =.
replace UCN3_Se_P3_n = 0 if UCN3_Se_P3_bin == 0
replace UCN3_Se_P3_n = 1 if UCN3_Se_P3_bin == 1
label define UCN3_Se_P3_bin 0 "<=50%" 1 ">50%"
label values UCN3_Se_P3_n UCN3_Se_P3_bin
tab UCN3_Se_P3_n
stepwise, pr(.10): regress logUCN3_Se_P3 logAdiponectin_P3
logAdiponectin_Se_P3 School sex_n AgeP3 med_hist_n medical_history_n
meds_n mom_diab_n dad_diab_n mom_edu_n mother_ed_n dad_edu_n fath_ed_n
sleep_weekday_n wakeup_weekday_n Sleep_time_weekday_n sleep_weekend_n
wakeup_weekend_n Sleep_time_weekend_n interupted_sleep_n
interupted_sleep_bi_n wakeup_night_n wakeupnight_bin_n sleepy_day_n
sleepy_day_bi_n hard_wakeup_n hard_wakeup_bi_n diff_fall_asleep_n
diff_fall_asleep_bi_n quality_sleep_n snore_n snore_bi_n nap_n
nap_bi_n sleep_apnea_max_n bed12bin_n bed10_bin_n sleep_duration6_n
sleep_duration7_n sleep_duration8_n WHOcat3_n OBWHO3_n

regress logUCN3_Se_P3 Sleep_time_weekday_n
regress logUCN3_Se_P3 Sleep_time_weekday_n sex_n fath_ed_n School
mom_diab_n medical_history_n physical_act OBWHO3_n

logit UCN3_Se_P3_n Sleep_time_weekday_n, or
logit UCN3_Se_P3_n Sleep_time_weekday_n mother_ed_n mom_diab_n
physical_act OBWHO3_n electronic_n medical_history_n snore_n nap_n, or

regress logUCN3_Se_P3 bed12bin_n
regress logUCN3_Se_P3 bed12bin_n mom_diab_n mother_ed_n
medical_history_n School OBWHO3_n dinner

logit UCN3_Se_P3_n bed12bin_n, or
logit UCN3_Se_P3_n bed12bin_n AgeP3 dad_diab_n electronic_n snore_n
nap_n , or

regress logUCN3_Se_P3 sleep_duration6_n


*Fourth: oral findings: none was statistically significant
poisson NteethP2 Sleep_time_weekday_n, irr
poisson NteethP2 Sleep_time_weekday_n physical_act electronic_n
poisson NteethP2 bed12bin_n
poisson NteethP2 sleep_duration6_n
poisson NteethP2 logAdiponectin_P3
poisson NteethP2 logAdiponectin_Se_P3
poisson NteethP2 logUCN3_Se_P3

regress RedPctP2 Sleep_time_weekday_n
regress RedPctP2 bed12bin_n
regress RedPctP2 sleep_duration6_n
regress RedPctP2 logAdiponectin_P3
regress RedPctP2 logAdiponectin_Se_P3
```

```
regress RedPctP2 logUCN3_Se_P3

logit HiDecayP2 Sleep_time_weekday_n, or
logit HiDecayP2 bed12bin_n,or
logit HiDecayP2 sleep_duration6_n,or
logit Adiponectin_P3_75 HiDecayP2

poisson NteethP2 Adiponectin_P3_75
logit Adiponectin_P3_75 OBWHO3_n, or
logit Adiponectin_P3_75  Sleep_time_weekday_n


***** Now we want to build our linear regression model by checking the
association of each vraible with our outcome variable.
****Think of all possible confounders and Effect modifiers:
*Confounders for Adiponectin
AgeP3, sex_n, medical_history_n, mother_ed_n, fath_ed_n, electronic_n,
mom_diab_n, dad_diab_n
*Effect modification:
gen intOBEadipo = OBWHO3_n * Adiponectin_P3
gen intSLEPOBE = Sleep_time_weekday_n * OBWHO3_n
gen intSLEP12OBE= bed12bin_n * OBWHO3_n

**************************************************

* at 12 (midnight) By obesity
regress logAdiponectin_P3 bed12bin_n if OBWHO3_n == 0
*regress logAdiponectin_P3 bed12bin_n if OBWHO3_n == 1
regress logAdiponectin_P3 bed12bin_n if ow_ob == 0
*regress logAdiponectin_P3 bed12bin_n if ow_ob == 1


logistic Adiponectin_P3_75 bed12bin_n if OBWHO3_n == 0
*logistic Adiponectin_P3_75 bed12bin_n if OBWHO3_n == 1
logistic Adiponectin_P3_75 bed12bin_n if ow_ob == 0
*logistic Adiponectin_P3_75 bed12bin_n if ow_ob == 1


*regress logAdiponectin_Se_P3 bed12bin_n if OBWHO3_n == 0
regress logAdiponectin_Se_P3 bed12bin_n if OBWHO3_n == 1
*regress logAdiponectin_Se_P3 bed12bin_n if ow_ob == 0
regress logAdiponectin_Se_P3 bed12bin_n if ow_ob == 1


regress logIL6_P3 bed12bin_n if OBWHO3_n == 0
*regress logIL6_P3 bed12bin_n if OBWHO3_n == 1
regress logIL6_P3 bed12bin_n if ow_ob == 0
regress logIL6_P3 bed12bin_n if ow_ob == 1

regress IL6_Se_P3 bed12bin_n
```

```
*regress IL6_Se_P3 bed12bin_n if OBWHO3_n == 0
*regress IL6_Se_P3 bed12bin_n if OBWHO3_n == 1
regress IL6_Se_P3 bed12bin_n if ow_ob == 0
regress IL6_Se_P3 bed12bin_n if ow_ob == 1

*regress logHSCRPugml bed12bin_n
*regress logHSCRPugml bed12bin_n if OBWHO3_n == 0
*regress logHSCRPugml bed12bin_n if OBWHO3_n == 1
regress logHSCRPugml bed12bin_n if ow_ob == 0
regress logHSCRPugml bed12bin_n if ow_ob == 1


drop permFP1
drop permFP2
drop permDP1
drop PermDP2
drop DFPctP1
drop DFPctP2
drop DPctP1
drop HiDecayP1
drop RedPctP1
drop NteethP1
drop permteethP1
drop decidteethP1

tab PermteethP2
sum PermteethP2,detail

hist Adiponectin_P1
graph box Adiponectin_P1
hist Adiponectin_P2
graph box Adiponectin_P2
hist PermteethP2
graph box PermteethP2
*Above is skewed to the left

*for continuous by categorical variable:
tabstat RedPctP2,  s(n mean SD) c(s) by(Adiponectin_P3_n)
tabstat AgeP3, s(n mean SD) c(s) by(Adiponectin_P3_n)

tab Adiponectin_P2
sum Adiponectin_P2, detail
gen Adiponectin_P2_bin = 0 if Adiponectin_P2 <= 6663.6
replace Adiponectin_P2_bin = 1 if Adiponectin_P2 > 6663.6 &
Adiponectin_P2 <= 48594.43
tab Adiponectin_P2_bin
gen Adiponectin_P2_n =.
replace Adiponectin_P2_n = 0 if Adiponectin_P2_bin == 0
replace Adiponectin_P2_n = 1 if Adiponectin_P2_bin == 1
label define Adiponectin_P2_bin 0 "<=50%" 1 ">50%"
```

```
label values Adiponectin_P2_n Adiponectin_P2_bin
tab Adiponectin_P2_n

hist NteethP2
gen logNteethP2 = log(NteethP2)
poisson logNteethP2 bed12bin
poisson logNteethP2 Sleep_time_weekday

logistic HiDecayP2 bed12bin
logistic HiDecayP2 Sleep_time_weekday
logistic HiDecayP2 IL6_P3 sex_n
logistic HiDecayP2 IL6_Se_P3

regress HSCRPugml Abscent_circadian sex_n i.School  AgeP3 nap_bi_n
i.medical_history_n DineOut, robust
regress IL6_Se_P3 Abscent_circadian sex_n i.School  AgeP3 nap_bi_n
i.medical_history_n DineOut, robust
regress logIL6_P3 Abscent_circadian sex_n i.School  AgeP3 nap_bi_n
i.medical_history_n DineOut, robust


*Only Gene:
sum UCN3_Se_P3, detail

drop crp_hb1c
drop hba1c_bi
drop hba1c_57
drop ob_crp
drop ob_crp_bi
drop nap_hours
drop nap_hours_
drop RecordNo
drop tea
drop milk
drop freq_milk
drop flavored_milk
drop Breakfat
drop Family_meal
drop eAG
drop IFCCHBA1C
drop RECALLforfullprofile
drop HSCRPngml
drop fried_bi
drop daysnack_bi
drop friedfood
drop Eat_TV
drop Eat_Restaurant
drop Fast_Foods
drop Snacks
drop Milk_All
```

```
drop EatHome
drop EatHomeper
drop health_prob
drop Medicine
drop HBA1C
drop SysBP1
drop DiaBP1
drop SysBP2
drop DiaBP2
drop HIBP1
drop HIBP2
drop frozen_yogurt
**********************************************************************
**********
*** Biomarkers:
tab Adiponectin_P3
sum Adiponectin_P3,detail
gen Adiponectin_P3_bin = 0 if Adiponectin_P3 <= 4993.21
replace Adiponectin_P3_bin = 1 if Adiponectin_P3 > 4993.21 &
Adiponectin_P3 <= 154642.5
tab Adiponectin_P3_bin
gen Adiponectin_P3_n =.
replace Adiponectin_P3_n = 0 if Adiponectin_P3_bin == 0
replace Adiponectin_P3_n = 1 if Adiponectin_P3_bin == 1
label define Adiponectin_P3_bin 0 "<=50%" 1 ">50%"
label values Adiponectin_P3_n Adiponectin_P3_bin
tab Adiponectin_P3_n
sum Adiponectin_P3, detail
gen Adiponectin_P3_75 =.
replace Adiponectin_P3_75 = 0 if Adiponectin_P3 <= 9321.74
replace Adiponectin_P3_75 = 1 if Adiponectin_P3 > 9321.74 &
Adiponectin_P3 <= 154642.5
label define Adiponectin_P3 0 " equal or less than 75%" 1 " more than
75%"
label value  Adiponectin_P3_75 Adiponectin_P3
tab  Adiponectin_P3_75

tab Adiponectin_Se_P3
sum Adiponectin_Se_P3,detail
gen Adiponectin_Se_P3_bin = 0 if Adiponectin_Se_P3 <= 7708450
replace Adiponectin_Se_P3_bin = 1 if Adiponectin_Se_P3 > 7708450 &
Adiponectin_Se_P3 <= 9.03e+07
tab Adiponectin_Se_P3_bin
gen Adiponectin_Se_P3_n =.
replace Adiponectin_Se_P3_n = 0 if Adiponectin_Se_P3_bin == 0
replace Adiponectin_Se_P3_n = 1 if Adiponectin_Se_P3_bin == 1
label define Adiponectin_Se_P3_bin 0 "<=50%" 1 ">50%"
label values Adiponectin_Se_P3_n Adiponectin_P3_bin
tab Adiponectin_Se_P3_n
```

```
tab IL6_Se_P3
sum IL6_Se_P3, detail
gen IL6_Se_P3_bi = 0 if IL6_Se_P3 <= 1.202366
replace IL6_Se_P3_bi = 1 if IL6_Se_P3 > 1.202366 & IL6_Se_P3 <= 2.92
tab IL6_Se_P3_bi
label define IL6_Se_P3 0 "<=50%mean" 1 ">50%mean"
label values IL6_Se_P3_bi IL6_Se_P3
tab IL6_Se_P3_bi


* Generating the log10 for the continuous outcomes==> not normally
distributed
gen logAdiponectin_P3 = log(Adiponectin_P3)
gen logAdiponectin_P3_n = log(Adiponectin_P3_n)
gen logAdiponectin_Se_P3 = log(Adiponectin_Se_P3)
gen logUCN3_Se_P3 = log(UCN3_Se_P3)
gen logIL8_P3 = log(IL8_P3)
gen logIL8_Se_P3 = log(IL8_Se_P3)
gen logLeptin_P3 = log(Leptin_P3)
gen logLeptin_Se_P3 = log(Leptin_Se_P3)
gen logVEGF_Se_P3 = log(VEGF_Se_P3)
gen logInsulin_P3 = log(Insulin_P3)
gen logInsulin_Se_P3 = log(Insulin_Se_P3)
gen logCRP_P3 = log(CRP_P3)
gen logMCP1_P3 = log(MCP1_P3)
gen logMCP1_Se_P3 = log(MCP1_Se_P3)


*By Adiponectin binary variable (by median)
*for 2 categorical variables:
tab Adiponectin_P3_n bed12bin_n, chi2 exact
tab Adiponectin_P3_n bed12bin_n, column nofreq chi2
tab Adiponectin_P3_n sex_n, chi2 exact
tab Adiponectin_P3_n sex_n, column nofreq chi2
tab Adiponectin_P3_n medical_history_n, chi2 exact
tab Adiponectin_P3_n medical_history_n, column nofreq chi2
tab Adiponectin_P3_n electronic_n, chi2 exact
tab Adiponectin_P3_n electronic_n, column nofreq chi2
tab Adiponectin_P3_n OBWHO3_n, chi2 exact
tab Adiponectin_P3_n OBWHO3_n, column nofreq chi2
tab Adiponectin_P3_n physical_act, chi2 exact
tab Adiponectin_P3_n physical_act, column nofreq chi2
tab Adiponectin_P3_n dinner, chi2 exact
tab Adiponectin_P3_n dinner, column nofreq chi2
tab Adiponectin_P3_n nap_n, chi2 exact
tab Adiponectin_P3_n nap_n, column nofreq chi2
tab Adiponectin_P3_n snore_n, chi2 exact
tab Adiponectin_P3_n snore_n, column nofreq chi2

*Bivariate for bedtime on Weekdays (BINARY)
```

```
tab bed12bin_n
tab sex_n bed12bin_n, chi2 exact
tab sex_n bed12bin_n, column nofreq chi2 exact
tab WHOcat3_n bed12bin_n, chi2 exact
tab WHOcat3_n bed12bin_n, column nofreq chi2 exact
tab OBWHO3_n bed12bin_n, chi2 exact
tab OBWHO3_n bed12bin_n, column nofreq chi2 exact
tab nap_bi_n bed12bin_n, chi2 exact
tab nap_bi_n bed12bin_n, column nofreq chi2 exact
tab snore_bi_n bed12bin_n, chi2 exact
tab snore_bi_n bed12bin_n, column nofreq chi2 exact
tab electronic_n bed12bin_n, chi2 exact
tab electronic_n bed12bin_n, column nofreq chi2 exact
tab medical_history_n bed12bin_n, chi2 exact
tab medical_history_n bed12bin_n, column nofreq chi2 exact
tab interupted_sleep_bi_n bed12bin_n, chi2 exact
tab interupted_sleep_bi_n bed12bin_n, column nofreq chi2 exact
tab wakeupnight_bin_n bed12bin_n, chi2 exact
tab wakeupnight_bin_n bed12bin_n, column nofreq chi2 exact
tab diff_fall_asleep_bi_n bed12bin_n, chi2 exact
tab diff_fall_asleep_bi_n bed12bin_n, column nofreq chi2 exact
tab sleepy_day_bi_n bed12bin_n, chi2 exact
tab sleepy_day_bi_n bed12bin_n, column nofreq chi2 exact
tab hard_wakeup_bi_n bed12bin_n, chi2 exact
tab hard_wakeup_bi_n bed12bin_n, column nofreq chi2 exact
tab Abscent_circadian bed12bin_n, chi2 exact
tab Abscent_circadian bed12bin_n, column nofreq chi2 exact
tab sleepdeptcat bed12bin_n, chi2 exact
tab sleepdept bed12bin_n, column nofreq chi2 exact
tab social_getlag bed12bin_n, chi2 exact
tab social_getlag bed12bin_n, column nofreq chi2 exact

*Bivariate for bedtime on Weekdays (CATEGORICAL)
tab bedtimeWDcat
tab sex_n bedtimeWDcat, column freq chi2 exact
tab WHOcat3_n bedtimeWDcat, column freq chi2 exact
tab OBWHO3_n bedtimeWDcat, column freq chi2 exact
tab nap_bi_n bedtimeWDcat, column freq chi2 exact
tab snore_bi_n bedtimeWDcat, column freq chi2 exact
tab interupted_sleep_bi_n bedtimeWDcat, column freq chi2 exact
tab hard_wakeup_bi_n bedtimeWDcat, column freq chi2 exact
tab diff_fall_asleep_bi_n bedtimeWDcat, column freq chi2 exact
tab sleepy_day_bi_n bedtimeWDcat, column freq chi2 exact
tab wakeupnight_bin_n bedtimeWDcat, column freq chi2 exact
tab electronic_n bedtimeWDcat, column freq chi2 exact
tab medical_history_n bedtimeWDcat, column freq chi2 exact
tab sleepdeptcat bedtimeWDcat, column freq chi2 exact
tab sleepdeptsevere bedtimeWDcat, column freq chi2 exact
tab socialgetlag_bi bedtimeWDcat, column freq chi2 exact
tab Abscent_circadian bedtimeWDcat, column freq chi2 exact
```

```
tab physical_act bedtimeWDcat, column freq chi2 exact
tab coffee_n bedtimeWDcat, column freq chi2 exact
tab dinner bedtimeWDcat, column freq chi2 exact
tab lunch bedtimeWDcat, column freq chi2 exact
tab friedfood_restaurant bedtimeWDcat, column freq chi2 exact


*for continuous by categorical variable:(WEKDAYS)
tabstat AgeP3,  s(n mean SD) c(s) by(bedtimeWDcat)
tabstat BMI3,  s(n mean SD) c(s) by(bedtimeWDcat)
tabstat Waist_cm3,  s(n mean SD) c(s) by(bedtimeWDcat)
tabstat Adiponectin_P3,  s(n mean SD) c(s) by(bedtimeWDcat)
tabstat Adiponectin_Se_P3,  s(n mean SD) c(s) by(bedtimeWDcat)
tabstat Leptin_P3,  s(n mean SD) c(s) by(bedtimeWDcat)
tabstat Leptin_Se_P3,  s(n mean SD) c(s) by(bedtimeWDcat)
tabstat Insulin_P3,  s(n mean SD) c(s) by(bedtimeWDcat)
tabstat Insulin_Se_P3,  s(n mean SD) c(s) by(bedtimeWDcat)
tabstat IL6_P3,  s(n mean SD) c(s) by(bedtimeWDcat)
tabstat IL6_Se_P3,  s(n mean SD) c(s) by(bedtimeWDcat)
tabstat IL8_P3,  s(n mean SD) c(s) by(bedtimeWDcat)
tabstat IL8_Se_P3,  s(n mean SD) c(s) by(bedtimeWDcat)
tabstat IL10_P3,  s(n mean SD) c(s) by(bedtimeWDcat)
tabstat IL10_Se_P3,  s(n mean SD) c(s) by(bedtimeWDcat)
tabstat VEGF_P3,  s(n mean SD) c(s) by(bedtimeWDcat)
tabstat VEGF_Se_P3,  s(n mean SD) c(s) by(bedtimeWDcat)
tabstat MCP1_P3,  s(n mean SD) c(s) by(bedtimeWDcat)
tabstat MCP1_Se_P3,  s(n mean SD) c(s) by(bedtimeWDcat)
tabstat CRP_P3,  s(n mean SD) c(s) by(bedtimeWDcat)
tabstat CRP_Se_P3,  s(n mean SD) c(s) by(bedtimeWDcat)
tabstat HSCRPugml,  s(n mean SD) c(s) by(bedtimeWDcat)
tabstat DineOut,  s(n mean SD) c(s) by(bedtimeWDcat)
tabstat EatHome,  s(n mean SD) c(s) by(bedtimeWDcat)

tabstat AverageSleepDuration,  s(n mean SD) c(s) by(bedtimeWDcat)
tabstat Sleep_time_weekday_n,  s(n mean SD) c(s) by(bedtimeWDcat)
tabstat Sleep_time_weekend_n,  s(n mean SD) c(s) by(bedtimeWDcat)
tabstat bedtime_both2,  s(n mean SD) c(s) by(bedtimeWDcat)
tabstat bed_hour,  s(n mean SD) c(s) by(bedtimeWDcat)
tabstat bed_weekend,  s(n mean SD) c(s) by(bedtimeWDcat)
tabstat sleepdept,  s(n mean SD) c(s) by(bedtimeWDcat)
tabstat social_getlag,  s(n mean SD) c(s) by(bedtimeWDcat)


*Bivariate for bedtime on Average weekdays and wekends (BINARY)
tab bed12
tab sex_n bed12, column freq chi2 exact
tab WHOcat3_n bed12, column freq chi2 exact
tab OBWHO3_n bed12, column freq chi2 exact
tab nap_bi_n bed12, column freq chi2 exact
tab snore_bi_n bed12, column freq chi2 exact
```

```
tab electronic_n bed12, column freq chi2 exact
tab medical_history_n bed12, column freq chi2 exact
tab interupted_sleep_bi_n bed12, column freq chi2 exact
tab wakeupnight_bin_n bed12, column freq chi2 exact
tab diff_fall_asleep_bi_n bed12, column freq chi2 exact
tab sleepy_day_bi_n bed12, column freq chi2 exact
tab hard_wakeup_bi_n bed12, column freq chi2 exact
tab Abscent_circadian bed12, column freq chi2 exact
tab sleepdeptcat bed12, column freq chi2 exact
tab sleepdeptsevere bed12, column freq chi2 exact
tab socialgetlag_bi bed12, column freq chi2 exact
tab physical_act bed12, column freq chi2 exact
tab coffee_n bed12, column freq chi2 exact
tab dinner bed12, column freq chi2 exact
tab lunch bed12, column freq chi2 exact
tab friedfood_restaurant bed12, column freq chi2 exact

*for continuous by binary bedtime variable:(Average weekdays and
wekends)
tabstat AgeP3,  s(n mean SD) c(s) by(bed12)

*Pearson correlation
pwcorr CRP_P3 HSCRPugml, sig star(.05) obs
pwcorr IL6_P3 IL6_Se_P3, sig star(.05) obs
pwcorr IL8_P3 IL8_Se_P3, sig star(.05) obs
pwcorr IL10_P3 IL10_Se_P3, sig star(.05) obs
pwcorr VEGF_P3 VEGF_Se_P3, sig star(.05) obs
pwcorr MCP1_P3 MCP1_Se_P3, sig star(.05) obs
pwcorr Adiponectin_P3 Adiponectin_Se_P3, sig star(.05) obs
pwcorr Leptin_P3 Leptin_Se_P3, sig star(.05) obs
pwcorr Insulin_P3 Insulin_Se_P3, sig star(.05) obs


xtmixed HSCRPugml AverageSleepDuration sex_n AgeP3 nap_bi_n
i.medical_history_n vediogames DineOut, || School:
xtmixed HSCRPugml Sleep_time_weekday_n sex_n AgeP3 nap_bi_n
i.medical_history_n vediogames DineOut, || School:
xtmixed HSCRPugml Sleep_time_weekend_n sex_n AgeP3 nap_bi_n
i.medical_history_n vediogames DineOut, || School:

xtmixed HSCRPugml bedtime_both2 sex_n AgeP3 nap_bi_n
i.medical_history_n vediogames DineOut, || School:
xtmixed HSCRPugml bed_hour sex_n AgeP3 nap_bi_n i.medical_history_n
vediogames DineOut, || School:
xtmixed HSCRPugml bed_weekend sex_n AgeP3 nap_bi_n i.medical_history_n
vediogames DineOut, || School:

xtmixed HSCRPugml i.bedtimecat2 sex_n AgeP3 nap_bi_n
i.medical_history_n vediogames DineOut, || School:
*sig
```

```
xtmixed HSCRPugml i.bedtimeWDcat2 sex_n AgeP3 nap_bi_n
i.medical_history_n vediogames DineOut, || School:
*sig
xtmixed HSCRPugml i.bedtimeWEcat2 sex_n AgeP3 nap_bi_n
i.medical_history_n vediogames DineOut, || School:

xtmixed HSCRPugml i.sleepdeptcat sex_n AgeP3 nap_bi_n
i.medical_history_n vediogames DineOut, || School:
xtmixed HSCRPugml i.sleepdeptcat sex_n AgeP3 nap_bi_n
i.medical_history_n vediogames BMI3 DineOut, || School:

xtmixed HSCRPugml i.sleepdeptsevere sex_n AgeP3 nap_bi_n
i.medical_history_n vediogames DineOut, || School:
xtmixed HSCRPugml i.socialgetlag_bi sex_n AgeP3 nap_bi_n
i.medical_history_n vediogames DineOut, || School:


xtmixed HSCRPugml bed12new sex_n AgeP3 nap_bi_n i.medical_history_n
vediogames DineOut, || School:
xtmixed HSCRPugml bedtimecat sex_n AgeP3 nap_bi_n i.medical_history_n
vediogames DineOut, || School:
*sig
xtmixed HSCRPugml bed12 sex_n AgeP3 nap_bi_n i.medical_history_n
vediogames DineOut, || School:
*Significant
xtmixed HSCRPugml bed12bin_n sex_n AgeP3 nap_bi_n i.medical_history_n
vediogames DineOut, || School:
*significant
xtmixed HSCRPugml bed12_weekend_bi sex_n AgeP3 nap_bi_n
i.medical_history_n vediogames DineOut, || School:

xtmixed HSCRPugml i.bedtime123 sex_n AgeP3 nap_bi_n
i.medical_history_n vediogames DineOut, || School:
xtmixed HSCRPugml i.bedtimeWDcat sex_n AgeP3 nap_bi_n
i.medical_history_n vediogames DineOut, || School:
xtmixed HSCRPugml i.bedtimeWEcat sex_n AgeP3 nap_bi_n
i.medical_history_n vediogames DineOut, || School:

xtmixed HSCRPugml sleepdept sex_n AgeP3 nap_bi_n i.medical_history_n
vediogames DineOut, || School:
xtmixed HSCRPugml sleepdept sex_n AgeP3 nap_bi_n i.medical_history_n
vediogames DineOut, || School:

xtmixed HSCRPugml social_getlag sex_n AgeP3 nap_bi_n
i.medical_history_n vediogames DineOut, || School:
xtmixed HSCRPugml i.timeandduration sex_n AgeP3 nap_bi_n
i.medical_history_n vediogames DineOut, || School:
```

```
xtmixed IL10_P3 AverageSleepDuration sex_n AgeP3 nap_bi_n
i.medical_history_n vediogames DineOut, || School:
xtmixed IL10_P3 Sleep_time_weekday_n sex_n AgeP3 nap_bi_n
i.medical_history_n vediogames DineOut, || School:
xtmixed IL10_P3 Sleep_time_weekend_n sex_n AgeP3 nap_bi_n
i.medical_history_n vediogames DineOut, || School:

xtmixed IL10_P3 bedtime_both2 sex_n AgeP3 nap_bi_n i.medical_history_n
vediogames DineOut, || School:
xtmixed IL10_P3 bed_hour sex_n AgeP3 nap_bi_n i.medical_history_n
vediogames DineOut, || School:
xtmixed IL10_P3 bed_weekend sex_n AgeP3 nap_bi_n i.medical_history_n
vediogames DineOut, || School:

xtmixed IL10_P3 i.bedtimecat2 sex_n AgeP3 nap_bi_n i.medical_history_n
vediogames DineOut, || School:
xtmixed IL10_P3 i.bedtimeWDcat2 sex_n AgeP3 nap_bi_n
i.medical_history_n vediogames DineOut, || School:
xtmixed IL10_P3 i.bedtimeWEcat2 sex_n AgeP3 nap_bi_n
i.medical_history_n vediogames DineOut, || School:

xtmixed IL10_P3 i.bedtimecat2 sex_n AgeP3 nap_bi_n i.medical_history_n
vediogames BMI3 DineOut, || School:

xtmixed IL10_P3 i.sleepdeptcat sex_n AgeP3 nap_bi_n
i.medical_history_n vediogames DineOut, || School:
xtmixed IL10_P3 i.sleepdeptsevere sex_n AgeP3 nap_bi_n
i.medical_history_n vediogames DineOut, || School:
xtmixed IL10_P3 i.socialgetlag_bi sex_n AgeP3 nap_bi_n
i.medical_history_n vediogames DineOut, || School:

xtmixed IL10_P3 bed12new sex_n AgeP3 nap_bi_n i.medical_history_n
vediogames DineOut, || School:
xtmixed IL10_P3 bedtimecat sex_n AgeP3 nap_bi_n i.medical_history_n
vediogames DineOut, || School:

xtmixed IL10_P3 bed12 sex_n AgeP3 nap_bi_n i.medical_history_n
vediogames DineOut, || School:
xtmixed IL10_P3 bed12bin_n sex_n AgeP3 nap_bi_n i.medical_history_n
vediogames DineOut, || School:
xtmixed IL10_P3 bed12_weekend_bi sex_n AgeP3 nap_bi_n
i.medical_history_n vediogames DineOut, || School:

xtmixed IL10_P3 i.bedtime123 sex_n AgeP3 nap_bi_n i.medical_history_n
vediogames DineOut, || School:
xtmixed IL10_P3 i.bedtimeWDcat sex_n AgeP3 nap_bi_n
i.medical_history_n vediogames DineOut, || School:
xtmixed IL10_P3 i.bedtimeWEcat sex_n AgeP3 nap_bi_n
i.medical_history_n vediogames DineOut, || School:
```

```
xtmixed IL10_P3 social_getlag sex_n AgeP3 nap_bi_n i.medical_history_n
vediogames DineOut, || School:

xtmixed IL10_P3 i.timeandduration sex_n AgeP3 nap_bi_n
i.medical_history_n vediogames DineOut, || School:
xtmixed IL10_P3 sleepdept sex_n AgeP3 nap_bi_n i.medical_history_n
vediogames DineOut, || School:
```
